# Expression of the Tyrosine Hydroxylase Gene from Rat Leads to Oxidative Stress in Potato Plants

**DOI:** 10.3390/antiox9080717

**Published:** 2020-08-07

**Authors:** Kamil Kostyn, Aleksandra Boba, Anna Kostyn, Bartosz Kozak, Michał Starzycki, Anna Kulma, Jan Szopa

**Affiliations:** 1 Department of Genetics, Plant Breeding and Seed Production, Faculty of Life Sciences and Technology, Wroclaw University of Environmental and Life Sciences, pl. Grunwaldzki 24A, 50-363 Wroclaw, Poland; bartosz.kozak@upwr.edu.pl (B.K.); szopa@ibmb.uni.wroc.pl (J.S.); 2 Department of Genetic Biochemistry, Faculty of Biotechnology, University of Wroclaw, Przybyszewskiego 63, 51-148 Wroclaw, Poland; aleksandra.boba@uwr.edu.pl (A.B.); anna.kostyn@wp.pl (A.K.); anna.kulma@uwr.edu.pl (A.K.); 3 Institute of Genetics and Microbiology, Faculty of Biological Sciences, University of Wroclaw, Przybyszewskiego 63, 51-148 Wroclaw, Poland; 4 The Plant Breeding and Acclimatization Inst. (IHAR)—National Research Inst., Research Div, Poznan, ul. Strzeszyńska 36, 60-479 Poznan, Poland; michals@nico.ihar.poznan.pl

**Keywords:** catecholamine, tyrosine hydroxylase, *Solanum tuberosum*, reactive oxygen species, oxidative stress, antioxidants

## Abstract

Catecholamines are biogenic aromatic amines common among both animals and plants. In animals, they are synthesized via tyrosine hydroxylation, while both hydroxylation or decarboxylation of tyrosine are possible in plants, depending on the species, though no tyrosine hydroxylase—a counterpart of the animal enzyme—has been identified yet. It is known that in potato plants, it is the decarboxylation of tyrosine that leads to catecholamine production. In this paper, we present the effects of the induction of an alternative route of catecholamine production by introducing the tyrosine hydroxylase gene from rat. We demonstrate that an animal system can be used by the plant. However, it does not function to synthesize catecholamines. Instead, it leads to elevated reactive oxygen species content and a constant stress condition in the plant, which responds with elevated antioxidant levels and improved resistance to infection.

## 1. Introduction

**Figure 1:**
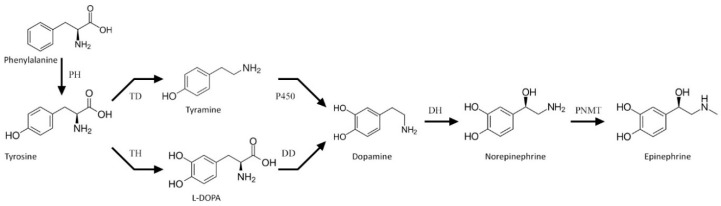
Scheme of the catecholamine biosynthesis pathway. PH—phenylalanine hydroxylase, TH—tyrosine hydroxylase, TD—tyrosine decarboxylase, DD—l-DOPA decarboxylase, DH—dopamine β-monooxygenase, and PMNT—phenylethanolamine *N*-methyltransferase.

Catecholamines are natural biogenic amines found in both animals and plants. Their biosynthesis route in animals has been well recognized. It starts with tyrosine hydroxylation to l-DOPA (levodopa, l-3,4-dihydroxyphenylalanine), catalyzed by tyrosine hydroxylase (TH, EC: 1.14.16.2). l-DOPA is further decarboxylated to dopamine by l-DOPA decarboxylase (DD, EC: 4.1.1.28). This neurotransmitter may undergo hydroxylation by dopamine β-monooxygenase (DH, EC: 1.14.17.1) to norepinephrine, and further to epinephrine by phenylethanolamine *N*-methyltransferase (PNMT, EC: 2.1.1.28). This main route of catecholamine biosynthesis may be complemented by that employing tyramine as a substrate for dopamine biosynthesis in a reaction driven by a cytochrome P450 isoform (CYP2D6 in human brain or CYP2D in rats) [1] (Figure 1). Functions of catecholamines in animals (hormones and neurotransmitters) are well recognized, as they have been studied extensively for decades [2].

The presence of these compounds in plants was shown as early as in the 1950s [3] and has been confirmed in a number of plant species since [4]. The role of catecholamines in plants is not fully recognized. Numerous publications report either on their structural or regulatory functions. They may be substrates for other relevant compounds such as betalains [5], alkaloids such as mescaline [6], melanins or hydroxycinnamic acid amides. Catecholamine conjugates with hydroxycinnamic acids are important molecules produced upon pathogen infection that may be incorporated into the cell wall, reinforcing its structure and providing a tighter barrier for the invading microorganisms [7]. Their role may also be explained by their antioxidant properties, since catecholamine molecules are potent free radical scavenging agents [8]. This activity is even more pronounced in their amide conjugates. Due to neighboring hydroxyl groups in the catechol ring, catecholamines possess remarkable ability to quench free radicals [9]. The 2,2-diphenyl-1-picrylhydrazyl (DPPH) and superoxide scavenging activities of catecholamines were shown to be much higher than those of ascorbic acid and comparable to those of catechin and flavan [10]. Moreover, catecholamines reacted with a single oxygen distinctively more strongly than catechin or sodium azide [11]. Dopamine was shown to be a stronger antioxidant than tocopherol and glutathione [8], and flavonoids—luteolin and quercetin—in vitro [12]. However, it must be noted that oxidized forms of catecholamines might be toxic and can pose a threat to cellular components. Analogously to their function in animals, catecholamines were suggested to control carbohydrate metabolism. In transgenic potatoes, they influenced simple sugar content, with a simultaneous decrease in starch [13,14]. Moreover, catecholamine interaction with phytohormones was reported. Catecholamines were shown to stimulate ethylene biosynthesis [15] or induce flowering in *Lemna paucicostata* [16]. Dopamine was indicated as a mediator of gibberellic acid activity in lettuce hypocotyl [17] and inhibited auxin oxidase in tobacco and *Acmella oppositifolia*, leading to growth stimulation [18].

Studies on the catecholamine biosynthesis pathway in plants showed their analogy to that found in animals. However, while the route through tyrosine hydroxylation is preferred in animals, both hydroxylation and decarboxylation of tyrosine was confirmed in plants, though not simultaneously, and the active route is probably species specific [4]. Tyrosine decarboxylase (TD) presence in plants has been established and its structure and functions are well recognized [19]. Plant TDs show narrow substrate specificity and can catalyze decarboxylation of tyrosine only or tyrosine and l-DOPA, depending on plant species and/or tissue and developmental stage [20,21]. However, despite the fact that the presence of l-DOPA was evidenced in numerous plants [22], tyrosine hydroxylase was not found in these organisms. Hydroxylation of tyrosine may be ascribed to other enzymes. For instance, tyrosinase catalyzes the reactions leading to melanin formation, namely the ortho-hydroxylation of monophenols to o-diphenols (monophenolase activity, EC 1.14.18.1) and the subsequent oxidation of o-diphenols to the corresponding *o*-quinones (diphenolase activity, EC 1.10.3.1). The l-DOPA produced in this pathway is utilized in the synthesis of DOPA-quinone rather than catecholamines [23]. However, l-DOPA may be decarboxylated to tyramine with the aid of tyrosine decarboxylase, which is further processed to yield catecholamines [4]. Nevertheless, tyrosine hydroxylase activity was found in callus cultures of *Portulaca grandiflora* and it was shown to be different from tyrosinase and polyphenol oxidase activities [24]. In another study, an enzyme of tyrosine hydroxylase activity was purified and characterized in *Mucuna pruriens* [25]. It was highly specific to tyrosine, but also showed a slight activity to tyramine, converting it to dopamine, although catalysis of such a reaction is usually ascribed to tyrosinases [26].

l-DOPA, the precursor for catecholamines, can also serve as a substrate for melanins, producing leucodopachrome and dopachrome either by auto-oxidation or due to polyphenol oxidase (PPO, EC 1.10.3.1) activity. This reaction produces reactive oxygen species (ROS), such as hydrogen peroxide, superoxide anion and hydroxyl radical [22]. l-DOPA, as with other non-protein amino acids, is accumulated in plants to serve as a deterrent to herbivores [27]. Moreover, it can act as allelochemical and, after exudation from the roots, leads to a reduction in growth of neighboring plants [28]. The toxicity of l-DOPA is clearly multisource. The already mentioned production of quinones is connected with ROS generation on the one hand, but it also leads to quinoprotein formation on the other hand. These proteins can be produced by a reaction between dopaquinone and the sulfhydryl groups of proteins and lead to negative effects such as enzyme deactivation, mitochondrial dysfunction, DNA fragmentation, and apoptosis [29,30]. In addition, it has been shown that l-DOPA may be incorporated to proteins via mimicking tyrosine or phenylalanine in respective tRNA synthesis [31]. Nevertheless, the ROS generated due to l-DOPA oxidation are the most serious problem for a plant, as they cause oxidative stress with a number of diverse effects. Therefore, a specialized machinery of ROS neutralization must be employed by the plants to cope with oxidative stress. This machinery is based on two main groups of agents—one employing enzymatic apparatus to neutralize reactive radicals and another using small antioxidant molecules. The enzymatic mechanisms include antioxidant enzymes, such as superoxide dismutase (SOD) (which converts O_2_^●−^ to H_2_O_2_), catalases and peroxidases (which remove H_2_O_2_). The non-enzymatic mechanisms of ROS removal include antioxidant molecules, such as ascorbic acid, glutathione, phenylpropanoids, carotenoids, and α-tocopherol. Reactive oxygen species are intrinsic element of numerous stress events and, acting as signaling molecules, trigger signal transduction pathways in plant response [12]. It is well known that ROS is the factor that precedes activation of salicylic acid (SA) signaling in plants responding to biotic and abiotic stresses [32]. Members of the TGA and WRKY transcription factor families involved in SA-mediated transcriptional regulation play a significant role in controlling the biosynthesis of antioxidants, for instance, phenylpropanoids in response to stress.

In this study, we show the effects of rat tyrosine hydroxylase gene expression in potato. We show that an induction of the alternative route of tyrosine metabolism toward catecholamines results in the seizure of the catecholamine biosynthesis. l-DOPA synthesized due to tyrosine hydroxylase (TH) activity undergoes oxidation with simultaneous ROS production, which generates a state of constant oxidative stress that forces the plant to counteract with activation of antioxidant machinery.

## 2. Materials and Methods

### 2.1. Plant Material

Potato plants (*Solanum tuberosum* L., cv. Desiree), obtained from “Saatzucht Fritz Lange KG” (Bad Schwartau, Germany), were grown in tissue culture on Murashige and Skoog (MS) medium [33] supplemented with 0.8% agar, 1% sucrose and 250 mg/L claforan under 16 h light (21 °C) and 8 h darkness (16 °C) cycle.

### 2.2. Construction of Transgenic Potato Lines

The cDNA encoding TH from *Rattus norvegicus* (EMBL/GenBank database accession no. M10244.1), was ligated in the sense orientation into the plant binary vector under the control of the 35S CaMV (Cauliflower Mosaic Virus) promoter and octopine synthase (OCS) terminator. The vector was introduced into the *Agrobacterium tumefaciens* strain C58C1:pGV2260 and the integrity of the plasmid was verified by restriction enzyme analysis. Young leaves of wild-type potato *S. tuberosum* L. cv. Desiree were transformed with *A. tumefaciens* by immersing leaf explants in bacterial suspension. *A. tumefaciens*-inoculated leaf explants were subsequently transferred to callus induction medium and shoot-regeneration medium. Transgenic plants were pre-selected by using PCR with the primers for the neomycin phosphotransferase (kanamycin resistance) gene on genomic DNA isolated from 3-week-old tissue-cultured plants as a template and then selected by means of PCR on cDNA as a matrix and *R. norvegicus TH*-specific primers and subsequently by Western blot analysis with antibodies specific to rat TH protein (Sigma-Aldrich, Saint Louis, MO, USA).

### 2.3. Western Blot Analysis

Proteins were extracted from frozen plant material using extraction buffer (100 mM 4-(2-hydroxyethyl)-1-piperazineethanesulfonic acid (HEPES)-NaOH, pH 7.4, 10 mM MgCl_2_, 1 mM EDTA, 1 mM EGTA, 20% (*v*/*v*) glycerol, 0.5 mM phenylmethylsulfonyl fluoride (PMSF), and 70 mM beta-mercaptoethanol) supplemented either with 0.1% or 1% (*v*/*v*) TritonX-100. The assessment of the expression of the rat TH gene by means of Western blot analysis using rabbit IgG anti-TH protein was conducted as described previously [34]. Briefly, solubilized protein was run on 12% (*w*/*v*) sodium dodecyl sulfate (SDS) polyacrylamide gels and blotted electrophoretically onto nitrocellulose membranes (Schleicher and Schuell, Maidstone, UK). Following transfer, the membrane was sequentially incubated with blocking buffer (5% (*w*/*v*) defatted dry milk), and then with antibody directed against the TH protein (1:5000 dilution). Formation and detection of immune complexes was performed with alkaline phosphatase-conjugated goat anti-rabbit IgG at a dilution of 1:1500.

### 2.4. Tyrosine Hydroxylase Activity Assay

Plants from in vitro culture were ground in liquid nitrogen to fine powder and 100 mg was used for protein extraction to buffer containing 100 mM HEPES pH 7.4, 20% glycerol, 0.4% Triton X-100, 10 mM MgCl_2_, 2mM ethylenediaminetetraacetic acid (EDTA), 2mM ethylene glycol-bis(β-aminoethyl ether)-N,N,N′,N′-tetraacetic acid (EGTA), 0.5 mM PMSF and 0.04% 2-mercaptoethanol. TH activity was determined by measurements of the amount of l-DOPA compounds. The assay mixture contained 100 mM acetate buffer pH 6.0, 1 mM (6R)-5,6,7,8-tetrahydrobiopterin dihydrochloride (Sigma-Aldrich, Saint Louis, MO, USA), 19500 U/mL catalase (Sigma-Aldrich, Saint Louis, MO, USA), 200 μM tyrosine (Sigma-Aldrich, Saint Louis, MO, USA) and the plant extract containing 100 μg of protein (determined by Bradford method). The reaction was carried out at 37 °C for 15 min and stopped by adding 60 μL of 1 M HCL with 0.4 mM EDTA. Then the assay mixture was lyophilized and methanol extracted for GC–MS analysis, as described [35]. TH activity was expressed in picokatals/mg protein in the extract. The blind samples were prepared in the same way, but the stopping solution was added before the onset of the reaction. The assay was performed in three independent repeats.

### 2.5. Isolation and Analysis of Phenolics

The frozen tissue (100 mg), comminuted in liquid nitrogen, was extracted to 1 mL of 0.1% HCl in methanol in an ultrasonic bath for 10 min and centrifuged at 12,000× *g*, 4 °C for 10 min. The procedure was repeated twice and the collected supernatants were merged, dried under nitrogen flow and re-suspended in 200 μL of methanol (free phenolic fraction). The remaining pellet was hydrolyzed in 2 M NaOH at 37 °C overnight. Then the pH was adjusted to 3 using concentrated HCl and two volumes of ethyl acetate were added and mixed thoroughly. Following centrifugation (12,000× *g*, 4 °C, 15 min), the organic phase was collected and the extraction to ethyl acetate was repeated two more times. All the collected volumes were merged, dried under nitrogen flow and re-suspended in 200 μL of methanol (bound phenolic fraction). The obtained extracts were analyzed in Ultra Performance Liquid Chromatography (UPLC) with photodiode array detector (PDA) 2996 and Quadrupole Time of Flight (QTOF) mass detector in six independent repeats (Waters Corp., Milford, MA, USA). An Acquity UPLC BEH C18 (2.1 × 100 mm, 1.7 μm, Waters Corp., Milford, MA, USA) column was used for compound separation. The mobile phase consisted of 0.1% (*v*/*v*) formic acid (solvent A) and acetonitrile (solvent B) and the flow was 95A:5B for 1 min, then linearly for 11 min to 70A:30B, for the next 4 min to 0A:100B and 1 min to 95A:5B at a rate of 0.4 mL/min. The column temperature was 25 °C. Absorbance was measured at the 210–500 nm range and mass spectra were registered in the range of 50–1000 Da under the following conditions: nitrogen flow 800 L/h, source temperature 70 °C, cone temperature 400 °C, capillary voltage 3.5 kV, cone voltage 40–60 V, and scan time 0.2 s [36]. Identification of compounds was based on comparison to the retention times, absorbance spectra and mass spectra of pure standards (Sigma-Aldrich, Saint Louis, MO, USA).

### 2.6. Gas Chromatography–Mass Spectrometry (GC–MS) Analysis of Metabolome

An amount of 150 mg of whole plant tissue ground in liquid nitrogen was extracted with methanol (12 mL/g FW). Samples were incubated at 70 °C for 15 min and centrifuged at room temperature (12,000× *g*, 10 min). Then they were supplemented with 1.5 mL H_2_O and extracted with 0.75 mL chloroform. The polar phase was dried and resolved in piridin/metoxyamine solution (20 mg/mL) and then incubated at 37 °C for 2 h. Samples were derivatized with *n*-methyl-*n*-trimethylsilyl trifluoroacetamide (MSTFA) and analyzed in GC–MS. A GC8000 gas chromatograph with an A2000 autosampler and Voyager Quadrupole Mass Spectrometer (Thermoquest, Rodano, Milan, Italy) was used. Samples were analyzed in six independent repeats. Chromatograms and mass spectra were analyzed with TagFinder software based on a mass spectra database (The Max Planck Institute of Molecular Plant Physiology (MPI-MP), Golm, Germany) [37,38,39]. Ribitol was used as the external standard.

### 2.7. Real-Time Polymerase Chain Reaction (RT-PCR)

Total RNA prepared from frozen plant material using a plant RNA purification kit (RNeasy Kit, QIAGEN, Hilden, Germany) was used for cDNA synthesis preceded by removal of residual genomic DNA with a DNase I kit (Invitrogen, Carlsbad, CA, USA) according to the manufacturer’s instructions. A high-capacity cDNA Reverse Transcription Kit (Thermo Fisher Scientific, Waltham, MA, USA) was used for reverse transcription of the RNA as stated in the manufacturer’s instructions. Transcript levels were determined with RT-PCR using StepOnePlus™ Real-Time OCR Systems (Applied Biosystems, Foster City, CA, USA) on the cDNA matrix in three independent repeats. Primers used in the reactions were designed in LightCycler^®^ Probe Design v2 (Roche, Basel, Switzerland) software (Supplementary File S1). As there are several homologs of the studied genes (Supplementary File S2), the most conserved regions were chosen for amplification. For the reactions, DyNAmo SYBR Green qPCR Kit (Thermo Fisher Scientific, Waltham, MA, USA) was used. The cDNA was dissolved 5 times prior to the experiment. The annealing temperature was 57 °C. Changes in the transcript levels of the examined genes were presented as relative quantification in regard to control. The elongation factor (EF1α, AB061263) gene was used as the reference gene, as it was shown to be the most stable upon stress conditions in potato [40].

### 2.8. Determination of Antioxidant Capacity

Methanol extracts (free phenolic fraction and bound phenolic fraction) were used for antioxidant potential determination with DPPH [41]. A volume of 6 μL of sample extract was added to 200 μL of 0.2 mM DPPH methanolic solution and incubated at room temperature in darkness for 15 min. A volume of 6 μL of methanol was used in the control and pure methanol was used as blank. All assays were performed in six independent repeats. Subsequently, absorbance at *λ* = 515 nm was measured. The antioxidant potential corresponded to the degree of free radical reduction and was presented as:

$$\left(1-\frac{sample\;A_{515}}{control\;A_{515}}\right)\times 100\%$$

### 2.9. Hydrogen Peroxide Assay

A total of 50 mg of 4-week-old plant green tissue ground in liquid nitrogen was used for the analysis. Each sample was supplemented with 200 μL of 20 mM phosphate buffer pH 6.5, mixed and centrifuged (4 °C, 12,000× *g*, 10 min). A volume of 50 μL of the supernatant was then used for the assay with Amplex Red Assay (Life Technologies) according to the manufacturer’s instructions. The absorbance was measured at *λ* = 560 nm, fluorescence at *λ_ex_* = 571 nm and at *λ_em_* = 585 nm [42]. The assay was performed in six independent repeats.

### 2.10. Treatment of Plants with L-DOPA

The 4-week-old potato plants from in vitro culture were subjected to 0.1 mg/mL l-DOPA treatment. The plants grown in glass jars (5 plants per a jar) were sprayed with 3 mL of L-DOPA and collected at 3, 6 and 12 h after the treatment.

### 2.11. Determination of Resistance to Pathogen

*Phytophtora infestans* (pathotype 33-IHAR PIB) was grown on solid PDA medium (3%) at 18 °C for 14 days in darkness. Next, rye caryopses sterilized by autoclave, were placed onto the well-grown mycelium (30 per a Petri dish) and were left for 7 days under the same conditions. The inoculum prepared in this manner was applied to a leaf of individual plant. The plants were grown in hydroponic culture in a grow chamber (12 h photoperiod, 15 °C during the day and 10 °C at night) on liquid B5 medium (diluted 10 times) [43]. The experiment was conducted for 14 days in five independent repeats and the degree of infection was assessed according to a bonitation scale (1–1.5—high resistance; 2–2.5—mediocre resistance; 3–3.5—weak resistance).

### 2.12. Statistical Analysis

All experiments were performed using at least three biological repeats. The results were presented as the mean values ± standard deviation. To assess the significance of changes, one-way ANOVA with Tukey’s post-hoc test or the Kruskal–Wallis test was used (Statistica, Dell, Round Rock, TX, USA). Independent Component Analysis (ICA) for the data obtained from GC–MS analysis was performed with MetaGeneAnalyse software (v1.7.1; http://metagenealyse.mpimpgolm.mpg.de) [44].

## 3. Results

### 3.1. Construction of Transgenic Potato Plants

For transgenic potato plants, the generation agrotransformation method was employed and resulted in 232 regenerants, which were further pre-selected for *TH* cDNA presence in genomic DNA by means of PCR (Supplementary File S3A). In 118 individuals, a positive signal was detected. Plants positively screened for the presence of the transgene in genomic DNA were subjected to a subsequent stage of selection—the assessment of the level of the introduced gene’s expression. Total RNA isolated from 3-week-old potato plants from in vitro culture was transcribed to cDNA, which served as a matrix in a PCR reaction with primers specific to the introduced transgene. Among 118 samples, 28 were verified as positive (Supplementary File S3B). The 28 plants were then analyzed for the presence of tyrosine hydroxylase protein. Total proteins were separated in acrylamide gel electrophoresis (SDS-PAGE) and transferred onto a nitrocellulose membrane, followed by incubation with antibodies specific to *R. norvegicus* tyrosine hydroxylase. Out of 28 plants, 18 were positively selected (Supplementary File S3C) for the presence of 56 kDa protein specifically recognized by anti-TH antibodies; and based on the results from transcript-level analysis and Western blotting, five transgenic lines were selected for further analyses (HTZ8, HTZ12, HTZ28, HTZ43 and HTZ53). Tyrosine hydroxylase activity was confirmed in these lines (Supplementary File S3D). The transgenic lines cultured under in vitro conditions did not differ in their phenotype from the control.

### 3.2. Metabolic Profile of the Transgenic Potato Plants

**Figure 2:**
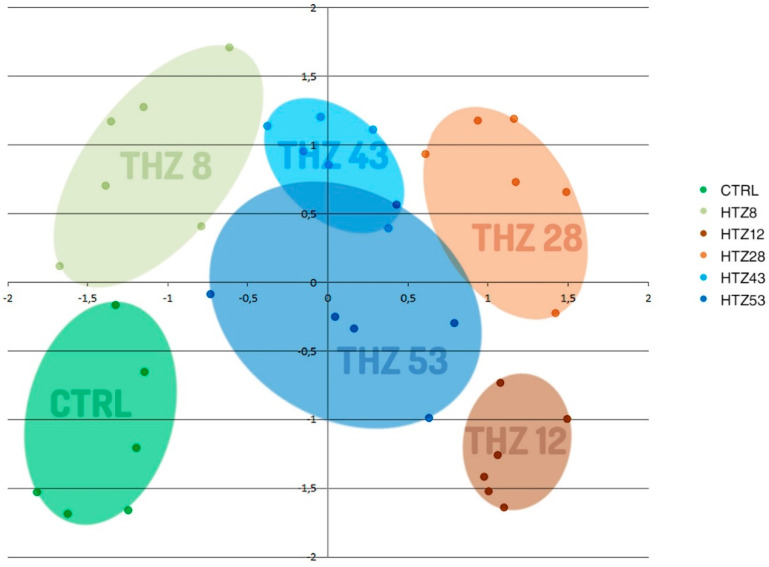
Independent Component Analysis of data obtained from Gas Chromatography–Mass Spectrometry (GC–MS) analysis of transgenic HTZ lines (HTZ8, HTZ12, HTZ28, HTZ43, HTZ53) and the control (CTRL). Colored areas cover agglomerations of the same sample’s data points.

To determine the influence of the rat tyrosine hydroxylase gene expression on metabolites, their contents were measured in methanolic extracts of selected transgenic lines (named HTZ lines) grown in in vitro culture by means of the GC–MS technique. A number of primary and secondary metabolites were identified. The obtained data were subjected to Independent Component Analysis (ICA). The HTZ transgenic lines clearly separated from the control and considering both the IC01 and IC02 components, line HTZ28 was the most different from the control (Figure 2).

### 3.3. Catecholamine Level in the Transgenic HTZ Lines

**Figure 3:**
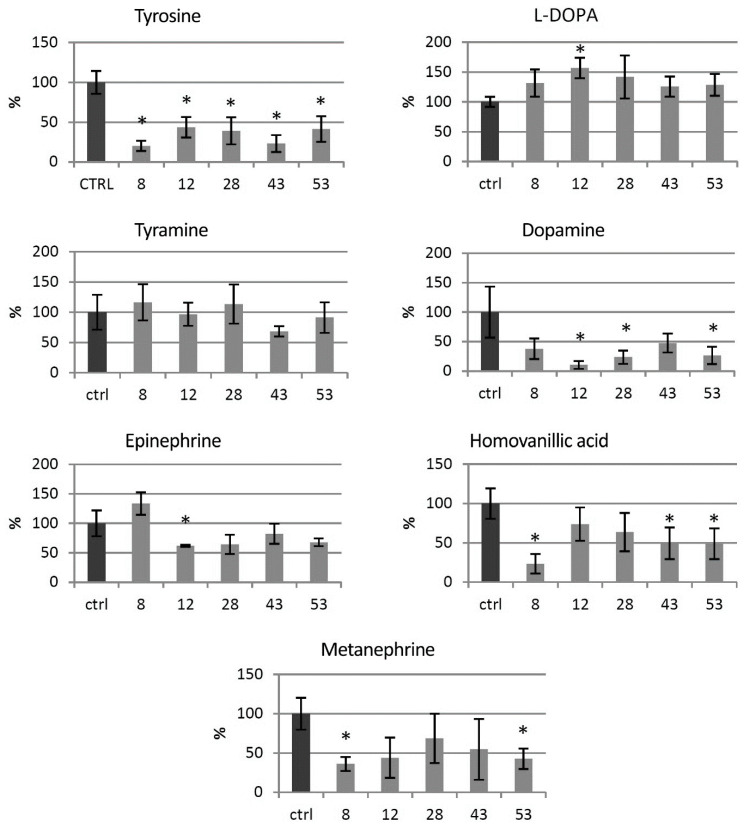
Levels of catecholamine group metabolites in HTZ transgenic lines relative to the control (non-transgenic potato) obtained with the GC–MS technique, presented as the means of six biological replicates ± standard deviation. Statistically significant changes (*p* < 0.05) are marked with asterisks.

Levels of catecholamine group metabolites were measured in the HTZ lines relative to the non-transgenic control (Figure 3). In all of the transgenic HTZ lines, the level of tyrosine, a substrate for tyrosine hydroxylase was considerably lower compared to the control. The level of l-DOPA, a direct product of tyrosine hydroxylase, was higher in all the transgenic lines, reaching 157% of the control. The level of tyramine did not change significantly in the HTZ lines, except in the HTZ43 line, where it was by 30% lower than in the control. A noticeable decrease in dopamine level was measured in the transgenic lines—to 10–48% of the control. Hydroxylation of dopamine at C-2 yields norepinephrine, which is further methylated to epinephrine. The level of the latter was lower in all transgenic lines but HTZ8 and the changes reached 62–82% of the control. The level of metanephrine, a catabolite of epinephrine was lower in all HTZ lines and reached 36–69% of the control. Homovanillic acid is a product of dopamine degradation. Its level was decreased in the transgenic lines to 36–74% of the control.

### 3.4. Primary Metabolite Level in the Transgenic HTZ Lines

The GC–MS technique allowed determining the levels of soluble primary metabolites with sugars, organic acids and amino acids among them. In all transgenic lines, a decrease in glucose, fructose and sucrose level was noted. The highest changes were observed for the line HTZ12, with decreases of 94%, 89.5% and 59.5%, respectively. In general, decreased levels of soluble sugar derivatives (sugar acids, sugar alcohols) were observed in all of the transgenic lines, except erythritol (elevated level in virtually all HTZ lines) (Supplementary File S4).

Among the primary metabolites, organic acids play an important role, as they participate in vital metabolic pathways, such as glycolysis and the Krebs cycle. The levels of these metabolites were found to be changed in the THZ transgenic lines. The most profound decreases were noted in all the transgenic lines for α-ketoglutarate (90% dropdown on average). The levels of citric acid and malic acid were also dropped down in all the transgenic lines but HTZ8 (Supplementary File S5).

Changes in free amino acids were also observed (Supplementary File S6). The highest decrease in the HTZ lines was found for proline and isoleucine. Leucine, valine, serine, asparagine and tryptophan levels dropped down to a lesser extent. The HTZ12 line showed the most diversity from the control, with a 3-fold increase in the arginine level and an 8-fold increase in the ornithine level.

### 3.5. Phenolic Level in the Transgenic HTZ Lines

**Figure 4:**
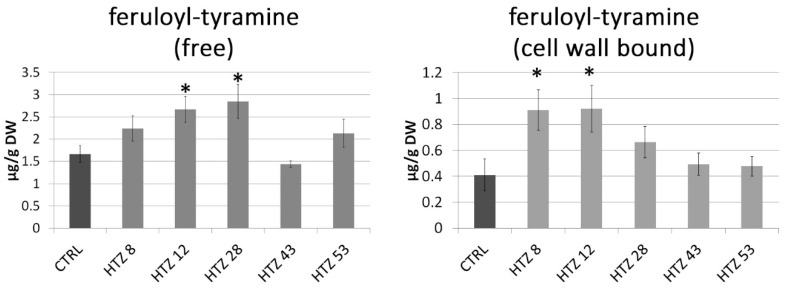
Amounts of free phenolic compounds in HTZ transgenic lines relative to the control (CTRL—non-transgenic potato) obtained with the LC–MS technique presented as the means of six biological replicates ± standard deviation. Statistically significant changes (*p* < 0.05) are marked with asterisks.

As the catecholamine synthetic route is connected with the large pathway of phenylalanine transformation, the phenylpropanoid pathway, levels of particular phenolic derivatives (both free and cell wall bound) were determined by means of the LC–MS technique. Based on retention times and absorption and mass spectra, the presence of feruloyl-tyramine, kaempferol glycoside and chlorogenic acid was determined in the methanolic extracts (free phenolic fraction). In addition, based on spectral analysis, putative ferulic acid derivative and caffeic acid derivative were measured. The amount of feruloyl-tyramine was higher in all HTZ lines except HTZ43 compared to the control (1.66 ± μg/g DW) (Figure 4). Similarly, the level of kaempferol glycoside was higher in all HTZ lines but HTZ43 in comparison to control (2.24 ± μg/g dry weight (DW)). A higher level of chlorogenic acid was found in HTZ8 and HTZ12 relative to control (8.78 ± μg/g DW). The levels of the ferulic acid derivative and caffeic acid derivative were higher in all the transgenic lines (Supplementary File S7).

In the cell wall-bound phenolic fraction, 4-hydroxybenzoic acid (4HBA), 4-hydroxybenzaldehyde (4HBAl), vanillin, *p*-coumaric acid, ferulic acid and feruloyl-tyramine were measured. In addition, based on spectral analysis, putative catechin derivative, *p*-coumaric acid derivative and tri-ferulic acid were identified. Levels of 4HBA and 4HBAl were elevated in all THZ lines (maximum of 2-fold) in comparison to the control (0.71 and 0.32 μg/g DW, respectively). Similarly, vanillin level was increased in the HTZ lines (except HTZ43—no change) relative to control (0.76 μg/g DW). Levels of *p*-coumaric acid, ferulic acid and feruloyl-tyramine were most increased in HTZ8 and HTZ12, while insignificant changes were measured in the remaining transgenic lines (Figure 4) (Supplementary File S7).

Total level of phenolic derivatives was higher in all the transgenic lines, in both methanolic extracts (free phenolics) and after hydrolysis (bound phenolics). The highest increase was noted for line HTZ28 and the smallest for line HTZ53 (Table 1).

### 3.6. Antioxidant Potential of the Transgenic Potato Extracts

**Figure 5:**
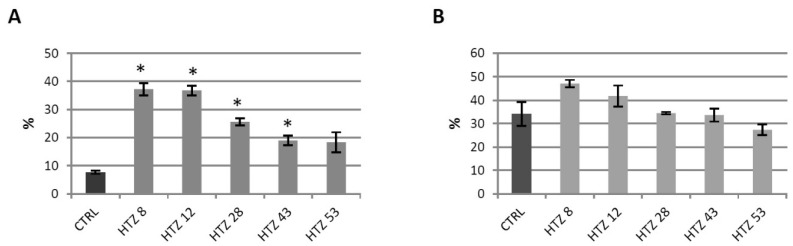
Antioxidant potential expressed as the percent of quenched 2,2-diphenyl-1-picrylhydrazyl (DPPH) radical measured in methanol extracts (**A**) and extracts after hydrolysis (**B**) from transgenic HTZ lines compared to the non-transgenic potato (CTRL), presented as the means of six biological replicates ± standard deviation. Statistically significant changes (*p* < 0.05) are marked with asterisks.

Phenolics are known to be strong antioxidants, thus their elevated level in the transgenic lines implies a higher antioxidant potential. The antioxidant potential of free phenolic extract and bound phenolic extract was measured using the DPPH method [41]. Methanol extracts from the transgenic plants were characterized with higher antioxidant potential (up to 5-fold) compared to control (Figure 5A). For cell wall hydrolysates, improved antioxidant potential was noted for lines HTZ8 and HTZ12, but the changes were weaker (Figure 5B).

### 3.7. Red-ox Analysis in the Transgenic HTZ Lines

#### 3.7.1. Analysis of H_2_O_2_ Level in Transgenic Potato Plants

**Figure 6:**
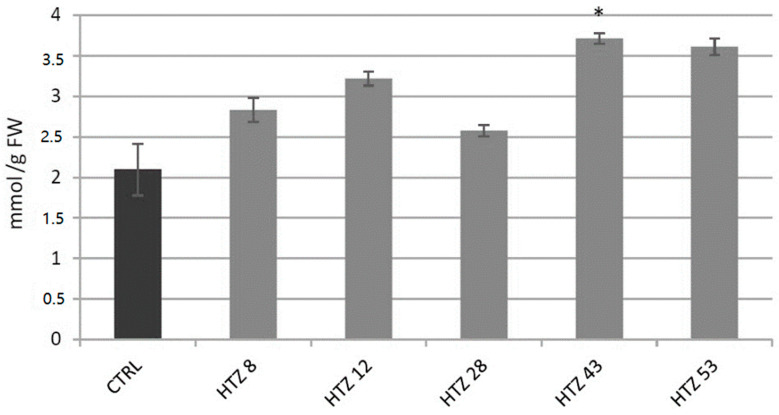
The level of H_2_O_2_ in HTZ potato lines compared to the non-transgenic potato (CTRL) presented as the means of six biological replicates ± standard deviation. Statistically significant changes (*p* < 0.05) are marked with asterisks.

Level of H_2_O_2_, a gauge of the red-ox state, was measured in the transgenic lines in relation to the control (Figure 6). The level of H_2_O_2_ was elevated in all HTZ lines by 44% on average. The highest increase was measured in HTZ43 and HTZ53 (by 77% and 72%, respectively).

#### 3.7.2. Analysis of Expression of Genes Involved in Free Radical Processing in Transgenic Potato Plants

**Figure 7:**
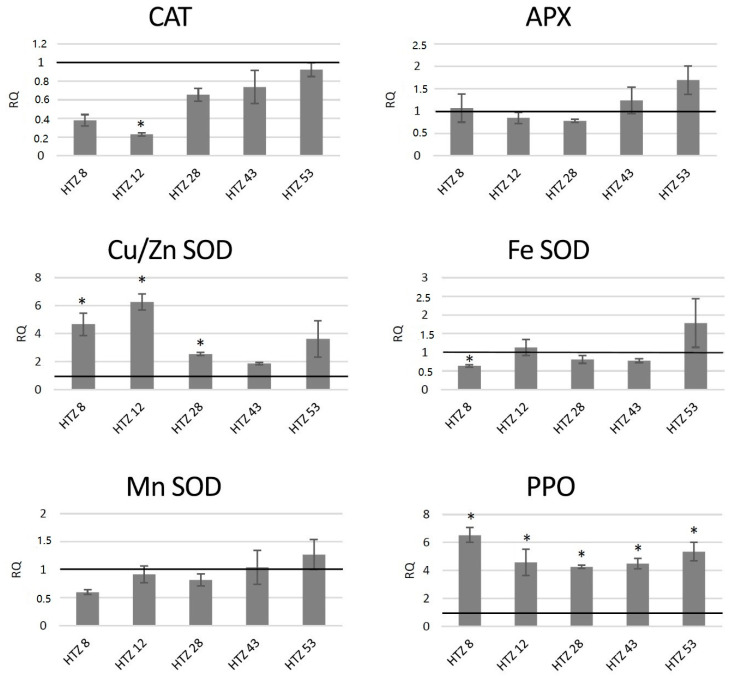
The level of transcript of genes involved in free radical processing (*CAT*—catalase, *APX*—ascorbate peroxidase, *SOD*—superoxide dismutase, and *PPO*—polyphenol oxidase) in the HTZ lines presented as relative quantification (RQ) in relation to the non-transgenic control (horizontal line at RQ = 1). The elongation factor gene was used as a reference gene. The results were obtained with the RT-qPCR method on the cDNA matrix as the mean values of three biological repeats ± standard deviation. Statistically significant changes (*p* < 0.05) are marked with asterisks.

The red-ox state depends mainly on the enzymatic system of free radical quenching, and therefore the levels of transcripts of catalase (*CAT*), ascorbate peroxidase (*APX*) and three superoxide dismutase (*SOD*) genes, which are involved in ROS neutralization were measured (Figure 7). For catalase gene expression, a dropdown in all transgenic lines was noted (by 42% on average), with the highest changes for HTZ8 and HTZ12 (by 62% and 77%, respectively). Ascorbate peroxidase expression did not change in the transgenic lines, except HTZ53, where it reached 177% of the control. Three superoxide dismutase (iron, copper/zinc and manganese) gene expression levels were measured. For one of them—*Cu/Zn SOD*—the average transcript level was considerably higher compared to the control (3.8-fold). The highest changes were detected for HTZ8 and HTZ12 (467% and 627% of the control, respectively). For *Fe SOD* and *Mn SOD*, no significant changes were observed, except HTZ8, where a decrease (by 36% and 41%, respectively) was detected. In addition, a polyphenol oxidase (*PPO*) gene expression level was measured. The PPO is responsible for *o*-hydroxylation of monophenols to *o*-diphenols (catechols) and further to *o*-quinones. The expression level of the *PPO* gene was substantially elevated in all the transgenic lines compared to the control (over 4- to 6-fold).

#### 3.7.3. Analysis of H_2_O_2_ Level in Potato Plants after Exogenous l-DOPA Treatment

**Figure 8:**
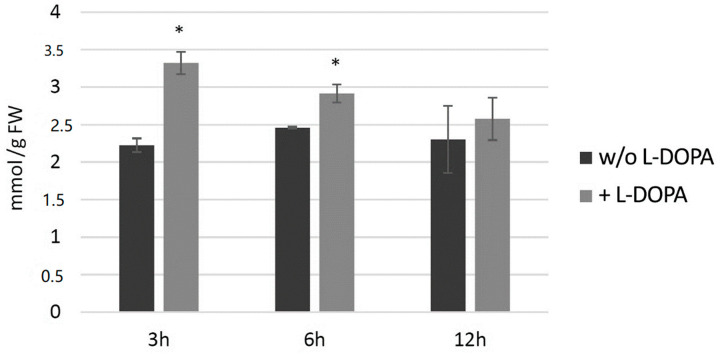
The level of H_2_O_2_ in wild-type potato plants treated with 0.1 mg/mL l-DOPA (brighter bars) compared to the non-treated control (darker bars) presented as the means of six biological replicates ± standard deviation. Statistically significant changes (*p* < 0.05) are marked with asterisks.

Potato plants (wild type) from in vitro culture were treated with 0.1 mg/mL l-DOPA and then H_2_O_2_ level and then measured at 3, 6 and 12 h later (Figure 8). The hydrogen peroxide level increased at all three time points in comparison to the control, with the highest change at 3 h after the treatment.

#### 3.7.4. Analysis of Expression of Genes Involved in Free Radical Processing in Potato Plants after Exogenous l-DOPA Treatment

**Figure 9:**
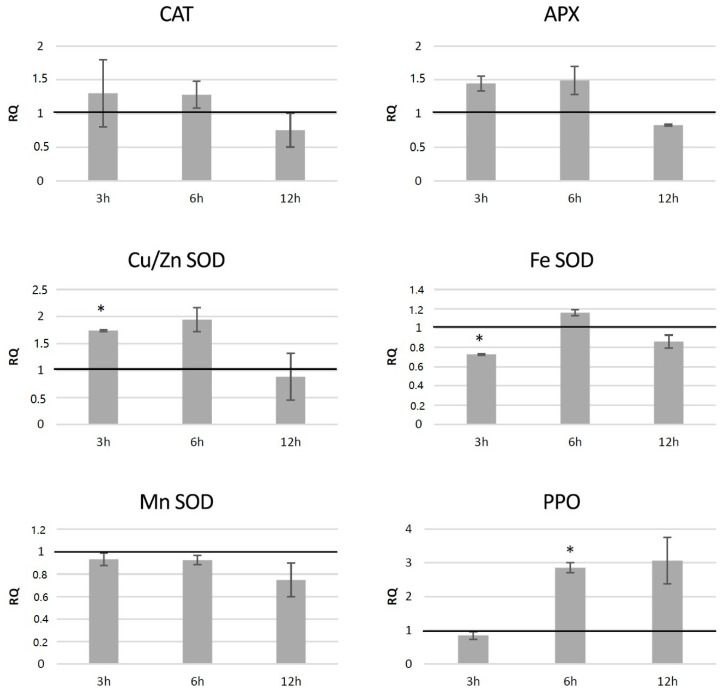
The level of transcript of genes involved in free radical processing (*CAT*—catalase, *APX*—ascorbate peroxidase, *SOD*—superoxide dismutase, and *PPO*—polyphenol oxidase) in wild-type potato plants treated with 0.1 mg/mL l-DOPA presented as relative quantification (RQ) in relation to the non-treated control (horizontal line at RQ = 1). The elongation factor gene was used as a reference gene. The results were obtained with the RT-qPCR method on the cDNA matrix as the mean values of three biological repeats ± standard deviation. Statistically significant changes (*p* < 0.05) are marked with asterisks.

In potato plants from in vitro culture treated with 0.1 mg/mL l-DOPA, the levels of transcripts of genes involved in free radical processing were measured (Figure 9). Transcript levels of genes connected with H_2_O_2_ neutralization were higher than in the control at 3 and 6 h after l-DOPA treatment—catalase by ca. 30% and ascorbate peroxidase by ca. 45%—and decreased below the control level at 12 h after the treatment. Among the superoxide dismutase genes, expression of the gene encoding the Cu/Zn SOD was higher at 3 and 6 h after the treatment—by 74% and 94% compared to the control, respectively. The polyphenol oxidase gene was considerably activated (ca. 3-fold) at 6 and 12 h after the treatment.

### 3.8. Resistance of the Transgenic Potatoes to Phytophtora Infestans Attack

**Figure 10:**
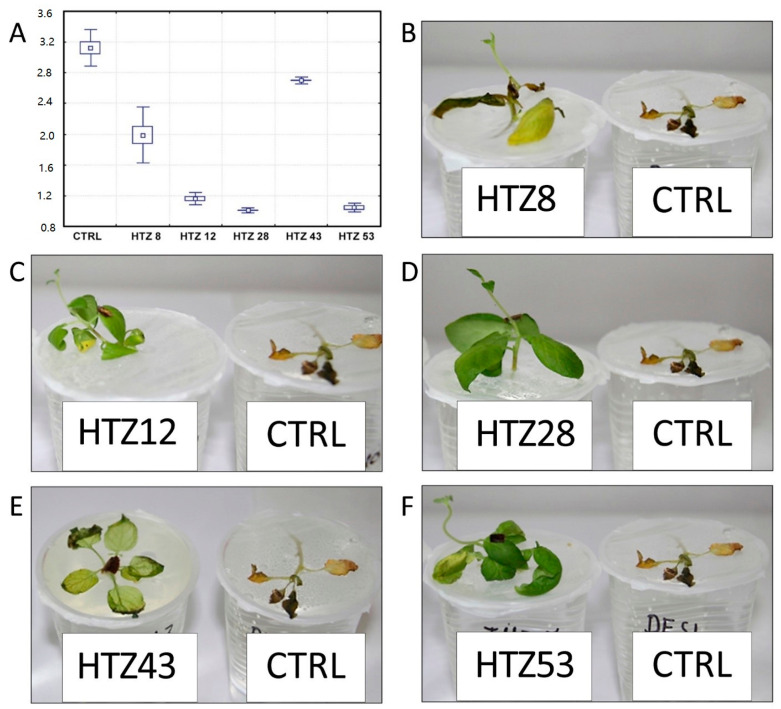
Degree of infection of transgenic HTZ lines with *P. infestans* in comparison to the non-transgenic potato (CTRL) (**A**). Sample photos of infected HTZ lines (**B**–**F**).

It is suggested that catecholamines participate in plant response to stress, including pathogen attack [21]. Moreover, the transgenic HTZ potato lines are characterized by a higher antioxidant potential, which is known to positively correlate with resistance to pathogens [45]. In order to investigate the effect of expression of tyrosine hydroxylase on potato susceptibility to infection, the HTZ lines were subjected to infection with *Phytophtora infestans*, a common and dangerous potato pathogen. Lines HTZ12, HTZ28 and HTZ53 were the most resistant in comparison to the control (Figure 10). No correlation was noted between the degree of infection and the phenolic compound level nor antioxidant potential.

## 4. Discussion

Although the literature on catecholamine biosynthesis in plants remains scant, it is generally believed that the main pathway proceeds through decarboxylation of tyrosine to tyramine conducted by tyrosine decarboxylase (TD). The presence of the ‘alternative pathway’ involving hydroxylation of tyrosine to l-DOPA by tyrosine hydroxylase (TH), which is the main route of catecholamine production in animals, was not fully confirmed in plants, although l-DOPA, a product of tyrosine hydroxylation, was found in these organisms. Based on the available plant genome sequence databases, no gene encoding tyrosine hydroxylase has been found. Analysis of protein sequence and protein domain similarity showed a maximum of 52% similar identity to other plant aromatic amino acid hydroxylases, with phenylalanine hydroxylase and tryptophan hydroxylase among them (Supplementary File S8B). In this study, using transgenic potato plants with the rat tyrosine hydroxylase gene, we analyzed the effect of the ‘alternative pathway’ of catecholamine synthesis on the plant’s metabolism. As anticipated, the obtained transgenic lines were characterized by increased l-DOPA levels, but despite further expectations, we observed decreased levels of dopamine and subsequent compounds of this route. In light of the results obtained for transgenic potatoes with overexpression of tyrosine decarboxylase, where the catecholamine level was significantly increased [13], the observed results suggest that the catecholamine biosynthesis route through tyrosine hydroxylation is non-functional in potato plants, while the induction of the l-DOPA synthesis by heterologous tyrosine hydroxylase reorganizes catecholamine biosynthesis and leads to redirection of the substrate towards the production of other types of compounds. It must be noted that TH shows some substrate promiscuity, with specificity constant (V max/K m) for phenylalanine and tryptophan being 10-fold lower and 30-fold lower than that for tyrosine, respectively [36]. However, hydroxylation of phenylalanine by TH was shown to produce L-DOPA, with free tyrosine as an intermediate [46,47].

It is known that l-DOPA is produced by some plants and utilized as an allelochemical [48,49] and its phytotoxicity is ascribed to the oxidative damage resulting from free radical species including ROS, generated from the pathway of l-DOPA metabolism (either auto-oxidation or oxidation aided by polyphenol oxidases (PPO)) to quinone and subsequently to melanins. Since we did not observe increased catecholamine synthesis after transformation of potato with the tyrosine hydroxylase gene in our research, we suspected that the surplus of l-DOPA might have resulted in its oxidation, followed by generation of free radicals, and consequently in oxidative stress in the plants. Indeed, we have observed an elevated level of H_2_O_2_ in the HTZ transgenic potato lines, which may translate to a constant oxidative stress present in the plants. This stays in agreement with Arabidopsis response to l-DOPA treatment, which led to changes in transcriptional activities of genes associated with the plant’s response to stress, both biotic and abiotic, with those involved in amino acid metabolism, oxidative stress, melanin synthesis and lignification among them [50]. Prolonged enhanced production of ROS can pose a threat to cells by causing the peroxidation of lipids, oxidation of proteins, damage to nucleic acids, etc. [51], thus it is imperative for an organism to maintain equilibrium between ROS and antioxidative capacity. In order to cope with oxidative stress, plants have developed several enzymatic and non-enzymatic approaches to eliminate free ROS in cells. The increased content of H_2_O_2_ in the HTZ potato lines correlated positively with the activation of the gene encoding the cytoplasmic superoxide dismutase (*SOD*). At the same time, the catalase (*CAT*) gene transcript level was lowered. It may reflect the mechanism, in which the transcription activity of the *CAT* gene decreases in plants during stress (e.g., pathogen infection), perhaps due to the H_2_O_2_ molecule itself [52] or accumulation of salicylic acid [53]. This helps in maintaining the H_2_O_2_ content at high levels, which in turn translates into activation of many antimicrobial activities [54,55]. The transgenic HTZ lines were also characterized with elevated levels of phenolic compounds and higher antioxidant potential. The phenolics comprise several groups of compounds such as flavonoids, phenolic acids, lignin or benzoates and have a whole gamut of functions, including structural, antioxidant, signaling and regulatory. Expression of the gene coding for phenylalanine ammonia lyase (*PAL*), which is considered a key enzyme of the phenolic synthesis, was increased in the transgenic HTZ lines. Bearing in mind a constant stress triggered by l-DOPA biosynthesis, this is not surprising, as *PAL* expression is known to increase upon ROS content increase and various stress conditions [56,57,58]. Our observation confirms the report by Soares et al. (2012) [59], where soybean which absorbed L-DOPA showed increased phenolic compounds, lignin content and activities of related enzymes such as PAL and cinnamyl alcohol dehydrogenase (CAD). Although lignin content in the 4-week-old plants from in vitro culture is barely determinable, we observed considerably high *CAD* gene and shikimate/quinate hydroxy-cinnamoyltransferase (*HCT*) gene transcript levels (Supplementary File S9). Increased expression of genes connected with lignification was also observed in l-DOPA-treated *Arabidopsis* [50]. Stress conditions, especially pathogen infections, coax plants to reinforce the cell wall with bio-reactive compounds to hinder microbial penetration into cells. Among the phenolics identified in the HTZ lines, feruloyl-tyramine deserves special attention as, on the one hand, it is a catecholamine derivative and, on the other hand, it participates in the plant’s response to pathogen infections [60], wounding [61] and drought [62]. The observed increase in the hydroxycinnamic acid-tyramine amide content in the transgenic lines may be somehow incomprehensible, because the level of tyramine, a direct substrate did not differ, nor was tyramine decarboxylase gene (*TD*) expression higher compared to the wild-type control. However, it is conceivable that tyrosine acts as the substrate for both tyrosine decarboxylase, normally occurring in the plant and for tyrosine hydroxylase. Because of the oxidative stress elicited by l-DOPA, the produced tyramine is immediately used in the reaction of the hydroxycinnamic acid amide biosynthesis driven by tyramine *n*-hydroxycinnamoyl transferase (THT), while dopamine synthesis is reduced. *THT* gene encoding this enzyme is known to be activated in the hypersensitivity reaction, due to salicylic acid (SA) action [63,64]. Analysis of the promoter sequence of the potato *THT* gene revealed the presence of a number of elements interacting with WRKY and MYB transcription factors, known to be connected with oxidative stress response [65,66]. Such elements were also found in the promoter sequences of *THT* from other plant organisms, such as tomato [67] or wheat [68]. Moreover, such promoter elements are found in a number of genes involved in the phenylpropanoid pathway, allowing for their control by SA in response to infection [69]. WRKY, among other transcription factor genes, were shown to be activated in *Arabidopsis* upon l-DOPA treatment [50]. This resemblance to a pathogen attack reaction in the HTZ potato lines might be the reason for their better resistance to *P. infestans*.

Another explanation might be rerouting of tyramine to the hydroxycinnamic acid amide production due to the presence of tyrosine hydroxylase (TH). TH is a soluble enzyme occurring in cytoplasm, but it has been shown to interact with plasma membrane elements, such as phosphatidylserine [70]. In animal neurons, TH is localized in the vicinity of the synaptic vesicle and mitochondria, together with aromatic amino acid decarboxylase (AAD) and dopamine β-hydroxylase (DH), which, thanks to the availability of substrates can swiftly convert l-DOPA to dopamine and dopamine to norepinephrine, respectively [71]. The co-localization of the three enzymes using an immunoprecipitation approach in rats was also shown by Cartier et al. [72]. To date, however, the presence of such complexes or similar complexes that could involve tyrosine decarboxylase remains hypothetical. However, the presence of tyrosine hydroxylase in transgenic potatoes could lead to the rearrangement of the putative enzymatic complex and tyramine might be rerouted to the biosynthesis of hydroxycinnamic acid amides catalyzed by THT. Nonetheless, this hypothesis requires detailed research on the interactions between the enzymes of catecholamine biosynthesis in plants.

## 5. Conclusions

To summarize, the study on the effect of the alternative route of tyrosine metabolism towards catecholamines led to acquisition of new knowledge on the possibility of manipulating plant metabolic pathways by introducing an animal-type system. Expression of the rat tyrosine hydroxylase gene in potatoes resulted in elevated l-DOPA levels. However, this intermediate did not serve for catecholamine biosynthesis, but was oxidized with polyphenol oxidases and possibly also spontaneously. These reactions produced ROS molecules, the elevated level of which led to the formation of constant oxidative stress conditions. To compensate, antioxidant machinery was actuated—both enzymatic and non-enzymatic. This in turn caused better resistance of the transgenic potatoes to *P. infestans* infection. We suspect that introduction of an external element to the mechanism of catecholamine synthesis in potato disturbed the process and caused redirection of the substrates. These results constitute a starting point to a new study, which will determine whether an enzymatic complex producing catecholamines exists or not.

## Figures and Tables

**Table 1:** Total phenolic content assayed in the transgenic HTZ lines in the methanolic extracts (free phenolics) and after hydrolysis (cell wall-bound phenolics) compared to the control (CTRL).

Potato Line	Free Phenolics [%]	Cell Wall-Bound Phenolics [%]
CTRL	100	100
HTZ8	212.3	148.5
HTZ12	194.6	145.3
HTZ28	249.7	169.4
HTZ43	198.5	137.4
HTZ53	132.5	107.5

## Supplementary Materials

The following are available online at https://www.mdpi.com/2076-3921/9/8/717/s1, Supplementary File S1. Details of primers used in RT-qPCR. Supplementary File S2. The number of selected gene homologues found in the potato genome. Supplementary File S3. Consecutive selection steps of potato plants transformed with TH gene from *R. norvegicus*. A—sample PCR product electrophoresis separation with use of primers specific to a 486-nt fragment of *TH* cDNA from *R. norvegicus*. P—positive control, N—negative control (non-transgenic potato); B—sample Northern blot of RNA samples from transgenic potato plants bearing *TH* gene from R. norvegicus. P—positive control, N—negative control (wt potato); C—Western blot analysis of proteins isolated from transgenic HTZ potato plants. N—negative control (non-transgenic potato); activity of the TH protein in extracts from the HTZ transgenic potato lines (HTZ 8, HTZ 12, HTZ 28, HTZ 43, HTZ 53) relatively to control (CTRL) expressed in pkatals calculated from the amount of L-DOPA produced from tyrosine. Supplementary File S4. Levels of soluble sugars in HTZ transgenic lines relatively to the control (non-transgenic potato) obtained with GC-MS technique presented as means of 6 biological replicates ± standard deviation. Statistically significant changes (*p* < 0.05) are marked with asterisks. Supplementary File S5. Levels of organic acids in HTZ transgenic lines relatively to the control (non-transgenic potato) obtained with GC-MS technique presented as means of 6 biological replicates ± standard deviation. Statistically significant changes (*p* < 0.05) are marked with asterisks. Supplementary File S6. Levels of soluble amino acids in HTZ transgenic lines relatively to the control (non-transgenic potato) obtained with GC-MS technique presented as means of 6 biological replicates ± standard deviation. Statistically significant changes (*p* < 0.05) are marked with red color. Supplementary File S7. Levels of soluble (A) and cell wall bound (B) phenolics’ derivatives in HTZ transgenic lines relatively to the control (non-transgenic potato) obtained with LC-MS technique presented as means of 6 biological replicates ± standard deviation. Statistically significant changes (*p* < 0.05) are marked with red color. Supplementary File S8. Similarity analysis (Blastp) of rat tyrosine hydroxylase against plant protein sequences (Swiss Prot Database). Supplementary File S8B. Protein domain similarity analysis using profile hidden Markov Models (hmmsearch). Supplementary File S9. The levels of transcripts of cinnamyl-alcohol dehydrogenase (*CAD*) and shikimate/quinate hydroxy-cinnamoyl transferase (*HCT*) genes in the HTZ lines presented as relative quantification (RQ) in relation to the control (horizontal line at RQ = 1). Elongation factor gene was used as a reference gene. The results were obtained with RT-qPCR method on cDNA matrix as mean values of 3 biological repeats ± standard deviation. Statistically significant changes (*p* < 0.05) are marked with asterisks.

## Abbreviations

APX	ascorbate peroxidase
CAD	cinnamyl alcohol dehydrogenase
CAT	catalase THT—n-hydroxycinnamoyl transferase
HCT	shikimate/quinate hydroxy-cinnamoyltransferase
PAL	phenylalanine ammonia lyase
PPO	polyphenol oxidase
SOD	superoxide dismutase
TD	tyrosine decarboxylase
TH	tyrosine hydroxylase
