# Supplementary material for: Expression of the Tyrosine Hydroxylase Gene from Rat Leads to Oxidative Stress in Potato Plants
Source: Antioxidants (Basel). 2020 Aug 7;9(8):717. doi: 10.3390/antiox9080717 (PMC7465045; doi:10.3390/antiox9080717)
Supplement: Supplementary file 1 [file antioxidants-09-00717-s001.zip › Supple/Supplemental.pdf]

NAME	PRIMER	%GC	Tm [°C]
Elongation factor (EF1α)	**FWD:** TACAAGATTGGTGGTATTGGAAC	39	61
	**REV:** CCAACATTGTCACCGGG	59	62
Polyphenol oxidase (PPO)	**FWD:** CCTTCAAAGTGAAAGTCCGAGA	45	63
	**REV:** GGGAATACCTGGCTTGC	59	62
Catalase (CAT)	**FWD:** CTGAGGATCTCTTGCCG	59	61
	**REV:** AGTAAATACCAGGGACGATATG	41	60
Superoxide dismutase Cu-Zn (SOD Cu-Zn)	**FWD:** TTACTATTACCGACAAGCAGATTC	38	61
	**REV:** GCTCATGTCCTCCCTTTC	56	61
Superoxide dismutase Fe (SOD Fe)	**FWD:** TTACTATTACCGACAAGCAGATTC	38	61
	**REV:** GCTCATGTCCTCCCTTTC	56	61
Superoxide dismutase Mn (SOD Mn)	**FWD:** TCTTCGGACTTTAGTGAGCAGA	45	64
	**REV:** GCCATAGTCGTATAGGGAGA	50	61
Ascorbate peroxidase (APX)	**FWD:** AAGGAGCGTTCTGGTTT	47	56
	**REV:** CTTGTCTGATGGCAACTGT	47	57
Tyrosine hydroxylase (TH)	**FWD:** ACTGCTGCCATGAGCTGTTGG	57	65
	**REV:** AGCTAATGGCACTCAGTGCGTG	55	65
Neomycin phosphotransferase II (NPTII)	**FWD:** CCGACCTGTCCGGTGCCC	78	67
	**REV:** CGCCACACCCAGCCGGCC	83	71

Supplementary File S1. Details of primers used in RT-qPCR.

CAT 3

SEQ_ID	Functional Annotation
PGSC0003DMT400075611	catalase
PGSC0003DMT400003986	catalase isozyme 2
PGSC0003DMT400025653	catalase isozyme 3

APX 8

SEQ_ID	Functional Annotation
PGSC0003DMT400009380	ascorbate peroxidase
PGSC0003DMT400077286	ascorbate peroxidase
PGSC0003DMT400015799	ascorbate peroxidase
PGSC0003DMT400004360	cytosolic ascorbate peroxidase
PGSC0003DMT400077268	cytosolic ascorbate peroxidase 1
PGSC0003DMT400009378	l-ascorbate peroxidase 1, cytosolic
PGSC0003DMT400082709	l-ascorbate peroxidase 1, cytosolic
PGSC0003DMT400002341	thylakoid-bound ascorbate peroxidase 6

PPO 11

SEQ_ID	Functional Annotation
PGSC0003DMT400048692	chloroplast polyphenol oxidase
PGSC0003DMT400048685	polyphenol oxidase
PGSC0003DMT400048699	polyphenol oxidase
PGSC0003DMT400048686	polyphenol oxidase
PGSC0003DMT400048684	polyphenol oxidase
PGSC0003DMT400076055	polyphenol oxidase
PGSC0003DMT400057781	polyphenol oxidase
PGSC0003DMT400048681	polyphenol oxidase
PGSC0003DMT400048703	polyphenol oxidase
PGSC0003DMT400048706	polyphenol oxidase b, chloroplastic
PGSC0003DMT400048679	polyphenol oxidase f, chloroplastic

SOD 7

SEQ_ID	Functional Annotation
PGSC0003DMT400027651	copper-zinc superoxide dismutase
PGSC0003DMT400046236	Superoxide dismutase
PGSC0003DMT400013447	Superoxide dismutase
PGSC0003DMT400070920	Superoxide dismutase
PGSC0003DMT400059417	Superoxide dismutase [Cu-Zn]
PGSC0003DMT400001102	Superoxide dismutase [Cu-Zn]
PGSC0003DMT400042937	Superoxide dismutase [Mn], mitochondrial

Supplementary File S2. The number of selected gene homologues found in the potato genome.

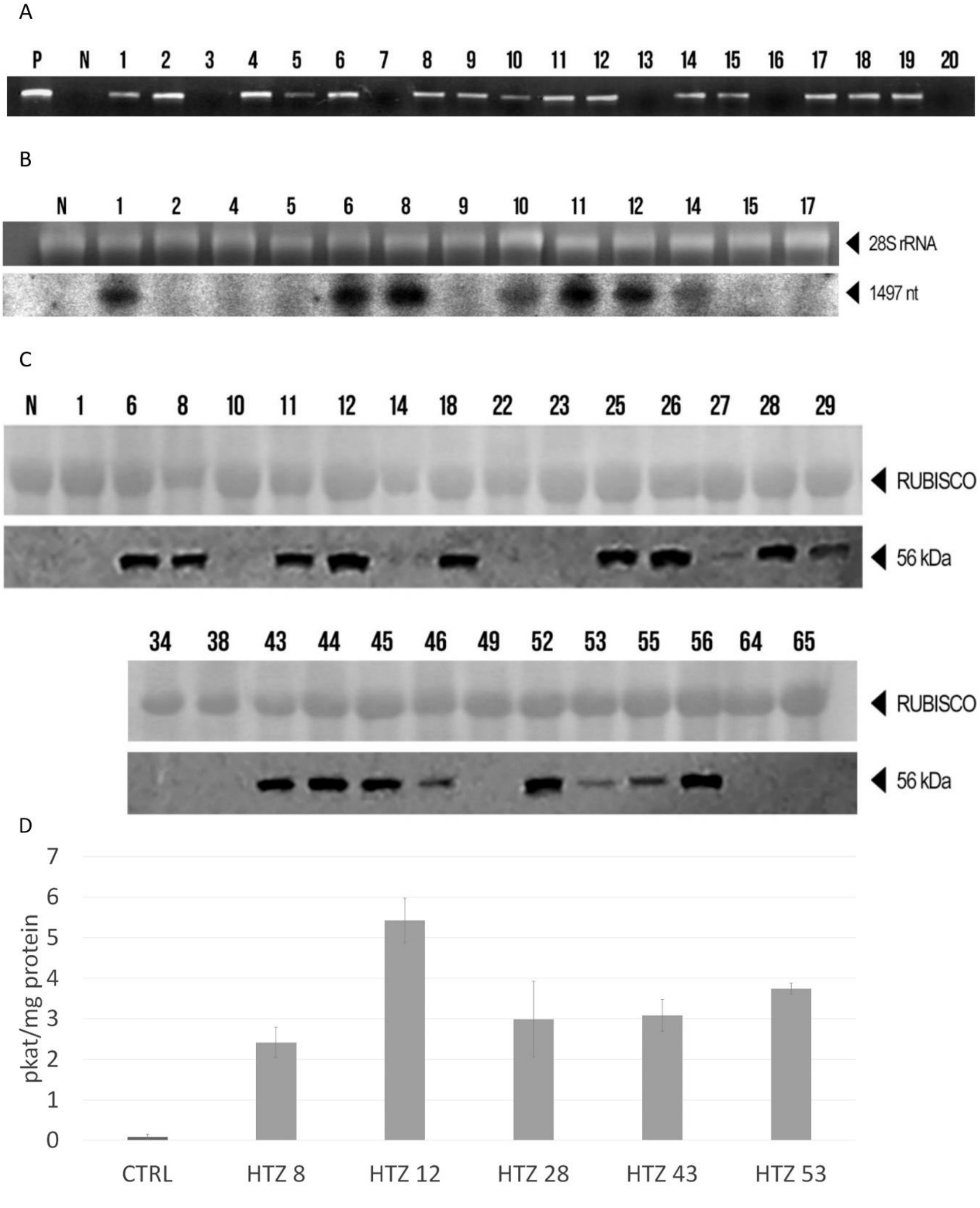

Supplementary File S3. Consecutive selection steps of potato plants transformed with TH gene from *R. norvegicus*. A – sample PCR product electrophoresis separation with use of primers specific to a 486-nt fragment of TH cDNA from R. norvegicus. P – positive control, N – negative control (non-transgenic potato); B – sample Northern blot of RNA samples from transgenic potato plants bearing TH gene from R. norvegicus. P – positive control, N – negative control (wt potato); C – Western blot analysis of proteins isolated from transgenic HTZ potato plants. N – negative control (non-transgenic potato); activity of the TH protein in extracts from the HTZ transgenic potato lines (HTZ 8, HTZ 12, HTZ 28, HTZ 43, HTZ 53) relatively to control (CTRL) expressed in pkatals calculated from the amount of L-DOPA produced from tyrosine.

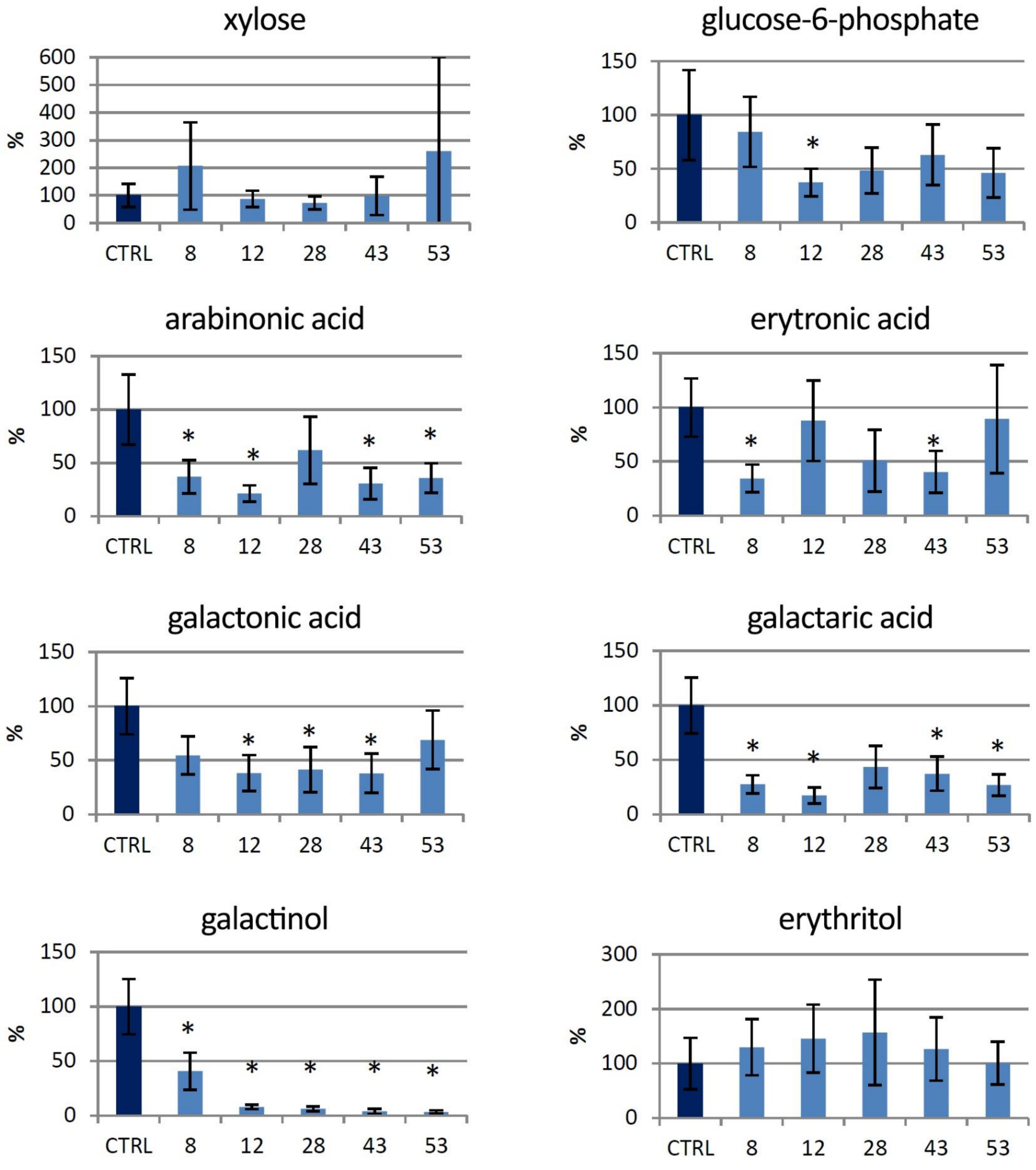

Supplementary File S4. Levels of soluble sugars in HTZ transgenic lines relatively to the control (non-transgenic potato) obtained with GC-MS technique presented as means of 6 biological replicates ± standard deviation. Statistically significant changes (p<0.05) are marked with asterisks.

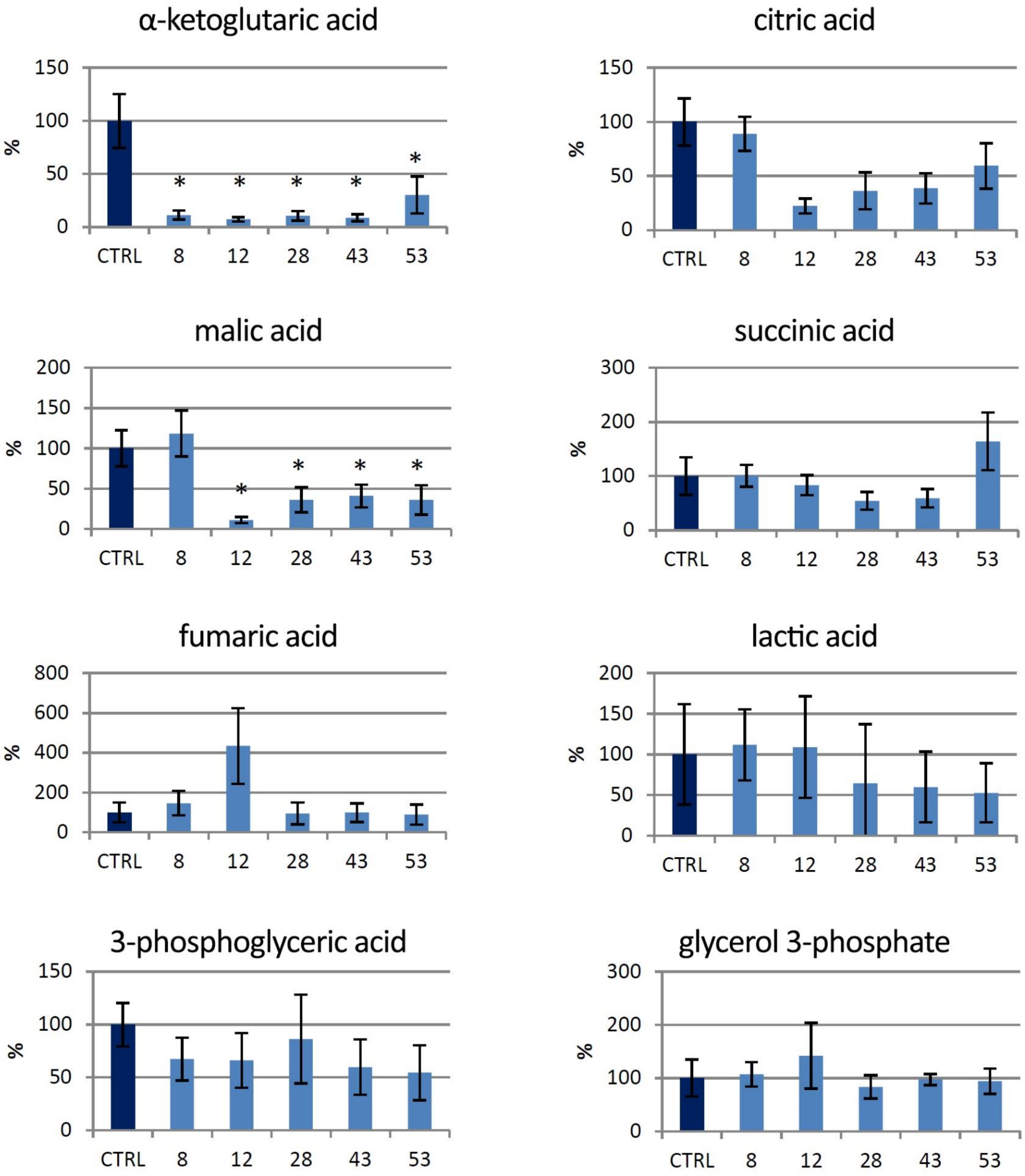

Supplementary File S5. Levels of organic acids in HTZ transgenic lines relatively to the control (non-transgenic potato) obtained with GC-MS technique presented as means of 6 biological replicates ± standard deviation. Statistically significant changes ($p<0.05$) are marked with asterisks.

AMINO ACID	CTRL	HTZ8	HTZ12	HTZ28	HTZ43	HTZ53
GLYCINE	100% ± 12.8	39.6% ± 8.3	145.3% ± 14.2	107.6% ± 19.2	73.4% ± 11.2	115.9% ± 24.8
LEUCINE	100% ± 53.1	56.4% ± 21.7	75.4% ± 29.7	77.9% ± 42.4	60.6% ± 29.3	72.7% ± 36.9
ISOLEUCINE	100% ± 44.6	33.7% ± 11.8	35.4% ± 12.6	51.8 ± 26.4	35.1 ± 16.2	41.3 ± 19.6
VALINE	100% ± 55.1	56.2% ± 22.2	61.9% ± 22.5	84.1% ± 40.0	67.0% ± 31.7	88.2% ± 45.4
PROLINE	100% ± 47.1	38.6% ± 13.8	12.0% ± 4.5	55.0% ± 29.0	18.6% ± 8.0	19.8% ± 9.6
SERINE	100% ± 45.9	60.8% ± 20.9	79.2% ± 36.0	78.4% ± 55.6	61.4% ± 28.0	78.0% ± 57.4
THREONINE	100% ± 46.4	122.9% ± 34.2	63.4% ± 39.7	142.4% ± 93.2	100.5% ± 33.1	72.9% ± 56.5
ASPARAGINE	100% ± 30.1	59.8% ± 20.4	24.4% ± 14.3	50.2% ± 26.9	69.1% ± 32.4	34.7% ± 17.1
ASPARTIC ACID	100% ± 63.7	113.5% ± 118.4	120.2% ± 37.9	126.5% ± 75.2	125.5% ± 91.2	182.6% ± 109.3
GLUTAMINE	100% ± 52.9	128.3% ± 109.4	74.8% ± 49.9	200.7% ±129.5	165.7% ± 98.8	125.1% ±83.3
GLUTAMIC ACID	100% ± 32.8	98.7% ± 26.3	47.7% ± 16.4	71.0% ± 34.3	70.0% ± 20.4	56.7% ± 34.6
LYSINE	100% ± 17.6	38.3% ± 11.5	61.8% ± 17.9	86.9% ± 30.6	74.2% ± 27.7	60.0% ± 22.1
ARGININE	100% ± 31.3	24.0% ± 26.9	314.3% ± 123.9	120.7% ± 63.5	60.0% ± 44.3	79.9% ± 55.4
METIONINE	100% ± 43.7	164.5% ± 78.7	49.0% ± 22.0	102.6% ±83.6	99.4% ± 68.5	81.0% ± 53.5
HISTIDINE	100% ± 22.8	45.1% ± 13.1	91.7% ± 32.8	119.3% ± 47.9	61.3% ± 31.7	54.8% ± 16.3
TRYPTOPHAN	100% ± 17.0	22.8% ± 7.9	77.1% ± 27.8	38.4% ± 15.5	26.7% ± 12.2	37.9% ± 11.9
TYROSINE	100% ± 14.5	20.1% ± 6.4	43.6% ± 12.9	39.0% ± 17.1	23.2% ± 10.6	41.4% ± 16.0
ORNITHINE	100% ± 43.7	198.5% ± 22.2	778.2% ± 201.4	83.7% ± 38.5	114.4% ± 18.1	181.8% ± 168.5
PUTRESCINE	100% ± 28.3	144.2% ± 42.8	168.9% ± 71.0	53.5% ± 27.2	106.0% ± 51.2	88.8% ± 52.8
OCTOPAMINE	100% ± 38.8	146.0% ± 10.3	140.1% ± 15.5	81.6% ± 31.3	84.7% ± 12.4	82.7% ± 8.2

Supplementary File S6. Levels of soluble amino acids in HTZ transgenic lines relatively to the control (non-transgenic potato) obtained with GC-MS technique presented as means of 6 biological replicates ± standard deviation. Statistically significant changes ($p<0.05$) are marked with red color.

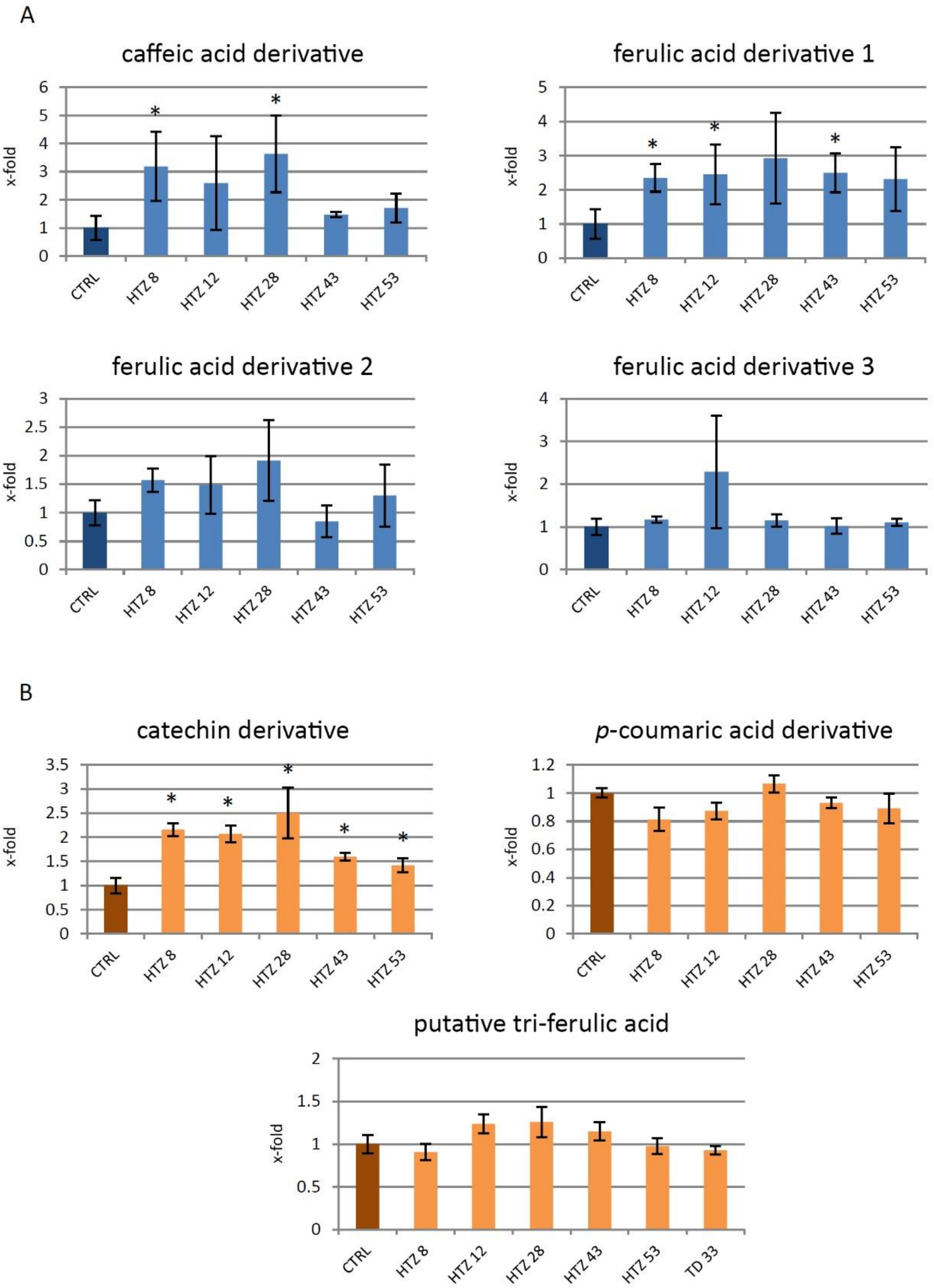

Supplementary File S7. Levels of soluble (A) and cell wall bound (B) phenolics' derivatives in HTZ transgenic lines relatively to the control (non-transgenic potato) obtained with LC-MS technique presented as means of 6 biological replicates ± standard deviation. Statistically significant changes ($p<0.05$) are marked with red color.

qseqid	sseqid	pident	length	mismatch	gapopen	qstart	qend	sstart	send	evalue	bitscore	stitle
NP_036872.1	tr|A0A1D1XK09|A0A1D1XK09_9ARAE	52,607	422	184	4	85	497	161	575	8,81E-154	456	Tyrosine 3-monooxygenase OS=Anthurium amnicola OX=1678845 GN=ple PE=4 SV=1
NP_036872.1	tr|E5KBU3|E5KBU3_PINTA	47,931	290	146	2	164	453	67	351	1,07E-91	289	Chloroplast phenylalanine hydroxylase OS=Pinus taeda OX=3352 PE=2 SV=1
NP_036872.1	tr|A0A5J4XP48|A0A5J4XP48_9CHLO	48,789	289	142	2	167	455	63	345	2,49E-90	286	Tryptophan 5-hydroxylase 1 OS=Trebouxia sp. A1-2 OX=2608996 GN=FRX49_09034 PE=4 SV=1
NP_036872.1	tr|A0A383VAB6|A0A383VAB6_TETOB	47,735	287	144	2	167	453	52	332	1,16E-89	283	BH4_AAA_HYDROXYL_2 domain-containing protein OS=Tetradesmus obliquus OX=3088 GN=BQ4739_LOCUS2200 PE=4 SV=1
NP_036872.1	tr|I0YV42|I0YV42_COCSC	50,909	275	131	1	181	455	10	280	1,77E-88	279	Aromatic amino acid hydroxylase (Fragment) OS=Coccomyxa subellipsoidea (strain C-169) OX=574566 GN=COCSUDRAFT_16625 PE=4 SV=1
NP_036872.1	tr|E5KBU4|E5KBU4_PHYPA	46,44	323	160	5	146	460	70	387	1,48E-87	280	Chloroplast phenylalanine hydroxylase OS=Physcomitrella patens subsp. patens OX=3218 GN=PHYPA_011502 PE=2 SV=1
NP_036872.1	tr|A9T8N9|A9T8N9_PHYPA	50,362	276	129	2	184	455	1	272	3,73E-86	272	Predicted protein (Fragment) OS=Physcomitrella patens subsp. patens OX=3218 GN=PHYPADRAFT_5250 PE=4 SV=1
NP_036872.1	tr|A0A1Y1IAC0|A0A1Y1IAC0_KLENI	46,233	292	148	4	167	454	74	360	7,84E-86	275	Aromatic amino acid hydroxylase OS=Klebsormidium nitens OX=105231 GN=KFL_003840020 PE=4 SV=1
NP_036872.1	tr|A0A087SFZ0|A0A087SFZ0_AUXPR	45,361	291	153	2	167	457	32	316	3,41E-82	264	Tryptophan 5-hydroxylase 1 OS=Auxenochlorella protothecoides OX=3075 GN=APUTEX25_005193 PE=4 SV=1
NP_036872.1	tr|A0A1D1ZM34|A0A1D1ZM34_AUXPR	45,361	291	153	2	167	457	84	368	1,13E-81	264	BH4_AAA_HYDROXYL_2 domain-containing protein (Fragment) OS=Auxenochlorella protothecoides OX=3075 GN=g.17877 PE=4 SV=1
NP_036872.1	tr|A0A2P6TZP0|A0A2P6TZP0_CHLSO	46,048	291	145	5	167	454	72	353	1,88E-80	261	Phenylalanine hydroxylase OS=Chlorella sorokiniana OX=3076 GN=C2E21_1449 PE=4 SV=1
NP_036872.1	tr|A0A5B8MVV0|A0A5B8MVV0_9CHLO	44,702	302	155	5	151	449	58	350	2,27E-80	260	Aromatic amino acid hydroxylase OS=Chloropicon primus OX=1764295 GN=A3770_13p68940 PE=4 SV=1
NP_036872.1	tr|A0A2P6V2S1|A0A2P6V2S1_9CHLO	45,361	291	143	5	168	453	75	354	2,50E-80	260	Chloroplast phenylalanine hydroxylase OS=Micractinium conductrix OX=554055 GN=C2E20_8090 PE=4 SV=1
NP_036872.1	tr|A0A2P6VBN6|A0A2P6VBN6_9CHLO	43,533	317	158	6	142	453	32	332	2,78E-80	260	Chloroplast phenylalanine hydroxylase OS=Micractinium conductrix OX=554055 GN=C2E20_5121 PE=4 SV=1
NP_036872.1	tr|A8HQD7|A8HQD7_CHLRE	45,907	281	148	1	181	461	81	357	3,54E-80	260	Aromatic amino acid hydroxylase-related protein OS=Chlamydomonas reinhardtii OX=3055 GN=AAH1 PE=2 SV=1
NP_036872.1	tr|D8U9W1|D8U9W1_VOLCA	45,804	286	147	2	181	462	11	292	3,14E-79	255	BH4_AAA_HYDROXYL_2 domain-containing protein OS=Volvox carteri f. nagariensis OX=3068 GN=VOLCADRAFT_96350 PE=4 SV=1
NP_036872.1	tr|A0A061R0L5|A0A061R0L5_9CHLO	45,517	290	146	5	168	454	64	344	1,85E-78	255	Tryptophan 5-monooxygenase OS=Tetraselmis sp. GSL018 OX=582737 GN=TPH PE=4 SV=1
NP_036872.1	tr|A0A250WYI1|A0A250WYI1_9CHLO	44,097	288	155	2	168	455	83	364	4,32E-78	255	BH4_AAA_HYDROXYL_2 domain-containing protein OS=Chlamydomonas eustigma OX=1157962 GN=CEUSTIGMA_g3346.t1 PE=4 SV=1
NP_036872.1	tr|E1ZIW4|E1ZIW4_CHLVA	51,316	228	107	1	187	414	2	225	6,65E-77	248	BH4_AAA_HYDROXYL_2 domain-containing protein (Fragment) OS=Chlorella variabilis OX=554065 GN=CHLNCDRAFT_24756 PE=4 SV=1
NP_036872.1	tr|A0A1Y1I6U4|A0A1Y1I6U4_KLENI	42,466	292	153	4	174	454	149	436	1,01E-76	253	Aromatic amino acid hydroxylase OS=Klebsormidium nitens OX=105231 GN=KFL_001890200 PE=4 SV=1
NP_036872.1	tr|A0A2J8AD84|A0A2J8AD84_9CHLO	43,003	293	161	2	169	461	42	328	2,64E-76	249	Tryptophan 5-hydroxylase 1 OS=Tetrabaena socialis OX=47790 GN=TSOC_002790 PE=4 SV=1
NP_036872.1	tr|A0A0D2MT76|A0A0D2MT76_9CHLO	48,79	248	117	2	168	415	50	287	5,44E-76	247	Phenylalanine-4-hydroxylase OS=Monoraphidium neglectum OX=145388 GN=MNEG_4320 PE=4 SV=1
NP_036872.1	tr|A0A150G664|A0A150G664_GONPE	46,094	256	134	1	206	461	1	252	1,03E-69	229	BH4_AAA_HYDROXYL_2 domain-containing protein OS=Gonium pectorale OX=33097 GN=GPECTOR_56g434 PE=4 SV=1
NP_036872.1	tr|A0A2V0PJD4|A0A2V0PJD4_9CHLO	44,014	284	135	6	168	448	279	541	1,54E-62	220	Chloroplast phenylalanine hydroxylase OS=Raphidocelis subcapitata OX=307507 GN=Rsub_10342 PE=4 SV=1
NP_036872.1	tr|A0A061RS39|A0A061RS39_9CHLO	49,65	143	66	3	315	454	1	140	3,92E-36	137	Tryptophan 5-monooxygenase OS=Tetraselmis sp. GSL018 OX=582737 GN=TPH PE=4 SV=1
NP_036872.1	tr|A0A2P6V6F8|A0A2P6V6F8_9CHLO	43,548	124	60	3	335	453	11	129	6,96E-23	101	Chloroplast phenylalanine hydroxylase OS=Micractinium conductrix OX=554055 GN=C2E20_6935 PE=4 SV=1
NP_036872.1	tr|A0A6A0ACI4|A0A6A0ACI4_HAELA	47,297	74	33	3	364	434	40	110	1,02E-09	62,8	Tryptophan 5-monooxygenase (Fragment) OS=Haematococcus lacustris OX=44745 GN=HaLaN_28756 PE=4 SV=1
NP_036872.1	tr|A0A6A0ACI4|A0A6A0ACI4_HAELA	47,059	34	18	0	263	296	11	44	0,006	43,5	Tryptophan 5-monooxygenase (Fragment) OS=Haematococcus lacustris OX=44745 GN=HaLaN_28756 PE=4 SV=1
NP_036872.1	tr|A0A4Y7KYN9|A0A4Y7KYN9_PAPSO	27,068	133	71	7	68	182	11	135	0,2	42	Uncharacterized protein OS=Papaver somniferum OX=3469 GN=C5167_001434 PE=3 SV=1
NP_036872.1	tr|A0A445L060|A0A445L060_GLYSO	46,154	26	13	1	308	333	39	63	7,3	34,7	AAI domain-containing protein (Fragment) OS=Glycine soja OX=3848 GN=D0Y65_009689 PE=4 SV=1
NP_036872.1	tr|I1JWI3|I1JWI3_SOYBN	46,154	26	13	1	308	333	39	63	7,7	34,7	AAI domain-containing protein OS=Glycine max OX=3847 GN=100817165 PE=4 SV=1

NP_036872.1	tr|A0A0B2QQV3|A0A0B2QQV3_GLYSO	46,154	26	13	1	308	333	29	53	7,8	34,7	AAI domain-containing protein OS=Glycine soja OX=3848 GN=glysoja_047386 PE=4 SV=1
NP_036872.1	tr|G7L2F2|G7L2F2_MEDTR	35,484	62	40	0	426	487	168	229	8,2	36,6	DUF295 family protein OS=Medicago truncatula OX=3880 GN=MTR_7g076100 PE=4 SV=1
NP_036872.1	tr|A2Q5C0|A2Q5C0_MEDTR	35,484	62	40	0	426	487	176	237	9,3	36,2	DUF295 domain-containing protein OS=Medicago truncatula OX=3880 GN=MtrDRAFT_AC160924g10v1 PE=4 SV=1

Supplementary File S8. Similarity analysis (Blastp) of rat tyrosine hydroxylase against plant protein sequences (Swiss Prot Database).

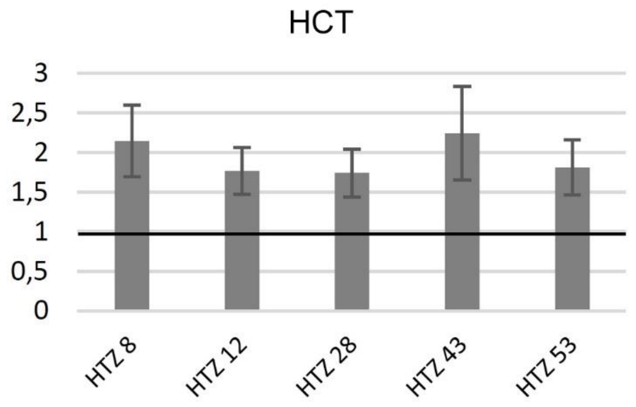

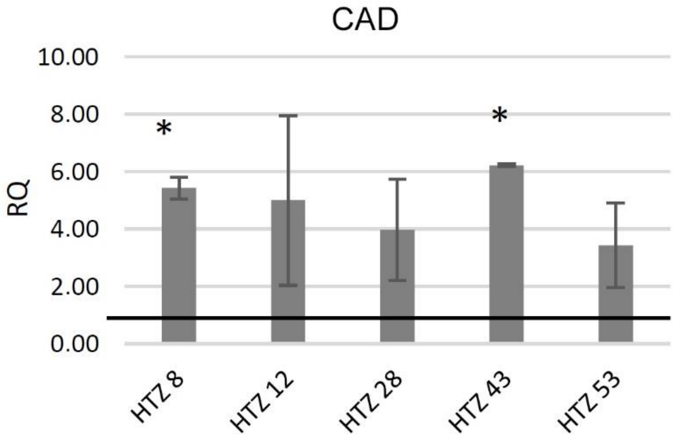

Supplementary File S9. The levels of transcripts of cinnamyl-alcohol dehydrogenase (CAD) and shikimate/quinate hydroxy-cinnamoyl transferase (HCT) genes in the HTZ lines presented as relative quantification (RQ) in relation to the control (horizontal line at RQ = 1). Elongation factor gene was used as a reference gene. The results were obtained with RT-qPCR method on cDNA matrix as mean values of 3 biological repeats ± standard deviation. Statistically significant changes ($p<0.05$) are marked with asterisks.
