# Supplementary material for: Expression of the Tyrosine Hydroxylase Gene from Rat Leads to Oxidative Stress in Potato Plants
Source: Antioxidants (Basel). 2020 Aug 7;9(8):717. doi: 10.3390/antiox9080717 (PMC7465045; doi:10.3390/antiox9080717)
Supplement: Supplementary file 1 [file antioxidants-09-00717-s001.zip › Supple/SupplementaryFileS8B.pdf]

```
# hmmsearch :: search profile(s) against a sequence database
# HMMER 3.2.1 (June 2018); http://hmmer.org/
# Copyright (C) 2018 Howard Hughes Medical Institute.
# Freely distributed under the BSD open source license.
# - - - - - - - - - - - - - - - - - - - - - - - - - - - - - - - - - - - - -
# query HMM file:                  seq.hmm
# target sequence database:        db/plant-only-full/uniprot-reviewed_no+taxonomy_viridiplantae.fasta
# - - - - - - - - - - - - - - - - - - - - - - - - - - - - - - - - - - - - -

Query:       sequence  [M=498]
Scores for complete sequences (score includes all domains):
   --- full sequence ---   --- best 1 domain ---    -#dom-
    E-value  score  bias    E-value  score  bias    exp  N  Sequence                        Description
    ------- ------ -----    ------- ------ -----   ---- --  --------                        -----------
   2.5e-149  507.1   0.1   4.2e-149  506.3   0.0    1.3  1  tr|A0A1D1XK09|A0A1D1XK09_9ARAE  Tyrosine 3-monooxygenase OS=Anth
    1.2e-87  303.6   0.0    1.5e-87  303.3   0.0    1.0  1  tr|E5KBU3|E5KBU3_PINTA          Chloroplast phenylalanine hydrox
      1e-86  300.6   0.0    1.4e-86  300.1   0.0    1.2  1  tr|A0A5J4XP48|A0A5J4XP48_9CHLO  Tryptophan 5-hydroxylase 1 OS=Tr
      1e-86  300.5   0.0    1.2e-86  300.2   0.0    1.0  1  tr|I0YV42|I0YV42_COCSC          Aromatic amino acid hydroxylase
    5.3e-85  294.9   0.0    7.4e-85  294.4   0.0    1.1  1  tr|E5KBU4|E5KBU4_PHYPA          Chloroplast phenylalanine hydrox
    8.5e-85  294.2   0.0    9.8e-85  294.0   0.0    1.0  1  tr|A0A383VAB6|A0A383VAB6_TETOB  BH4_AAA_HYDROXYL_2 domain-contai
    1.7e-82  286.6   0.0    1.9e-82  286.5   0.0    1.0  1  tr|A9T8N9|A9T8N9_PHYPA          Predicted protein (Fragment) OS=
    1.7e-81  283.3   0.0    2.2e-81  282.9   0.0    1.0  1  tr|A0A1Y1IAC0|A0A1Y1IAC0_KLENI  Aromatic amino acid hydroxylase
    1.8e-78  273.3   0.0    2.4e-78  272.9   0.0    1.0  1  tr|A0A2P6TZP0|A0A2P6TZP0_CHLSO  Phenylalanine hydroxylase OS=Chl
    4.6e-78  272.0   0.0    5.8e-78  271.7   0.0    1.0  1  tr|A0A087SFZ0|A0A087SFZ0_AUXPR  Tryptophan 5-hydroxylase 1 OS=Au
    9.3e-78  271.0   0.0    1.3e-77  270.5   0.0    1.0  1  tr|A0A1D1ZM34|A0A1D1ZM34_AUXPR  BH4_AAA_HYDROXYL_2 domain-contai
    9.9e-78  270.9   0.0    1.3e-77  270.5   0.0    1.0  1  tr|A0A5B8MVV0|A0A5B8MVV0_9CHLO  Aromatic amino acid hydroxylase
    4.1e-77  268.8   0.0    5.1e-77  268.5   0.0    1.0  1  tr|A0A2P6VBN6|A0A2P6VBN6_9CHLO  Chloroplast phenylalanine hydrox
    9.4e-77  267.7   0.0    1.5e-76  267.0   0.0    1.2  1  tr|A0A2P6V2S1|A0A2P6V2S1_9CHLO  Chloroplast phenylalanine hydrox
    5.5e-76  265.1   0.0    6.9e-76  264.8   0.0    1.0  1  tr|A8HQD7|A8HQD7_CHLRE          Aromatic amino acid hydroxylase-
      7e-76  264.8   0.0    8.6e-76  264.5   0.0    1.0  1  tr|A0A061R0L5|A0A061R0L5_9CHLO  Tryptophan 5-monooxygenase OS=Te
    5.3e-75  261.9   0.0    6.4e-75  261.6   0.0    1.0  1  tr|D8U9W1|D8U9W1_VOLCA          BH4_AAA_HYDROXYL_2 domain-contai
      2e-73  256.7   0.0    2.8e-73  256.2   0.0    1.1  1  tr|A0A250WYI1|A0A250WYI1_9CHLO  BH4_AAA_HYDROXYL_2 domain-contai
    2.4e-73  256.4   0.0    2.9e-73  256.2   0.0    1.0  1  tr|E1ZIW4|E1ZIW4_CHLVA          BH4_AAA_HYDROXYL_2 domain-contai
    4.3e-72  252.3   0.0    5.4e-72  252.0   0.0    1.1  1  tr|A0A1Y1I6U4|A0A1Y1I6U4_KLENI  Aromatic amino acid hydroxylase
    7.7e-72  251.4   0.0    9.7e-72  251.1   0.0    1.1  1  tr|A0A2J8AD84|A0A2J8AD84_9CHLO  Tryptophan 5-hydroxylase 1 OS=Te
    5.4e-71  248.7   0.0    6.7e-71  248.4   0.0    1.1  1  tr|A0A0D2MT76|A0A0D2MT76_9CHLO  Phenylalanine-4-hydroxylase OS=M
    5.9e-67  235.3   0.0    6.9e-67  235.1   0.0    1.0  1  tr|A0A150G664|A0A150G664_GONPE  BH4_AAA_HYDROXYL_2 domain-contai
    5.5e-60  212.3   2.2    1.3e-59  211.2   0.0    2.5  2  tr|A0A2V0PJD4|A0A2V0PJD4_9CHLO  Chloroplast phenylalanine hydrox
    9.5e-37  135.7   0.0    1.1e-36  135.5   0.0    1.0  1  tr|A0A061RS39|A0A061RS39_9CHLO  Tryptophan 5-monooxygenase OS=Te
    6.1e-24   93.5   0.0    7.6e-24   93.1   0.0    1.0  1  tr|A0A2P6V6F8|A0A2P6V6F8_9CHLO  Chloroplast phenylalanine hydrox
      4e-17   71.0   0.2    4.6e-10   47.7   0.1    2.0  2  tr|A0A6A0ACI4|A0A6A0ACI4_HAELA  Tryptophan 5-monooxygenase (Frag
  ------ inclusion threshold ------
       0.22   19.1   0.1       0.29   18.6   0.1    1.1  1  tr|A0A396H0S2|A0A396H0S2_MEDTR  DUF295 domain-containing protein
       0.26   18.8   0.1       0.35   18.4   0.1    1.1  1  tr|G7L2F2|G7L2F2_MEDTR          DUF295 family protein OS=Medicag
       0.27   18.7   0.1       0.37   18.3   0.1    1.1  1  tr|A2Q5C0|A2Q5C0_MEDTR          DUF295 domain-containing protein
       0.58   17.6   0.2       0.58   17.6   0.2    1.8  2  tr|A0A199VCK3|A0A199VCK3_ANACO  AP2/ERF and B3 domain-containing
```

0.84	17.1	0.0	6.5e+02	7.6	0.0	2.1	2	tr|A0A6A5NPF4|A0A6A5NPF4_LUPAL	Uncharacterized protein OS=Lupin
1.2	16.7	0.6	1.6	16.2	0.6	1.1	1	tr|A0A061SG95|A0A061SG95_9CHLO	Uncharacterized protein (Fragmen
1.3	16.5	0.4	1.5	16.3	0.4	1.0	1	tr|A0A397Y5H5|A0A397Y5H5_BRACM	Uncharacterized protein (Fragmen
1.6	16.2	0.0	7.1e+02	7.5	0.0	2.1	2	tr|A0A6A4PI42|A0A6A4PI42_LUPAL	Putative ribosomal protein S1 OS
3.9	14.9	5.2	5.1	14.5	5.2	1.1	1	tr|A0A2Z6ZTF1|A0A2Z6ZTF1_9LAMI	Uncharacterized protein OS=Dorco
4.1	14.9	0.9	6.6	14.2	0.9	1.2	1	tr|I0YL19|I0YL19_COCSC	Uncharacterized protein OS=Cocco
4.2	14.8	0.2	5.4	14.4	0.2	1.1	1	tr|A0A059CY94|A0A059CY94_EUCGR	Uncharacterized protein OS=Eucal
4.5	14.7	0.6	6	14.3	0.6	1.1	1	tr|A0A5D2SK86|A0A5D2SK86_GOSMU	Uncharacterized protein OS=Gossy
5	14.6	0.2	6.5	14.2	0.2	1.1	1	tr|A0A059CZ03|A0A059CZ03_EUCGR	Uncharacterized protein OS=Eucal
5.6	14.4	1.0	9.1	13.7	1.0	1.3	1	tr|B9RTY8|B9RTY8_RICCO	Protein binding protein, putativ
5.7	14.4	0.1	9.1	13.7	0.1	1.3	1	tr|I0YKX2|I0YKX2_COCSC	BZIP domain-containing protein O
6.1	14.3	0.1	7.2	14.0	0.1	1.1	1	tr|A0A2P5EQI7|A0A2P5EQI7_TREOI	Uncharacterized protein OS=Trema
6.1	14.3	0.7	6.3	14.2	0.7	1.1	1	tr|A0A2P6TE94|A0A2P6TE94_CHLSO	SufE chloroplastic OS=Chlorella
7.5	14.0	1.2	9.6	13.6	1.2	1.1	1	tr|A0A699ZJV5|A0A699ZJV5_HAELA	TAF domain-containing protein OS
7.7	13.9	0.9	16	12.9	0.9	1.4	1	tr|I0ZAJ8|I0ZAJ8_COCSC	Uncharacterized protein OS=Cocco
7.8	13.9	1.5	11	13.4	1.5	1.2	1	tr|A0A2I0AQ32|A0A2I0AQ32_9ASPA	40S ribosomal protein S4 OS=Apos
7.9	13.9	0.4	10	13.5	0.4	1.1	1	tr|A0A5D2IGR4|A0A5D2IGR4_GOSTO	Uncharacterized protein OS=Gossy
8	13.9	1.0	11	13.4	1.0	1.1	1	tr|A0A2R6X400|A0A2R6X400_MARPO	Uncharacterized protein OS=March
8.1	13.9	0.4	10	13.5	0.4	1.1	1	tr|A0A0B0NP41|A0A0B0NP41_GOSAR	39S ribosomal L47, mitochondrial
8.2	13.9	0.4	10	13.5	0.4	1.1	1	tr|A0A1U8MSW2|A0A1U8MSW2_GOSHI	39S ribosomal protein L47, mitoc
8.2	13.9	0.4	10	13.5	0.4	1.1	1	tr|A0A0D2T8Q2|A0A0D2T8Q2_GOSRA	Uncharacterized protein OS=Gossy
8.2	13.9	0.4	10	13.5	0.4	1.1	1	tr|A0A1U8MRB8|A0A1U8MRB8_GOSHI	39S ribosomal protein L47, mitoc
8.2	13.9	0.4	10	13.5	0.4	1.1	1	tr|A0A5D2EGM7|A0A5D2EGM7_GOSDA	Uncharacterized protein OS=Gossy
8.2	13.9	0.4	10	13.5	0.4	1.1	1	tr|A0A5D2N494|A0A5D2N494_GOSTO	Uncharacterized protein OS=Gossy
8.2	13.9	0.4	10	13.5	0.4	1.1	1	tr|A0A5D2X3A8|A0A5D2X3A8_GOSMU	Uncharacterized protein OS=Gossy
8.2	13.9	0.4	10	13.5	0.4	1.1	1	tr|A0A5J5TKL3|A0A5J5TKL3_GOSBA	Uncharacterized protein OS=Gossy
8.2	13.8	1.0	13	13.2	1.0	1.2	1	tr|A0A0D2MIV3|A0A0D2MIV3_9CHLO	NFACT-R_1 domain-containing prot
8.4	13.8	0.6	12	13.3	0.6	1.2	1	tr|A0A1D6FM46|A0A1D6FM46_MAIZE	Protein apaG OS=Zea mays OX=4577
8.4	13.8	0.6	12	13.3	0.6	1.2	1	tr|A0A3L6DRF5|A0A3L6DRF5_MAIZE	Protein ApaG OS=Zea mays OX=4577
8.5	13.8	2.9	9.5	13.6	2.9	1.0	1	tr|A0A2Z6ZTG8|A0A2Z6ZTG8_9LAMI	Uncharacterized protein OS=Dorco
8.8	13.7	0.2	9.2	13.7	0.2	1.1	1	tr|I1JWI3|I1JWI3_SOYBN	AAI domain-containing protein OS
9.1	13.7	0.0	14	13.1	0.0	1.2	1	tr|A0A0E0JL63|A0A0E0JL63_ORYPU	Uncharacterized protein OS=Oryza
9.6	13.6	0.0	13	13.2	0.0	1.1	1	tr|A0A446NN00|A0A446NN00_TRITD	Uncharacterized protein OS=Triti
9.7	13.6	0.3	11	13.4	0.3	1.1	1	tr|A0A445L060|A0A445L060_GLYSO	AAI domain-containing protein (F
9.8	13.6	0.0	15	13.0	0.0	1.2	1	tr|J3L1W2|J3L1W2_ORYBR	Uncharacterized protein OS=Oryza

Domain annotation for each sequence (and alignments):

>> tr|A0A1D1XK09|A0A1D1XK09_9ARAE Tyrosine 3-monooxygenase OS=Anthurium amnicola OX=1678845 GN=ple PE=4 SV=1

#		score	bias	c-Evalue	i-Evalue	hmmfrom	hmm to		alifrom	ali to		envfrom	env to		acc
1	!	506.3	0.0	2.7e-154	4.2e-149	75	495	..	148	573	..	134	576	..	0.92

Alignments for each domain:

== domain 1 score: 506.3 bits; conditional E-value: 2.7e-154

```
                  sequence  75 eerdgn.avln..llfslrgtkpsslsravkvfetfeakihhletrpaqrplagsphleyfvrfevpsgdlaallssvr 150
```

```
                                e++d + av    l++ lr     slsr +k +e ++  + +le+rp++ p     +l+ +v +++    l  ll s+r
  tr|A0A1D1XK09|A0A1D1XK09_9ARAE 148 ENKDAEtAVQRaaLVLRLRE-SGGSLSRILKTVENLKGTVVQLESRPSKTPG---CQLDILVTLDMARSSLLQLLRSLR 222
                                55555414443114444554.3579************************994...68********************** PP

                    sequence 151 rvsd..dvrsaredkv....pwfprkvseldkchhlvtkfdpdldldhpgfsdqvyrqrrkliaeiafqykhgepiphv 223
                                +      v    e+kv     wfpr+  +ld+c hl+tk++p+ld++hpgf+d+ yr+rrk ia+iaf yk g+piph+
  tr|A0A1D1XK09|A0A1D1XK09_9ARAE 223 QSGAlaGVTLLSENKVdakeAWFPRHARDLDNCNHLMTKYEPELDMNHPGFADKDYRERRKHIANIAFSYKFGDPIPHI 301
                                976422566667777643337********************************************************** PP

                    sequence 224 eytaeeiatwkevyvtlkglyathacrehlegfqllerycgyredsipqledvsrflkertgfqlrpvagllsardfla 302
                                +y  +ei+tw  v+ t+  l   hac e+   fqll+    +re +ipql+d+s fl+ +tgf lrp agll+ardfla
  tr|A0A1D1XK09|A0A1D1XK09_9ARAE 302 DYNDDEISTWTSVFRTVVDLLPKHACAEYNRVFQLLQDEGIFREGAIPQLKDMSDFLQRHTGFTLRPAAGLLTARDFLA 380
                                ******************************************************************************* PP

                    sequence 303 slafrvfqctqyirhasspmhspepdcchellghvpmladrtfaqfsqdiglaslgasdeeieklstvywftvefglck 381
                                slafrvfq tqy+rh +sp+h+pepdc hellgh+p+lad +faqfsq+iglaslgasd eieklstvywftvefglck
  tr|A0A1D1XK09|A0A1D1XK09_9ARAE 381 SLAFRVFQSTQYVRHPTSPFHTPEPDCIHELLGHMPLLADPSFAQFSQEIGLASLGASDAEIEKLSTVYWFTVEFGLCK 459
                                ******************************************************************************* PP

                    sequence 382 qngelkaygagllssygellhslseepevrafdpdtaavqpyqdqtyqpvyfvsesfndakdklrnyasriqrpfsvkf 460
                                + g +kaygaglls+ygellh++s++pe r f+p ++a+qpyqdq yqp+y+v+esf+dak+k+r++ + + rp+ v+f
  tr|A0A1D1XK09|A0A1D1XK09_9ARAE 460 EGGAVKAYGAGLLSAYGELLHAVSDKPERRPFEPASTALQPYQDQEYQPIYYVAESFEDAKEKFRKWVAGMSRPYEVRF 538
                                ******************************************************************************* PP

                    sequence 461 dpytlaidvldsphtiqrslegvqdelhtlahals 495
                                +p+t  ++vlds   ++  +  ++ e+  l  a+
  tr|A0A1D1XK09|A0A1D1XK09_9ARAE 539 NPHTQRVEVLDSVSGLENLMAQLHLEMMHLQNAVN 573
                                ************99988888777777777776665 PP

>> tr|E5KBU3|E5KBU3_PINTA  Chloroplast phenylalanine hydroxylase OS=Pinus taeda OX=3352 PE=2 SV=1
   #    score  bias  c-Evalue  i-Evalue hmmfrom  hmm to    alifrom  ali to    envfrom  env to     acc
 ---   ------ ----- --------- --------- ------- -------    ------- -------    ------- -------    ----
   1 !  303.3   0.0   9.5e-93   1.5e-87     162     453 ..      65     351 ..      49     353 .. 0.96

  Alignments for each domain:
  == domain 1  score: 303.3 bits;  conditional E-value: 9.5e-93
                sequence 162 dkvpwfprkvseldkchhlvtkfdpdldldhpgfsdqvyrqrrkliaeiafqykhgepiphveytaeeiatwkevyvtlkglyatha 248
                               +p +pr + ++ +  h++  f  +l  dhpg+ d+ y++rr  ia++a ++k gepip ++yt+ee   w ev   l  ly +ha
  tr|E5KBU3|E5KBU3_PINTA  65 AVLPPVPRSIHDIPNGDHIL-GFGANLAEDHPGYHDEEYKRRRSCIADLAKKHKIGEPIPEINYTTEEAHVWAEVLTKLSELYPSHA 150
                             557899*********99986.6***************************************************************** PP

                sequence 249 crehlegfqllerycgyredsipqledvsrflkertgfqlrpvagllsardflaslafrvfqctqyirhasspmhspepdcchellg 335
                             c+e+le f l++    +  + ipqle++s++l+  tg+++rpvagll  r+fl  laf+ f+ tqyirh+s pm++pepd che+lg
  tr|E5KBU3|E5KBU3_PINTA 151 CKEYLESFPLFN----FSPNKIPQLEELSQILQHYTGWKIRPVAGLLHPRQFLNGLAFKTFHSTQYIRHTSNPMYTPEPDICHEILG 233
                             *********986....78899****************************************************************** PP
```

```
               sequence 336 hvpmladrtfaqfsqdiglaslgasdeeieklstvywftvefglckqngelkaygagllssygellhslseepevrafdpdtaavqp 422
                            h+pml    fa+++q iglaslgasd+ei +l+ +yw+tvefg   +n e+ka+gag+lss+gel h  s +p  + +dp +   +
  tr|E5KBU3|E5KBU3_PINTA 234 HMPMLVHPEFADLAQVIGLASLGASDKEIWHLTKLYWYTVEFGTIEENKEVKAFGAGILSSFGELQHMKSSKPTFQKLDPFAQLPKM 320
                            ********************************************************************************9988777 PP

               sequence 423 yqdqtyqpvyfvsesfndakdklrnyasriq 453
                               + +q +yf+ +sf d+ +klr ya  i+
  tr|E5KBU3|E5KBU3_PINTA 321 SYKDGFQNMYFLCQSFSDTTEKLRSYARTIH 351
                            66778**********************9875 PP

>> tr|A0A5J4XP48|A0A5J4XP48_9CHLO  Tryptophan 5-hydroxylase 1 OS=Trebouxia sp. A1-2 OX=2608996 GN=FRX49_09034 PE=4 SV=1
   #    score  bias  c-Evalue  i-Evalue hmmfrom  hmm to    alifrom  ali to    envfrom  env to     acc
 ---   ------ ----- --------- --------- ------- -------    ------- -------    ------- -------    ----
   1 !  300.1   0.0   8.8e-92   1.4e-86     167     457 ..      63     347 ..      19     355 .. 0.95

  Alignments for each domain:
  == domain 1  score: 300.1 bits;  conditional E-value: 8.8e-92
                        sequence 167 fprkvseldkchhlvtkfdpdldldhpgfsdqvyrqrrkliaeiafqykhgepiphveytaeeiatwkevyvtlkglya 245
                                     +p  +se+d+ +     f  dl  dhpgf d+ y++rr  iae+a +++ g+pip ++yt ee+ tw  v   l  ly
  tr|A0A5J4XP48|A0A5J4XP48_9CHLO  63 IPSSLSEIDNGKI--LGFGADLAADHPGFGDEPYKRRRVSIAELARDHQVGDPIPRIDYTPEEVQTWGTVLRELTQLYP 139
                                     5778889998765..579************************************************************* PP

                        sequence 246 thacrehlegfqllerycgyredsipqledvsrflkertgfqlrpvagllsardflaslafrvfqctqyirhasspmhs 324
                                      hac+e+l  f l++    ++e  +pqled+sr+lk+ tg+q+rpvagll  rdfl  laf  f+ tqy+rh s pm++
  tr|A0A5J4XP48|A0A5J4XP48_9CHLO 140 QHACQEYLHNFPLFN----FKEAEVPQLEDMSRILKNTTGWQIRPVAGLLHPRDFLNGLAFSTFHSTQYVRHHSKPMYT 214
                                     ************985....9*********************************************************** PP

                        sequence 325 pepdcchellghvpmladrtfaqfsqdiglaslgasdeeieklstvywftvefglckqngelkaygagllssygellhs 403
                                     pepd+chellghvpmlad +++++ q+ig+aslga +++i +l+ +ywftvefg++++  ++k +gag+lssygel h
  tr|A0A5J4XP48|A0A5J4XP48_9CHLO 215 PEPDVCHELLGHVPMLADPAYCDLVQNIGVASLGADEKQIWHLTKLYWFTVEFGVVREGDQVKGFGAGVLSSYGELQHM 293
                                     ******************************************************************************* PP

                        sequence 404 lseepevrafdpdtaavqpyqdqtyqpvyfvsesfndakdklrnyasriqrpfs 457
                                      s  p++  fdp +a  +    + yq  yfv +sf++   +l +y+  i  p
  tr|A0A5J4XP48|A0A5J4XP48_9CHLO 294 ASGLPKLVDFDPFAAQPKMSYKDGYQKQYFVLNSFKEGAKQLLDYCLSITDPDV 347
                                     ************999888777789**************99*****999988865 PP

>> tr|I0YV42|I0YV42_COCSC  Aromatic amino acid hydroxylase (Fragment) OS=Coccomyxa subellipsoidea (strain C-169) OX=5745
   #    score  bias  c-Evalue  i-Evalue hmmfrom  hmm to    alifrom  ali to    envfrom  env to     acc
 ---   ------ ----- --------- --------- ------- -------    ------- -------    ------- -------    ----
   1 !  300.2   0.0   8.1e-92   1.2e-86     183     456 ..      12     281 ..       2     293 .. 0.96

  Alignments for each domain:
  == domain 1  score: 300.2 bits;  conditional E-value: 8.1e-92
               sequence 183 kfdpdldldhpgfsdqvyrqrrkliaeiafqykhgepiphveytaeeiatwkevyvtlkglyathacrehlegfqllerycgyreds 269
                             f  dl  dhpgf dq y++rr  ia+ia +++ g+pip +eyt ee+  w+ v   l+ ly   ac e l  + l++    +red
```

```
  tr|I0YV42|I0YV42_COCSC  12 GFGADLAEDHPGFRDQAYKDRRMAIANIARRHRVGDPIPRLEYTPEELRVWRTVLRELHELYPDGACAEFLRSYPLFD----FREDD 94
                             6899*******************************************************************986....9**** PP

                sequence 270 ipqledvsrflkertgfqlrpvagllsardflaslafrvfqctqyirhasspmhspepdcchellghvpmladrtfaqfsqdiglas 356
                             +pqled+s++l+  tg+q+rpvagll  rdfl  laf+ f+ tqy+rh s pm++pepd+chellghvpmlad  f+++ + ig+as
  tr|I0YV42|I0YV42_COCSC  95 VPQLEDLSQVLRRETGWQIRPVAGLLHPRDFLNGLAFQTFHSTQYMRHPSRPMYTPEPDVCHELLGHVPMLADPDFCDMVHSIGVAS 181
                             *************************************************************************************** PP

                sequence 357 lgasdeeieklstvywftvefglckqngelkaygagllssygellhslseepevrafdpdtaavqpyqdqtyqpvyfvsesfndakd 443
                             l a d +i +l+ +ywftvefg++ +  e ka+gag+lssyge+ h  s + +   fdp +   +    + yq  yfv +sf+d  +
  tr|I0YV42|I0YV42_COCSC 182 LCADDSQIWHLTKMYWFTVEFGVVMEGDEKKAFGAGILSSYGEMEHMRSGKADFVPFDPHAKQPKMSYKDGYQNRYFVLNSFKDGAE 268
                             ***********************************************************988777666779**************** PP

                sequence 444 klrnyasriqrpf 456
                             klrny+s i  p
  tr|I0YV42|I0YV42_COCSC 269 KLRNYCSEITPPD 281
                             ********98875 PP

>> tr|E5KBU4|E5KBU4_PHYPA  Chloroplast phenylalanine hydroxylase OS=Physcomitrella patens subsp. patens OX=3218 GN=PHYPA
   #    score  bias  c-Evalue  i-Evalue hmmfrom  hmm to    alifrom  ali to    envfrom  env to     acc
 ---   ------ ----- --------- --------- ------- -------    ------- -------    ------- -------    ----
   1 !  294.4   0.0   4.8e-90   7.4e-85     154     460 ..      80     387 ..      64     392 .. 0.91

  Alignments for each domain:
  == domain 1  score: 294.4 bits;  conditional E-value: 4.8e-90
                sequence 154 ddvrsaredkvpwfprkvseldkchhlvtkfdpdldlhpgfsdqvyrqrrkliaeiafqykhgepiphveytaeeiatwkevyvtl 240
                             +  r a +   p +p  + ++ +  h++  f  dl  dhpg+ d  y++rr  ia++a  +k gepip v+yt+eei  w  v  tl
  tr|E5KBU4|E5KBU4_PHYPA  80 EKEREADKTSTPPIPSSIHDISNGDHIL-GFGADLTEDHPGYHDLEYKRRRSRIADLAKIHKIGEPIPCVDYTSEEIRVWGHVLDTL 165
                             345666677789999********99986.6******************************************************** PP

                sequence 241 kglyathacrehlegfqllerycgyredsipqledvsrflkertgfqlrpvagllsardflaslafrvfqctqyirhasspmhspep 327
                               ly thac+e+l+ ++l++    ++ + ipql+++s +l   tg+++rpvagll  rdfl  lafr f+ tqy+rh s pm++pep
  tr|E5KBU4|E5KBU4_PHYPA 166 VDLYPTHACKEYLNCYELFN----FKPNYIPQLQELSEVLERSTGWHIRPVAGLLHPRDFLNGLAFRTFHSTQYVRHGSNPMYTPEP 248
                             *****************986....77788****************************************************** PP

                sequence 328 dcchellghvpmladrtfaqfsqdiglaslgasdeeieklstvywftvefglckqngelkaygagllssygellhslsee....pev 410
                             d che+lghvp+lad  fa+++  ig aslgas+++i +l+ +yw+tvefg +k+  e+ka+gagllss+gel h         pe
  tr|E5KBU4|E5KBU4_PHYPA 249 DICHEVLGHVPILADPEFADLAWAIGQASLGASEKDIWHLTKLYWYTVEFGTVKEGNEIKAFGAGLLSSFGELKHMRVGTdgfmPEF 335
                             ***************************************************************************764322333999 PP

                sequence 411 rafdpdtaavqpyqdqtyqpvyfvsesfndakdklrnyasriqrp..fsvkf 460
                               +dp     +    + yq  yf+ esf da  klr y+  i +p   s+kf
  tr|E5KBU4|E5KBU4_PHYPA 336 VELDPFKKMPKMSYKDGYQKRYFLCESFADAAAKLRAYSRSILKPevQSIKF 387
                             99***988877666679*************************9994346776 PP

>> tr|A0A383VAB6|A0A383VAB6_TETOB  BH4_AAA_HYDROXYL_2 domain-containing protein OS=Tetradesmus obliquus OX=3088 GN=BQ473
```

```
  #    score  bias  c-Evalue  i-Evalue hmmfrom  hmm to    alifrom  ali to    envfrom  env to     acc
 ---   ------ ----- --------- --------- ------- -------    ------- -------    ------- -------    ----
   1 !  294.0   0.0   6.4e-90   9.8e-85     162     456 ..      47     335 ..      20     338 .] 0.93

  Alignments for each domain:
  == domain 1  score: 294.0 bits;  conditional E-value: 6.4e-90
                       sequence 162 dkvpwfprkvseldkchhlvtkfdpdldldhpgfsdqvyrqrrkliaeiafqykhgepiphveytaeeiatwkevyvtl 240
                                      +  +pr + e+d+ + l   f  +l  dhpgf d+ y+qrr +ia+ia q+  gepip ++yt ee+a w      l
  tr|A0A383VAB6|A0A383VAB6_TETOB  47 PSIVKIPRSIHEVDNGKIL--GFGAELSEDHPGFHDEAYKQRRVMIANIARQHEVGEPIPRIDYTPEEVAVWGTALSKL 123
                                     5566789*******98755..79******************************************************** PP

                       sequence 241 kglyathacrehlegfqllerycgyredsipqledvsrflkertgfqlrpvagllsardflaslafrvfqctqyirhas 319
                                     k l+ +hac e    +    +   +red +pql+d+s +l+  +g+q+rpvagl+  rdfl  laf+ f+ tqy+rhas
  tr|A0A383VAB6|A0A383VAB6_TETOB 124 KALFPSHACAEFNAAL---PK-FNFREDEVPQLQDISEVLQLSSGWQVRPVAGLMHPRDFLNGLAFKYFHSTQYMRHAS 198
                                     **********976654...33.479****************************************************** PP

                       sequence 320 spmhspepdcchellghvpmladrtfaqfsqdiglaslgasdeeieklstvywftvefglckqngelkaygagllssyg 398
                                      p ++pepd++hel+ghvpmlad tfa++ q ig+aslga +++i +l+ vyw+tvefg++++ g++ka+g+g+lss+g
  tr|A0A383VAB6|A0A383VAB6_TETOB 199 KPHYTPEPDVVHELIGHVPMLADPTFAEMVQAIGVASLGADEKQIWHLTKVYWYTVEFGVVREAGDIKAFGSGILSSFG 277
                                     ******************************************************************************* PP

                       sequence 399 ellhslseepevrafdpdtaavqpyqdqtyqpvyfvsesfndakdklrnyasriqrpf 456
                                     el +      e+  fdp +a  +    + yq  yfv +sf+   d+lr++a+ i+  +
  tr|A0A383VAB6|A0A383VAB6_TETOB 278 ELEYMGQGRAELAPFDPFSAQPKMSYKDGYQKRYFVLDSFQTGLDQLRQFAASIKPGV 335
                                     ******************9988777789**********************998655 PP

>> tr|A9T8N9|A9T8N9_PHYPA  Predicted protein (Fragment) OS=Physcomitrella patens subsp. patens OX=3218 GN=PHYPADRAFT_525
  #    score  bias  c-Evalue  i-Evalue hmmfrom  hmm to    alifrom  ali to    envfrom  env to     acc
 ---   ------ ----- --------- --------- ------- -------    ------- -------    ------- -------    ----
   1 !  286.5   0.0   1.2e-87   1.9e-82     184     455 ..       1     272 [.       1     274 [] 0.95

  Alignments for each domain:
  == domain 1  score: 286.5 bits;  conditional E-value: 1.2e-87
               sequence 184 fdpdldldhpgfsdqvyrqrrkliaeiafqykhgepiphveytaeeiatwkevyvtlkglyathacrehlegfqllerycgyredsi 270
                            f  dl  dhpg+ d  y++rr  ia++a  +k gepip v+yt+eei  w  v  tl  ly thac+e+l+ ++l++    ++ + i
  tr|A9T8N9|A9T8N9_PHYPA   1 FGADLTEDHPGYHDLEYKRRRSRIADLAKIHKIGEPIPCVDYTSEEIRVWGHVLDTLVDLYPTHACKEYLNCYELFN----FKPNYI 83
                            67899*********************************************************************986....77788* PP

               sequence 271 pqledvsrflkertgfqlrpvagllsardflaslafrvfqctqyirhasspmhspepdcchellghvpmladrtfaqfsqdiglasl 357
                            pql+++s +l   tg+++rpvagll  rdfl  lafr f+ tqy+rh s pm++pepd che+lghvp+lad  fa+++  ig asl
  tr|A9T8N9|A9T8N9_PHYPA  84 PQLQELSEVLERSTGWHIRPVAGLLHPRDFLNGLAFRTFHSTQYVRHGSNPMYTPEPDICHEVLGHVPILADPEFADLAWAIGQASL 170
                            *************************************************************************************** PP

               sequence 358 gasdeeieklstvywftvefglckqngelkaygagllssygellhslsee....pevrafdpdtaavqpyqdqtyqpvyfvsesfnd 440
                            gas+++i +l+ +yw+tvefg +k+  e+ka+gagllss+gel h         pe   +dp     +    + yq  yf+ esf d
  tr|A9T8N9|A9T8N9_PHYPA 171 GASEKDIWHLTKLYWYTVEFGTVKEGNEIKAFGAGLLSSFGELKHMRVGTdgfmPEFVELDPFKKMPKMSYKDGYQKRYFLCESFAD 257
```

```
                             **********************************************76432233399999***988877666679************* PP

                sequence 441 akdklrnyasriqrp 455
                             a  klr y+  i +p
  tr|A9T8N9|A9T8N9_PHYPA 258 AAAKLRAYSRSILKP 272
                             ********9999887 PP

>> tr|A0A1Y1IAC0|A0A1Y1IAC0_KLENI  Aromatic amino acid hydroxylase OS=Klebsormidium nitens OX=105231 GN=KFL_003840020 PE
   #    score  bias  c-Evalue  i-Evalue hmmfrom  hmm to    alifrom  ali to    envfrom  env to     acc
 ---   ------ ----- --------- --------- ------- -------    ------- -------    ------- -------    ----
   1 !  282.9   0.0   1.5e-86   2.2e-81     167     453 ..      74     359 ..      60     370 .. 0.91

  Alignments for each domain:
  == domain 1  score: 282.9 bits;  conditional E-value: 1.5e-86
                        sequence 167 fprkvseldkchhlvtkfdpdldldhpgfsdqvyrqrrkliaeiafqykhgepiphveytaeeiatwkevyvtlkglya 245
                                     +p+ + e+ +   +   f  +l  dhpg+ d+ y++rr  i e+a q+  g+p+p+veyt  e a w ev   l  ly
  tr|A0A1Y1IAC0|A0A1Y1IAC0_KLENI  74 IPKSINEISNGENI-LGFGANLSADHPGYNDEEYKRRRMQIVELAKQHVPGQPVPYVEYTPAETAVWGEVLTKLEKLYP 151
                                     56777777776654.46999************************************************************ PP

                        sequence 246 thacrehlegfqllerycgyredsipqledvsrflkertgfqlrpvagllsardflaslafrvfqctqyirhasspmhs 324
                                       ac++ l+ f l++    +r d +pql+d+s +l++rtg+ +rpvagll  rdfl  lafr f+ tqyirh s pm++
  tr|A0A1Y1IAC0|A0A1Y1IAC0_KLENI 152 DFACKQFLDTFSLFN----FRPDRVPQLQDMSEVLQQRTGWTIRPVAGLLHPRDFLNGLAFRTFHSTQYIRHGSNPMYT 226
                                     ************985....9*********************************************************** PP

                        sequence 325 pepdcchellghvpmladrtfaqfsqdiglaslgasdeeieklstvywftvefglckqngelkaygagllssygel... 400
                                     pepd chellghvpmlad +fa+++  iglaslgas e+  +l+ +yw+tvefg+ k+  +lka+gag+lss+gel
  tr|A0A1Y1IAC0|A0A1Y1IAC0_KLENI 227 PEPDICHELLGHVPMLADPAFADMAYSIGLASLGASKEDCWHLTKLYWYTVEFGVIKEGEQLKAFGAGILSSFGELewf 305
                                     ***************************************************************************9333 PP

                        sequence 401 lhsls.eepevrafdpdtaavqpyqdqtyqpvyfvsesfndakdklrnyasriq 453
                                      h    + p    +dp     +    + +q  y+v++sf+d   k+++ya+ ++
  tr|A0A1Y1IAC0|A0A1Y1IAC0_KLENI 306 QHGKGdQRPVFEPLDPFGKLTKMSYKDGFQKKYYVADSFEDLSAKIKEYATYLH 359
                                     3554414566778899888777666678**********************9776 PP

>> tr|A0A2P6TZP0|A0A2P6TZP0_CHLSO  Phenylalanine hydroxylase OS=Chlorella sorokiniana OX=3076 GN=C2E21_1449 PE=4 SV=1
   #    score  bias  c-Evalue  i-Evalue hmmfrom  hmm to    alifrom  ali to    envfrom  env to     acc
 ---   ------ ----- --------- --------- ------- -------    ------- -------    ------- -------    ----
   1 !  272.9   0.0   1.6e-83   2.4e-78     167     452 ..      72     351 ..      62     361 .. 0.94

  Alignments for each domain:
  == domain 1  score: 272.9 bits;  conditional E-value: 1.6e-83
                        sequence 167 fprkvseldkchhlvtkfdpdldldhpgfsdqvyrqrrkliaeiafqykhgepiphveytaeeiatwkevyvtlkglya 245
                                     +pr + e+d+ +     f  dl  dhpgf d vy+qrr  i+++a  ++ g+pip ++y+ ee+  w+ v   l+ l+
  tr|A0A2P6TZP0|A0A2P6TZP0_CHLSO  72 IPRSLEEVDNGQI--LGFSADLSEDHPGFHDPVYKQRRVDICNLARTHRIGQPIPRIDYSPEEVDVWRTVMTELQQLFP 148
                                     6899999998765..579************************************************************** PP
```

```
                        sequence 246 thacrehlegfqllerycgyredsipqledvsrflkertgfqlrpvagllsardflaslafrvfqctqyirhasspmhs 324
                                      hac+e l  + +     g+  d +pqled+s +l   tgfq+rpvagll  rdfl  lafr f+ tqy+rh ssp ++
  tr|A0A2P6TZP0|A0A2P6TZP0_CHLSO 149 QHACKEFLRCWPM----FGFSPDEVPQLEDLSAVLTRATGFQIRPVAGLLRPRDFLNGLAFRTFHSTQYMRHHSSPRYT 223
                                     *********9986....59************************************************************ PP

                        sequence 325 pepdcchellghvpmladrtfaqfsqdiglaslgasdeeieklstvywftvefglckqngelkaygagllssygellhs 403
                                     pepd++hel+ghvpmlad ++a++ ++ig+asl a +++i +l+ vyw+tvefg++++ g +ka+gag+lss+gel h
  tr|A0A2P6TZP0|A0A2P6TZP0_CHLSO 224 PEPDVVHELIGHVPMLADPAYADLVHQIGIASLTADEKQIWHLTKVYWYTVEFGVVREGGGVKAFGAGVLSSFGELQHM 302
                                     ******************************************************************************* PP

                        sequence 404 lseepevrafdpdtaavqpyqdqtyqpvyfvsesfndakdklrnyasri 452
                                      +   e+  +d  +    +    + yq  yfv +sf++   +l+ y+  +
  tr|A0A2P6TZP0|A0A2P6TZP0_CHLSO 303 AAGRAELVPLDVAAPLPRMSYKDGYQQRYFVLDSFEEGAAHLKAYCRTL 351
                                     *******99987766655555668********************98765 PP

>> tr|A0A087SFZ0|A0A087SFZ0_AUXPR  Tryptophan 5-hydroxylase 1 OS=Auxenochlorella protothecoides OX=3075 GN=APUTEX25_0051
   #    score  bias  c-Evalue  i-Evalue hmmfrom  hmm to    alifrom  ali to    envfrom  env to     acc
 ---   ------ ----- --------- --------- ------- -------    ------- -------    ------- -------    ----
   1 !  271.7   0.0   3.7e-83   5.8e-78     167     457 ..      32     316 ..      19     323 .. 0.95

  Alignments for each domain:
  == domain 1  score: 271.7 bits;  conditional E-value: 3.7e-83
                        sequence 167 fprkvseldkchhlvtkfdpdldldhpgfsdqvyrqrrkliaeiafqykhgepiphveytaeeiatwkevyvtlkglya 245
                                     +p+ +se+d+ + l   f  +l   hpgf+d+ y+qrr  i++ia ++  g pip +eytaee a w  v   l  l+
  tr|A0A087SFZ0|A0A087SFZ0_AUXPR  32 IPKSLSEVDNGKIL--GFGAELAEGHPGFADEAYKQRRVDICKIASKHEIGAPIPAIEYTAEETAVWSYVMNELAELFP 108
                                     68999999998755..79************************************************************* PP

                        sequence 246 thacrehlegfqllerycgyredsipqledvsrflkertgfqlrpvagllsardflaslafrvfqctqyirhasspmhs 324
                                     +hac e l  +    r  g+    +pql++vs fl+ r+g+q+rpvagll  rdfl  lafr f+ tqy+rh + p  +
  tr|A0A087SFZ0|A0A087SFZ0_AUXPR 109 AHACSEFLASW----RELGFSPRRVPQLQEVSEFLQARSGWQIRPVAGLLHPRDFLNGLAFRTFHSTQYMRHGADPCWT 183
                                     *******9998....5669999********************************************************* PP

                        sequence 325 pepdcchellghvpmladrtfaqfsqdiglaslgasdeeieklstvywftvefglckqngelkaygagllssygellhs 403
                                     pepd +he++ghvpmla  +f+q+++ ig+aslga + +i +l+  yw+tvefg++++ g +ka+gag+lssyge+
  tr|A0A087SFZ0|A0A087SFZ0_AUXPR 184 PEPDIVHEMIGHVPMLAHPAFCQLAHAIGVASLGADEAQIWHLTKCYWYTVEFGVVREGGAVKAFGAGVLSSYGEMKNM 262
                                     ******************************************************************************* PP

                        sequence 404 lseepevrafdpdtaavqpyqdqtyqpvyfvsesfndakdklrnyasriqrpfs 457
                                      s    +r fdp     +    + yq  y v +sf+d   kl  y+  ++ p+s
  tr|A0A087SFZ0|A0A087SFZ0_AUXPR 263 ASGRAGLREFDPFQKQPKMSYSDGYQKTYTVLDSFEDGAAKLEAYCKTLHVPLS 316
                                     ************988877777789**********************9999988 PP

>> tr|A0A1D1ZM34|A0A1D1ZM34_AUXPR  BH4_AAA_HYDROXYL_2 domain-containing protein (Fragment) OS=Auxenochlorella prototheco
   #    score  bias  c-Evalue  i-Evalue hmmfrom  hmm to    alifrom  ali to    envfrom  env to     acc
 ---   ------ ----- --------- --------- ------- -------    ------- -------    ------- -------    ----
   1 !  270.5   0.0   8.2e-83   1.3e-77     167     457 ..      84     368 ..      72     374 .. 0.96
```

```
  Alignments for each domain:
  == domain 1  score: 270.5 bits;  conditional E-value: 8.2e-83
                      sequence 167 fprkvseldkchhlvtkfdpdldlhpgfsdqvyrqrrkliaeiafqykhgepiphveytaeeiatwkevyvtlkglya 245
                                   +p+ +se+d+ + l   f  +l   hpgf+d+ y+qrr  i++ia ++  g pip +eytaee a w  v   l  l+
  tr|A0A1D1ZM34|A0A1D1ZM34_AUXPR  84 IPKSLSEVDNGKIL--GFGAELAEGHPGFADEAYKQRRVDICKIASKHEIGAPIPAIEYTAEETAVWSYVMNELAELFP 160
                                   68999999998755..79************************************************************* PP

                      sequence 246 thacrehlegfqllerycgyredsipqledvsrflkertgfqlrpvagllsardflaslafrvfqctqyirhasspmhs 324
                                   +hac e l  +    r  g+    +pql++vs fl+ r+g+q+rpvagll  rdfl  lafr f+ tqy+rh + p  +
  tr|A0A1D1ZM34|A0A1D1ZM34_AUXPR 161 AHACSEFLASW----RELGFSPRRVPQLQEVSEFLQARSGWQIRPVAGLLHPRDFLNGLAFRTFHSTQYMRHGADPCWT 235
                                   *******9998....5669999********************************************************* PP

                      sequence 325 pepdcchellghvpmladrtfaqfsqdiglaslgasdeeieklstvywftvefglckqngelkaygagllssygellhs 403
                                   pepd +he++ghvpmla  +f+q+++ ig+aslga + +i +l+  yw+tvefg++++ g +ka+gag+lssyge+
  tr|A0A1D1ZM34|A0A1D1ZM34_AUXPR 236 PEPDIVHEMIGHVPMLAHPAFCQLAHAIGVASLGADEAQIWHLTKCYWYTVEFGVVREGGGVKAFGAGVLSSYGEMKNM 314
                                   ******************************************************************************* PP

                      sequence 404 lseepevrafdpdtaavqpyqdqtyqpvyfvsesfndakdklrnyasriqrpfs 457
                                    s    +r fdp     +    + yq  y v +sf+d   kl  y+  ++ p+s
  tr|A0A1D1ZM34|A0A1D1ZM34_AUXPR 315 ASGRAGLREFDPFQKQPKMSYSDGYQKTYTVLDSFEDGAAKLEAYCKTLHVPLS 368
                                   ************988877777789***********************9999987 PP

>> tr|A0A5B8MVV0|A0A5B8MVV0_9CHLO  Aromatic amino acid hydroxylase OS=Chloropicon primus OX=1764295 GN=A3770_13p68940 PE
   #    score  bias  c-Evalue  i-Evalue hmmfrom  hmm to    alifrom  ali to    envfrom  env to     acc
 ---   ------ ----- --------- --------- ------- -------    ------- -------    ------- -------    ----
   1 !  270.5   0.0   8.3e-83   1.3e-77     159     450 ..      66     351 ..      48     356 .. 0.94

  Alignments for each domain:
  == domain 1  score: 270.5 bits;  conditional E-value: 8.3e-83
                      sequence 159 aredkvpwfprkvseldkchhlvtkfdpdldlhpgfsdqvyrqrrkliaeiafqykhgepiphveytaeeiatwkevy 237
                                    +++ v  +p  + ++d+ +     fd +l  dhpgf d+ y+qrr  ia +a +   g+ ip +ey  ee+ tw +
  tr|A0A5B8MVV0|A0A5B8MVV0_9CHLO  66 EQNEEVRVIPTSIHDIDNGKI--LGFDHNLSEDHPGFKDEDYKQRRSWIAGLARDILPGDEIPRLEYRPEEVETWAKAL 142
                                   566778888999999998765..57****************************************************** PP

                      sequence 238 vtlkglyathacrehlegfqllerycgyredsipqledvsrflkertgfqlrpvagllsardflaslafrvfqctqyir 316
                                     l  l+ thac+e l+ ++ l+    +r d +pql+dv ++l+e +gf +rpvagll  rdfl  laf+ f+ tqy+r
  tr|A0A5B8MVV0|A0A5B8MVV0_9CHLO 143 EELEQLFPTHACKEFLDAIDKLD----FRPDVVPQLQDVHQVLQETSGFAIRPVAGLLHPRDFLNGLAFKTFHSTQYMR 217
                                   ******************99886....9*************************************************** PP

                      sequence 317 hasspmhspepdcchellghvpmladrtfaqfsqdiglaslgasdeeieklstvywftvefglckqngelkaygaglls 395
                                   h s p ++pepd  he++gh+pml+++ +a++ q+iglasl a+d+ei +l+ vyw+tvefg++ ++ e+ka+gag+ls
  tr|A0A5B8MVV0|A0A5B8MVV0_9CHLO 218 HWSQPHYTPEPDLIHEIIGHIPMLTNKYYADMVQQIGLASLYATDKEIWHLTKVYWYTVEFGVVMEDEEIKAFGAGVLS 296
                                   ******************************************************************************* PP

                      sequence 396 sygellhslseepevrafdpdtaavqpyqdqtyqpvyfvsesfndakdklrnyas 450
```

```
                                  sygel h  + +pe   +dp +   +    + +q  yfv +sf++  + lr+y+
  tr|A0A5B8MVV0|A0A5B8MVV0_9CHLO 297 SYGELDHMRTGNPEFAPLDPWAKQPKMSYKDGFQKKYFVLNSFEEGSELLREYSQ 351
                                  ********************987766666678**********************85 PP

>> tr|A0A2P6VBN6|A0A2P6VBN6_9CHLO  Chloroplast phenylalanine hydroxylase OS=Micractinium conductrix OX=554055 GN=C2E20_5
   #    score  bias  c-Evalue  i-Evalue hmmfrom  hmm to    alifrom  ali to    envfrom  env to     acc
 ---   ------ ----- --------- --------- ------- -------    ------- -------    ------- -------    ----
   1 !  268.5   0.0   3.3e-82   5.1e-77     160     454 ..      44     333 ..      18     340 .. 0.91

  Alignments for each domain:
  == domain 1  score: 268.5 bits;  conditional E-value: 3.3e-82
                        sequence 160 redkvp.wfprkvseldkchhlvtkfdpdldldhpgfsdqvyrqrrkliaeiafqykhgepiphveytaeeiatwkevy 237
                                      e+  p  +p  + e+d+    +  f  +l  dhpgf d  y+qrr  i+++a  ++ gepiph+ ytaee+a w
  tr|A0A2P6VBN6|A0A2P6VBN6_9CHLO  44 DEQAAPrRVPLGIQEVDNGA--ILGFSAELSEDHPGFHDAAYKQRRVNICNLARSHRIGEPIPHISYTAEEVAVWGSAL 120
                                     45555423566677777664..55799****************************************************** PP

                        sequence 238 vtlkglyathacrehlegfqllerycgyredsipqledvsrflkertgfqlrpvagllsardflaslafrvfqctqyir 316
                                       l  l+  hac+e    + l++     r d++pqled+s++l+  tgf++rpvagll  rdfl  lafr f+ tqy+r
  tr|A0A2P6VBN6|A0A2P6VBN6_9CHLO 121 TRLEHLFQQHACKEFQRSWPLFD----LRPDAVPQLEDLSQVLQGTTGFRIRPVAGLLHPRDFLNGLAFRTFHSTQYMR 195
                                     **************999988876....79**************************************************** PP

                        sequence 317 hasspmhspepdcchellghvpmladrtfaqfsqdiglaslgasdeeieklstvywftvefglckqngelkaygagllls 395
                                     h+s p ++pepd+ he++gh+pmlad +fa +++ ig+asl a +e++++l  +yw+tvefg++++  ++ka+gag+ls
  tr|A0A2P6VBN6|A0A2P6VBN6_9CHLO 196 HSSKPDYTPEPDVIHEVIGHLPMLADPSFASLAHAIGVASLAADEEQLKHLVKLYWYTVEFGVVREGSDVKAFGAGILS 274
                                     ******************************************************************************** PP

                        sequence 396 sygellhslseepevrafdpdtaavqpyqdqtyqpvyfvsesfndakdklrnyasriqr 454
                                     sygel h  +   ev  +d      +    + yq  yf  esf+     l+ y++ +q
  tr|A0A2P6VBN6|A0A2P6VBN6_9CHLO 275 SYGELQHMAAGGAEVAPLDVWQPLPKISYKDGYQKRYFALESFEAGAVELQAYCASLQA 333
                                     *******************9887776666668*********************999886 PP

>> tr|A0A2P6V2S1|A0A2P6V2S1_9CHLO  Chloroplast phenylalanine hydroxylase OS=Micractinium conductrix OX=554055 GN=C2E20_8
   #    score  bias  c-Evalue  i-Evalue hmmfrom  hmm to    alifrom  ali to    envfrom  env to     acc
 ---   ------ ----- --------- --------- ------- -------    ------- -------    ------- -------    ----
   1 !  267.0   0.0   9.8e-82   1.5e-76     181     454 ..      86     355 ..      69     363 .. 0.94

  Alignments for each domain:
  == domain 1  score: 267.0 bits;  conditional E-value: 9.8e-82
                        sequence 181 vtkfdpdldldhpgfsdqvyrqrrkliaeiafqykhgepiphveytaeeiatwkevyvtlkglyathacrehlegfqll 259
                                     +  f  +l  dhpgf d  y+qrr  i+++a  ++ gepiph+ ytaee+a w      l  l+  hac+e    + l+
  tr|A0A2P6V2S1|A0A2P6V2S1_9CHLO  86 ILGFSAELSEDHPGFHDAAYKQRRVNICNLARSHRIGEPIPHISYTAEEVAVWGSALTRLEHLFQQHACKEFQRSWPLF 164
                                     4569999*****************************************************************99998887 PP

                        sequence 260 erycgyredsipqledvsrflkertgfqlrpvagllsardflaslafrvfqctqyirhasspmhspepdcchellghvp 338
                                     +     r d++pqled+s++l+  tgf++rpvagll  rdfl  lafr f+ tqy+rh+s p ++pepd+ he++gh+p
  tr|A0A2P6V2S1|A0A2P6V2S1_9CHLO 165 D----LRPDAVPQLEDLSQVLQGTTGFRIRPVAGLLHPRDFLNGLAFRTFHSTQYMRHSSKPDYTPEPDVIHEVIGHLP 239
```

```
                               6....79************************************************************************ PP

                  sequence 339 mladrtfaqfsqdiglaslgasdeeieklstvywftvefglckqngelkaygagllssygellhslseepevrafdpdt 417
                               mlad +fa +++ ig+asl a +e++++l  +yw+tvefg++++  ++ka+gag+lssygel h  +   ev  +d
  tr|A0A2P6V2S1|A0A2P6V2S1_9CHLO 240 MLADPSFASLAHAIGVASLAADEEQLKHLVKLYWYTVEFGVVREGSDVKAFGAGILSSYGELQHMAAGGAEVAPLDVWQ 318
                               ****************************************************************************988 PP

                  sequence 418 aavqpyqdqtyqpvyfvsesfndakdklrnyasriqr 454
                                  +    + yq  yf  esf+     l+ y++ +q
  tr|A0A2P6V2S1|A0A2P6V2S1_9CHLO 319 PLPKISYKDGYQKRYFALESFEAGAVELQAYCASLQA 355
                               7776666668*********************999986 PP

>> tr|A8HQD7|A8HQD7_CHLRE  Aromatic amino acid hydroxylase-related protein OS=Chlamydomonas reinhardtii OX=3055 GN=AAH1
   #    score  bias  c-Evalue  i-Evalue hmmfrom  hmm to    alifrom  ali to    envfrom  env to     acc
 ---   ------ ----- --------- --------- ------- -------    ------- -------    ------- -------    ----
   1 !  264.8   0.0   4.5e-81   6.9e-76     179     461 ..      79     357 ..      49     360 .. 0.94

  Alignments for each domain:
  == domain 1  score: 264.8 bits;  conditional E-value: 4.5e-81
              sequence 179 hlvtkfdpdldldhpgfsdqvyrqrrkliaeiafqykhgepiphveytaeeiatwkevyvtlkglyathacrehlegfqllerycgy 265
                             +  f  dl  dhpgf d  y+qrr  +ae+a  ++ g pip vey+  e+atw  v   l gl   hacre+l  + l++    +
  tr|A8HQD7|A8HQD7_CHLRE  79 GQILGFGADLAEDHPGFHDPAYKQRRAWLAEMAKTHRIGTPIPDVEYSPAEVATWDAVLEELSGLLPQHACREYLRCLTLFD----F 161
                           45667999*********************************************************************99986....8 PP

              sequence 266 redsipqledvsrflkertgfqlrpvagllsardflaslafrvfqctqyirhasspmhspepdcchellghvpmladrtfaqfsqdi 352
                           r+  +pqle++  +l+  tg+ +rpvagl+  r fla laf+ f+ tqy+rh s p ++pepd++hel+ghvp+lad ++a++ q i
  tr|A8HQD7|A8HQD7_CHLRE 162 RKGRVPQLEEMNTVLRSTTGWTVRPVAGLMHPRHFLAGLAFKHFHSTQYMRHPSKPSYTPEPDVVHELIGHVPLLADPAYARLIQTI 248
                           999************************************************************************************ PP

              sequence 353 glaslgasdeeieklstvywftvefglckqngelkaygagllssygellhslseepevrafdpdtaavqpyqdqtyqpvyfvsesfn 439
                           glasl a d++i +l+ vyw tvefg++++  ++ka+gag+lssygel h  s    +  +dp     +    + +q  yfv +sf
  tr|A8HQD7|A8HQD7_CHLRE 249 GLASLAADDKQIWHLTKVYWHTVEFGVVREGDQVKAFGAGILSSYGELAHMASGAAALERLDPFRPQPRMAYKDGFQKRYFVLDSFA 335
                           ****************************************************************988776666668*********** PP

              sequence 440 dakdklrnyasriqrpfsvkfd 461
                           +  + l  ya+ +  p s++ d
  tr|A8HQD7|A8HQD7_CHLRE 336 EGSELLSSYAASLGLPESLRGD 357
                           ***********99999888765 PP

>> tr|A0A061R0L5|A0A061R0L5_9CHLO  Tryptophan 5-monooxygenase OS=Tetraselmis sp. GSL018 OX=582737 GN=TPH PE=4 SV=1
   #    score  bias  c-Evalue  i-Evalue hmmfrom  hmm to    alifrom  ali to    envfrom  env to     acc
 ---   ------ ----- --------- --------- ------- -------    ------- -------    ------- -------    ----
   1 !  264.5   0.0   5.6e-81   8.6e-76     156     453 ..      52     343 ..      36     348 .. 0.92

  Alignments for each domain:
  == domain 1  score: 264.5 bits;  conditional E-value: 5.6e-81
```

```
                        sequence 156 vrsaredkvpwfprkvseldkchhlvtkfdpdldlhpgfsdqvyrqrrkliaeiafqykhgepiphveytaeeiatwk 234
                                     v  a  +    +p+ + ++d+ +     f  +l  +hpg++d+ y++rr  i  +a  +  g+pip ++yt +ei +w
  tr|A0A061R0L5|A0A061R0L5_9CHLO  52 VLQATSNVESCVPQSIHDIDNGKI--LGFGEELGPEHPGYTDEDYKKRRAEIVRLAHYHEIGQPIPRLQYTEDEIRAWG 128
                                     444555555667888888887764..47999************************************************ PP

                        sequence 235 evyvtlkglyathacrehlegfqllerycgyredsipqledvsrflkertgfqlrpvagllsardflaslafrvfqctq 313
                                          l+ ly thac+e    ++ +e    ++e  +pql+dvs +lk  tg+q+rpvagll  rdfl  laf  f+ tq
  tr|A0A061R0L5|A0A061R0L5_9CHLO 129 IALRELQALYPTHACQEFRRNWDHFE----FKESEVPQLQDVSEILKCSTGWQIRPVAGLLHPRDFLNGLAFNTFHSTQ 203
                                     *****************999888766....9************************************************ PP

                        sequence 314 yirhasspmhspepdcchellghvpmladrtfaqfsqdiglaslgasdeeieklstvywftvefglckqngelkaygag 392
                                     y+rh s pm++pepd+ hellghv mlad  ++++ + ig asl asd+ei +l+ +yw+tvefg +k+  e++a+gag
  tr|A0A061R0L5|A0A061R0L5_9CHLO 204 YMRHHSEPMYTPEPDVIHELLGHVVMLADPVYCELVNTIGRASLAASDKEIWHLTKIYWYTVEFGTVKEGNEIRAFGAG 282
                                     ******************************************************************************* PP

                        sequence 393 llssygellhslseepevrafdpdtaavqpyqdqtyqpvyfvsesfndakdklrnyasriq 453
                                     llssygel h  s   +   fdp +   +    + yq  yf+ +sf+d   +l+++a+ +
  tr|A0A061R0L5|A0A061R0L5_9CHLO 283 LLSSYGELEHMRSGRAKFEPFDPFAKQPKMSYKDGYQERYFLMDSFEDGCRQLKEFAATMT 343
                                     ***********************987766666679*********************98765 PP

>> tr|D8U9W1|D8U9W1_VOLCA  BH4_AAA_HYDROXYL_2 domain-containing protein OS=Volvox carteri f. nagariensis OX=3068 GN=VOLC
   #    score  bias  c-Evalue  i-Evalue hmmfrom  hmm to    alifrom  ali to    envfrom  env to     acc
 ---   ------ ----- --------- --------- ------- -------    ------- -------    ------- -------    ----
   1 !  261.6   0.0   4.2e-80   6.4e-75     183     462 ..      13     292 ..       6     295 .] 0.96

  Alignments for each domain:
  == domain 1  score: 261.6 bits;  conditional E-value: 4.2e-80
                sequence 183 kfdpdldldhpgfsdqvyrqrrkliaeiafqykhgepiphveytaeeiatwkevyvtlkglyathacrehlegfqllerycgyreds 269
                              f  dl  dhpg+ d+ y+ rr ++ae a q+  g  ip v ytaee+atw+ v   l+ l   hac+e+l  + l++    +r
  tr|D8U9W1|D8U9W1_VOLCA  13 GFGADLADDHPGYHDEAYKVRRTMLAEAAKQHVIGTSIPDVVYTAEEVATWNIVLEQLQDLLPRHACKEYLRCLPLFN----FRPGK 95
                             6899*********************************************************************99975....89999 PP

                sequence 270 ipqledvsrflkertgfqlrpvagllsardflaslafrvfqctqyirhasspmhspepdcchell....ghvpmladrtfaqfsqdi 352
                             +pqle++ r+l+  tg+++rpvagl+  r fla laf+ f+ tqy+rh s p ++pepd++hel+    ghvpmlad +f+++ q i
  tr|D8U9W1|D8U9W1_VOLCA  96 VPQLEEMNRVLRSTTGWNIRPVAGLMHPRHFLAGLAFKHFHSTQYMRHPSKPSYTPEPDVVHELIgryvGHVPMLADPAFCRLVQAI 182
                             ***************************************************************9633337***************** PP

                sequence 353 glaslgasdeeieklstvywftvefglckqngelkaygagllssygellhslseepevrafdpdtaavqpyqdqtyqpvyfvsesfn 439
                             g aslga d+ i +l+ vyw+tvefg++++ g +ka+gag+lss+gel h  s   e++ +dp     +    + yq  yf  +sf+
  tr|D8U9W1|D8U9W1_VOLCA 183 GAASLGADDKTIWHLTKVYWYTVEFGVVREGGSIKAFGAGILSSFGELAHMASGVAELQPLDPFRPLPRMSYKDGYQNRYFCLDSFE 269
                             ***************************************************************998877666679************ PP

                sequence 440 dakdklrnyasriqrpfsvkfdp 462
                                + l++ya+ +  p s++ dp
  tr|D8U9W1|D8U9W1_VOLCA 270 SGTQPLQDYAAAMALPDSLRGDP 292
                             ******************99998 PP
```

```
>> tr|A0A250WYI1|A0A250WYI1_9CHLO  BH4_AAA_HYDROXYL_2 domain-containing protein OS=Chlamydomonas eustigma OX=1157962 GN=
   #    score  bias  c-Evalue  i-Evalue hmmfrom  hmm to    alifrom  ali to    envfrom  env to     acc
 ---   ------ ----- --------- --------- ------- -------    ------- -------    ------- -------    ----
   1 !  256.2   0.0   1.8e-78   2.8e-73     175     455 ..      88     364 ..      69     371 .. 0.95

  Alignments for each domain:
  == domain 1  score: 256.2 bits;  conditional E-value: 1.8e-78
                        sequence 175 dkchhlvtkfdpdldldhpgfsdqvyrqrrkliaeiafqykhgepiphveytaeeiatwkevyvtlkglyathacrehl 253
                                     d     +  f  +l  dhpgf d  y+ rr  i+e+a  +k g pip + y  ee+  wk v   l+ l   hac+++l
  tr|A0A250WYI1|A0A250WYI1_9CHLO  88 DVSNNNILGFGANLSQDHPGFNDLSYKARRAWISELASAHKIGMPIPRIAYNQEELQVWKTVLQELRDLLPQHACKQYL 166
                                     455566778999******************************************************************* PP

                        sequence 254 egfqllerycgyredsipqledvsrflkertgfqlrpvagllsardflaslafrvfqctqyirhasspmhspepdcche 332
                                     +   l++    +  + ipql+dv  +l+  tg+ +rp agll  rdfla laf+ f+ tqyirh ssp ++pepd +he
  tr|A0A250WYI1|A0A250WYI1_9CHLO 167 QAAPLFN----FTPNQIPQLQDVNEVLNRATGWSIRPAAGLLHPRDFLAGLAFKTFHSTQYIRHPSSPSYTPEPDLVHE 241
                                     *999875....78899*************************************************************** PP

                        sequence 333 llghvpmladrtfaqfsqdiglaslgasdeeieklstvywftvefglckqngelkaygagllssygellhslseepevr 411
                                     l+ghvp+lad +fa++ q +g aslg  d++i +l  vyw+tvefg++++   +ka+gag+lss+gel h      +
  tr|A0A250WYI1|A0A250WYI1_9CHLO 242 LIGHVPLLADAAFADMLQTLGEASLGVDDKQIWHLIKVYWYTVEFGVVREGSCVKAFGAGILSSFGELQHMSKGTAQFE 320
                                     ******************************************************************************* PP

                        sequence 412 afdpdtaavqpyqdqtyqpvyfvsesfndakdklrnyasriqrp 455
                                     ++dp  a  +    + +q  yfv e+f+  +d l++ya + + p
  tr|A0A250WYI1|A0A250WYI1_9CHLO 321 TLDPFIAQPKMSYKDGFQNRYFVLETFEAGRDMLQDYAMKAKLP 364
                                     ******9988777779**********************998776 PP

>> tr|E1ZIW4|E1ZIW4_CHLVA  BH4_AAA_HYDROXYL_2 domain-containing protein (Fragment) OS=Chlorella variabilis OX=554065 GN=
   #    score  bias  c-Evalue  i-Evalue hmmfrom  hmm to    alifrom  ali to    envfrom  env to     acc
 ---   ------ ----- --------- --------- ------- -------    ------- -------    ------- -------    ----
   1 !  256.2   0.0   1.9e-78   2.9e-73     187     410 ..       2     221 ..       1     235 [. 0.96

  Alignments for each domain:
  == domain 1  score: 256.2 bits;  conditional E-value: 1.9e-78
                sequence 187 dldldhpgfsdqvyrqrrkliaeiafqykhgepiphveytaeeiatwkevyvtlkglyathacrehlegfqllerycgyredsipql 273
                             dl  dhpg+ d  y+qrr  i+++a +++ gepip +eyt +e+a w  v   lk l   hac+e l  +ql++    +red +pql
  tr|E1ZIW4|E1ZIW4_CHLVA   2 DLSEDHPGYLDTFYKQRRADICNLAREHRIGEPIPRIEYTPDEVAVWGTVLPQLKELIPRHACKEFLRCWQLFD----FREDEVPQL 84
                             8999********************************************************************86....9******** PP

                sequence 274 edvsrflkertgfqlrpvagllsardflaslafrvfqctqyirhasspmhspepdcchellghvpmladrtfaqfsqdiglaslgas 360
                             ed+s +l+++tgf++rpvagll  r fl  la++ f+ tqy+rh s p ++pepd++hel+ghvpmlad +fa++ + ig+aslga
  tr|E1ZIW4|E1ZIW4_CHLVA  85 EDLSLVLQQQTGFRIRPVAGLLHPRLFLQGLAYKTFHSTQYMRHVSRPDYTPEPDVVHELIGHVPMLADPAFAELVHAIGIASLGAD 171
                             *************************************************************************************** PP

                sequence 361 deeieklstvywftvefglckqngelkaygagllssygellhslseepev 410
```

```
                             +++i +l+ +yw+tvefg++++ g++ka+gag+lssygel h  s    +
  tr|E1ZIW4|E1ZIW4_CHLVA 172 EKQIWHLTKIYWYTVEFGVVREGGDVKAFGAGILSSYGELQHMASGAARL 221
                             ******************************************98876554 PP

>> tr|A0A1Y1I6U4|A0A1Y1I6U4_KLENI  Aromatic amino acid hydroxylase OS=Klebsormidium nitens OX=105231 GN=KFL_001890200 PE
   #    score  bias  c-Evalue  i-Evalue hmmfrom  hmm to    alifrom  ali to    envfrom  env to     acc
 ---   ------ ----- --------- --------- ------- -------    ------- -------    ------- -------    ----
   1 !  252.0   0.0   3.5e-77   5.4e-72     185     454 ..     166     436 ..     142     437 .] 0.91

  Alignments for each domain:
  == domain 1  score: 252.0 bits;  conditional E-value: 3.5e-77
                       sequence 185 dpdldldhpgfsdqvyrqrrkliaeiafqykhgepiphveytaeeiatwkevyvtlkglyathacrehlegfqlleryc 263
                                    d +l  dhpgf d+ y +rr+ +a ia ++k g pip ++y+ ee   w  v   l+ l+ tha  ++   ++ l
  tr|A0A1Y1I6U4|A0A1Y1I6U4_KLENI 166 DQNLPSDHPGFNDEKYVRRRRELATIARNHKFGRPIPRIDYSPEETYVWTTVLNKLRELHPTHASSKYNAAVKKL---- 240
                                    456788*******************************************************9988887766.... PP

                       sequence 264 gyredsipqledvsrflkertgfqlrpvagllsardflaslafrvfqctqyirhasspmhspepdcchellghvpmlad 342
                                     +r ++ipqledvs ++ ++tg+++rpvagl+  rdf+ s+af++f+ctqy+rh s pm+spepd chel+gh pm+ d
  tr|A0A1Y1I6U4|A0A1Y1I6U4_KLENI 241 DFRPNAIPQLEDVSAVMYQQTGWRIRPVAGLMDPRDFIGSMAFKIFHCTQYVRHHSQPMYSPEPDLCHELIGHAPMFLD 319
                                    59***************************************************************************** PP

                       sequence 343 rtfaqfsqdiglaslgasdeeieklstvywftvefglckqngelkaygagllssygellhslsee....pevrafdpdt 417
                                      f+++ q ig aslga++ee  +l+ ++w+t efg+  ++ ++ka+gagllss+gel +         p  ra+
  tr|A0A1Y1I6U4|A0A1Y1I6U4_KLENI 320 PEFSDMVQAIGQASLGATNEEFVQLTRLFWYTCEFGVIEEDNQMKAFGAGLLSSFGELAYMRDGHegvmPRFRALGDPR 398
                                    **********************************************************88654321334899**97777 PP

                       sequence 418 aavqpyqdq.tyqpvyfvsesfndakdklrnyasriqr 454
                                     ++ p + +  +q  y+vs+ f d  +kl  +a +i+r
  tr|A0A1Y1I6U4|A0A1Y1I6U4_KLENI 399 ITLPPMRHKpGFQTRYMVSNGFADVSEKLSTFAEHIER 436
                                    88888876537***********************9998 PP

>> tr|A0A2J8AD84|A0A2J8AD84_9CHLO  Tryptophan 5-hydroxylase 1 OS=Tetrabaena socialis OX=47790 GN=TSOC_002790 PE=4 SV=1
   #    score  bias  c-Evalue  i-Evalue hmmfrom  hmm to    alifrom  ali to    envfrom  env to     acc
 ---   ------ ----- --------- --------- ------- -------    ------- -------    ------- -------    ----
   1 !  251.1   0.0   6.3e-77   9.7e-72     172     461 ..      45     328 ..      19     331 .. 0.94

  Alignments for each domain:
  == domain 1  score: 251.1 bits;  conditional E-value: 6.3e-77
                       sequence 172 seldkchhlvtkfdpdldldhpgfsdqvyrqrrkliaeiafqykhgepiphveytaeeiatwkevyvtlkglyathacr 250
                                     ++d+ +  +  f  dl  dhpg+ d+ y++rr  +ae a ++  g pip v y  ee+atw  v   l  l   hac+
  tr|A0A2J8AD84|A0A2J8AD84_9CHLO  45 HDVDNSQ--ILGFGADLADDHPGYHDEAYKRRRSALAEAAKRHVVGTPIPDVSYGPEEVATWDAVLAELSELLPRHACK 121
                                    4555443..55799***************************************************************** PP

                       sequence 251 ehlegfqllerycgyredsipqledvsrflkertgfqlrpvagllsardflaslafrvfqctqyirhasspmhspepdc 329
                                    e+l  + l++    +r   +pql ++  +l   tg+ +rpvagll  r fla laf+ f+ tqy+rh s p ++pepd
  tr|A0A2J8AD84|A0A2J8AD84_9CHLO 122 EYLRCLPLFN----FRPGKVPQLSEINGVLAGTTGWTVRPVAGLLHPRHFLAGLAFKHFHSTQYMRHPSKPSYTPEPDI 196
```

```
                                     *****99975....89999************************************************************ PP

                      sequence 330 chellghvpmladrtfaqfsqdiglaslgasdeeieklstvywftvefglckqngelkaygagllssygellhslseep 408
                                   +hel+ghvp+lad +++++ q ig aslga d+ i +l+ vywft+efg++++ g++ka+gag+lssygel h  s    
  tr|A0A2J8AD84|A0A2J8AD84_9CHLO 197 VHELIGHVPLLADPSYSRLIQAIGRASLGADDKAIWHLTKVYWFTIEFGVVRERGDIKAFGAGILSSYGELAHMASGAA 275
                                     ******************************************************************************* PP

                      sequence 409 evrafdpdtaavqpyqdqtyqpvyfvsesfndakdklrnyasriqrpfsvkfd 461
                                    +  +dp     +    + +q  yf+ +sf+   + l+ ya  +  p s++ d 
  tr|A0A2J8AD84|A0A2J8AD84_9CHLO 276 ALEPLDPFRPQPRMSYKDGFQKRYFLLDSFEGGAELLQRYAENLALPESLRGD 328
                                     *******988776666668************************9999888766 PP

>> tr|A0A0D2MT76|A0A0D2MT76_9CHLO  Phenylalanine-4-hydroxylase OS=Monoraphidium neglectum OX=145388 GN=MNEG_4320 PE=4 SV
   #    score  bias  c-Evalue  i-Evalue hmmfrom  hmm to    alifrom  ali to    envfrom  env to     acc
 ---   ------ ----- --------- --------- ------- -------    ------- -------    ------- -------    ----
   1 !  248.4   0.0   4.4e-76   6.7e-71     168     417 ..      50     289 ..      19     301 .. 0.92

  Alignments for each domain:
  == domain 1  score: 248.4 bits;  conditional E-value: 4.4e-76
                      sequence 168 prkvseldkchhlvtkfdpdldldhpgfsdqvyrqrrkliaeiafqykhgepiphveytaeeiatwkevyvtlkglyat 246
                                   p  + e+d+ +  +  f  dl  dhpgf d+ y+qrr  ia+ia ++  g+pip ++yt +e+ +w      lk l+ t 
  tr|A0A0D2MT76|A0A0D2MT76_9CHLO  50 PSSIHEVDNGK--ILGFGADLAEDHPGFGDEAYKQRRAAIADIARRHEIGDPIPRIDYTPDEVRAWGTALRELKLLFPT 126
                                     55555666554..55799************************************************************* PP

                      sequence 247 hacrehlegfqllerycgyredsipqledvsrflkertgfqlrpvagllsardflaslafrvfqctqyirhasspmhsp 325
                                    acre l  f +        ed ipql+d+s +l   +g+++rpvagl+  rdfl  laf+ f+ tqy+rh s p ++p 
  tr|A0A0D2MT76|A0A0D2MT76_9CHLO 127 AACREFLHTFPM--------EDEIPQLQDISDILTATSGWKVRPVAGLMHPRDFLNGLAFKYFHSTQYVRHHSQPNYTP 197
                                     *********985........9********************************************************** PP

                      sequence 326 epdcchellghvpmladrtfaqfsqdiglaslgasdeeieklstvywftvefglckqngelkaygagllssygellhsl 404
                                   epd++hel+ghvpmlad  fa++ q ig+aslga ++ i +l+ vyw+tvefg++++ g +ka+gag+lssygel    
  tr|A0A0D2MT76|A0A0D2MT76_9CHLO 198 EPDVVHELIGHVPMLADPDFARMVQAIGVASLGAEEKTIWHLTKVYWYTVEFGVVREGGAVKAFGAGVLSSYGELDWMA 276
                                     ****************************************************************************999 PP

                      sequence 405 seepevrafdpdt 417
                                       e+  fdp +  
  tr|A0A0D2MT76|A0A0D2MT76_9CHLO 277 RGGAELAPFDPYA 289
                                     999******9965 PP

>> tr|A0A150G664|A0A150G664_GONPE  BH4_AAA_HYDROXYL_2 domain-containing protein OS=Gonium pectorale OX=33097 GN=GPECTOR_
   #    score  bias  c-Evalue  i-Evalue hmmfrom  hmm to    alifrom  ali to    envfrom  env to     acc
 ---   ------ ----- --------- --------- ------- -------    ------- -------    ------- -------    ----
   1 !  235.1   0.0   4.4e-72   6.9e-67     207     461 ..       2     252 ..       1     255 [. 0.97

  Alignments for each domain:
  == domain 1  score: 235.1 bits;  conditional E-value: 4.4e-72
```

```
                      sequence 207 iaeiafqykhgepiphveytaeeiatwkevyvtlkglyathacrehlegfqllerycgyredsipqledvsrflkertg 285
                                   +ae a q++ g  i  veyt ee+atw+ v   l+ l   hacre+l  + l++    +r   +pqle++  +l+  tg
  tr|A0A150G664|A0A150G664_GONPE   2 LAERAKQHQIGTAITDVEYTPEEVATWNAVLAQLRELLPRHACREYLRCLSLFD----FRPGKVPQLEEMNAVLQCSTG 76
                                   7999**********************************************9986....89999**************** PP

                      sequence 286 fqlrpvagllsardflaslafrvfqctqyirhasspmhspepdcchellghvpmladrtfaqfsqdiglaslgasdeei 364
                                   + +rpvagl+  r fla laf+ f+ tqy+rh s p ++pepd++hel+ghvp+lad +++q+ q ig aslga d+ i
  tr|A0A150G664|A0A150G664_GONPE  77 WTVRPVAGLMHPRHFLAGLAFKHFHSTQYMRHPSKPNYTPEPDVVHELIGHVPLLADPAYSQLVQAIGAASLGADDKTI 155
                                   ******************************************************************************* PP

                      sequence 365 eklstvywftvefglckqngelkaygagllssygellhslseepevrafdpdtaavqpyqdqtyqpvyfvsesfndakd 443
                                    +l+ vyw+tvefg++++ g +ka+gag+lssygel h  s    +  +dp     +    + yq  yfv +sf+
  tr|A0A150G664|A0A150G664_GONPE 156 WHLTKVYWYTVEFGVVREGGGIKAFGAGILSSYGELEHMASGAAALEPLDPFRPLPKMSYKDGYQSRYFVLDSFEAGAS 234
                                   ***************************************************998877766779**************** PP

                      sequence 444 klrnyasriqrpfsvkfd 461
                                    l+ ya+ +  p s++ d
  tr|A0A150G664|A0A150G664_GONPE 235 LLHSYAASLALPESLRGD 252
                                   ********9999988766 PP

>> tr|A0A2V0PJD4|A0A2V0PJD4_9CHLO  Chloroplast phenylalanine hydroxylase OS=Raphidocelis subcapitata OX=307507 GN=Rsub_1
   #    score  bias  c-Evalue  i-Evalue hmmfrom  hmm to    alifrom  ali to    envfrom  env to     acc
 ---   ------ ----- --------- --------- ------- -------    ------- -------    ------- -------    ----
   1 ?   -3.1   1.8       7.2   1.1e+06      48      69 ..      43      64 ..      28      75 .. 0.70
   2 !  211.2   0.0   8.1e-65   1.3e-59     168     450 ..     279     543 ..     255     549 .. 0.93

  Alignments for each domain:
  == domain 1  score: -3.1 bits;  conditional E-value: 7.2
                      sequence 48 ereaaaaaaaaavassepgnpl 69
                                    ea aaaaa a a   p  p
  tr|A0A2V0PJD4|A0A2V0PJD4_9CHLO 43 ASEAPAAAAARAAACPAPARPS 64
                                  3466677777777777777665 PP

  == domain 2  score: 211.2 bits;  conditional E-value: 8.1e-65
                      sequence 168 prkvseldkchhlvtkfdpdldldhpgfsdqvyrqrrkliaeiafqykhgepiphveytaeeiatwkevyvtlkglyat 246
                                   p  ++e+d+    +  f  dl  dhpg+ d+ y++rr  iae+a  +k gepip ++y+ ee+a w      l+ l+ t
  tr|A0A2V0PJD4|A0A2V0PJD4_9CHLO 279 PTSIAEVDNGS--ILGFGADLAPDHPGYHDEAYKRRRVAIAELARAHKIGEPIPRIDYSPEEVAVWGLALRELRRLFPT 355
                                   66777777765..45799************************************************************* PP

                      sequence 247 hacrehlegfqllerycgyredsipqledvsrflkertgfqlrpvagllsardflaslafrvfqctqyirhasspmhsp 325
                                   hac e    + ll+    +red ipql+d+s  l   +g+++rpvagl+  rdfl  laf+ f+ tq
  tr|A0A2V0PJD4|A0A2V0PJD4_9CHLO 356 HACAEFNHTLPLLD----FREDEIPQLQDISDALTAASGWKIRPVAGLMHPRDFLNGLAFKYFHSTQAR---------- 420
                                   *********99996....9*********************************************99953.......... PP

                      sequence 326 epdcchellghvpmladrtfaqfsqdiglaslgasdeeieklstvywftvefglckqngelkaygagllssygellhsl 404
                                          l+ghvpmlad  fa + q ig aslga +  i +l+  yw+tvefg++++ g +ka+gag+lssygel
```

```
  tr|A0A2V0PJD4|A0A2V0PJD4_9CHLO 421 --GGGAGLIGHVPMLADPDFAAMVQAIGRASLGADERTIWHLTKCYWYTVEFGVVREGGGVKAFGAGILSSYGELQWMA 497
                                     ..234579********************************************************************* PP

                        sequence 405 seepevrafdpdtaavqpyqdqtyqpvyfvsesfndakdklrnyas 450
                                     s   e+ afdp +   +    + yq  yf  +sf++    l+ ya+
  tr|A0A2V0PJD4|A0A2V0PJD4_9CHLO 498 SGAAELAAFDPYAPQPKMSYKDGYQRRYFALDSFQEGARLLQRYAA 543
                                     ***********988777666779*************9999999996 PP

>> tr|A0A061RS39|A0A061RS39_9CHLO  Tryptophan 5-monooxygenase OS=Tetraselmis sp. GSL018 OX=582737 GN=TPH PE=4 SV=1
   #    score  bias  c-Evalue  i-Evalue hmmfrom  hmm to    alifrom  ali to    envfrom  env to     acc
 ---   ------ ----- --------- --------- ------- -------    ------- -------    ------- -------    ----
   1 !  135.5   0.0   6.9e-42   1.1e-36     315     453 ..       1     139 [.       1     144 [. 0.95

  Alignments for each domain:
  == domain 1  score: 135.5 bits;  conditional E-value: 6.9e-42
                        sequence 315 irhasspmhspepdcchellghvpmladrtfaqfsqdiglaslgasdeeieklstvywftvefglckqngelkaygagl 393
                                     +rh s pm++pepd+ hellghv mlad  ++++ + ig asl asd+ei +l+ +yw+tvefg +k+  e++a+gagl
  tr|A0A061RS39|A0A061RS39_9CHLO   1 MRHHSEPMYTPEPDVIHELLGHVVMLADPVYCELVNTIGRASLAASDKEIWHLTKIYWYTVEFGTVKEGNEIRAFGAGL 79
                                     69***************************************************************************** PP

                        sequence 394 lssygellhslseepevrafdpdtaavqpyqdqtyqpvyfvsesfndakdklrnyasriq 453
                                     lssygel h  s    +    fdp +    +     + yq  yf+ +sf+d   +l+++a+ +
  tr|A0A061RS39|A0A061RS39_9CHLO  80 LSSYGELEHMRSGRAKFEPFDPFAKQPKMSYKDGYQERYFLMDSFEDGCRQLKEFAATMT 139
                                     **********************987766666679**********************98765 PP

>> tr|A0A2P6V6F8|A0A2P6V6F8_9CHLO  Chloroplast phenylalanine hydroxylase OS=Micractinium conductrix OX=554055 GN=C2E20_6
   #    score  bias  c-Evalue  i-Evalue hmmfrom  hmm to    alifrom  ali to    envfrom  env to     acc
 ---   ------ ----- --------- --------- ------- -------    ------- -------    ------- -------    ----
   1 !   93.1   0.0   4.9e-29   7.6e-24     334     455 ..      10     131 ..       6     138 .. 0.94

  Alignments for each domain:
  == domain 1  score: 93.1 bits;  conditional E-value: 4.9e-29
                        sequence 334 lghvpmladrtfaqfsqdiglaslgasdeeieklstvywftvefglckqngelkaygagllssygellhslseepevra 412
                                      gh+pmlad +fa +++ ig+asl a +e++++l  +yw+tvefg++++  ++ka+gag+lssygel h  +   ev
  tr|A0A2P6V6F8|A0A2P6V6F8_9CHLO  10 AGHLPMLADPSFASLAHAIGVASLAADEEQLKHLVKLYWYTVEFGVVREGSDVKAFGAGILSSYGELQHMAAGGAEVAP 88
                                     59***************************************************************************** PP

                        sequence 413 fdpdtaavqpyqdqtyqpvyfvsesfndakdklrnyasriqrp 455
                                     +d      +    + yq  yf  esf+     l+ y++ +q
  tr|A0A2P6V6F8|A0A2P6V6F8_9CHLO  89 LDVWQPLPKISYKDGYQKRYFALESFEAGAVELQAYCASLQAG 131
                                     **9887776666668*********************9999865 PP

>> tr|A0A6A0ACI4|A0A6A0ACI4_HAELA  Tryptophan 5-monooxygenase (Fragment) OS=Haematococcus lacustris OX=44745 GN=HaLaN_28
   #    score  bias  c-Evalue  i-Evalue hmmfrom  hmm to    alifrom  ali to    envfrom  env to     acc
 ---   ------ ----- --------- --------- ------- -------    ------- -------    ------- -------    ----
   1 !   22.9   0.0   9.4e-08     0.014     262     293 ..      10      41 ..       4      45 .. 0.90
```

```
  2 !   47.7   0.1     3e-15   4.6e-10     366     434 ..      42     110 ..      40     111 .] 0.91

 Alignments for each domain:
 == domain 1  score: 22.9 bits;  conditional E-value: 9.4e-08
                      sequence 262 ycgyredsipqledvsrflkertgfqlrpvag 293
                                    cg+r+d +pql+++s +l+ rtg+ +rpv
 tr|A0A6A0ACI4|A0A6A0ACI4_HAELA  10 ECGFRKDKVPQLQELSELLQRRTGWTIRPVIW 41
                                   5****************************975 PP

 == domain 2  score: 47.7 bits;  conditional E-value: 3e-15
                      sequence 366 klstvywftvefglckqngelkaygagllssygellhslseepevrafdpdtaavqpyqdqtyqpvyfv 434
                                   +l+ vyw+tvefg++++   +ka+gag+lss+gel h  +   ++  fdp +   +    + yq  yfv
 tr|A0A6A0ACI4|A0A6A0ACI4_HAELA  42 HLTKVYWYTVEFGVVREGDSVKAFGAGILSSFGELQHLAAGRAQLLPFDPFAPQPKMSYKDGYQQAYFV 110
                                   6899**********************************************987766666678*****98 PP

>> tr|A0A396H0S2|A0A396H0S2_MEDTR  DUF295 domain-containing protein OS=Medicago truncatula OX=3880 GN=MtrunA17_Chr7g0246
   #    score  bias  c-Evalue  i-Evalue hmmfrom  hmm to    alifrom  ali to    envfrom  env to     acc
 ---   ------ ----- --------- --------- ------- -------    ------- -------    ------- -------    ----
   1 ?   18.6   0.1   1.9e-06      0.29     425     487 ..     137     199 ..     118     208 .. 0.85

 Alignments for each domain:
 == domain 1  score: 18.6 bits;  conditional E-value: 1.9e-06
                      sequence 425 dqtyqpvyfvsesfndakdklrnyasriqrpfsvkfdpytlaidvldsphtiqrslegvqdel 487
                                    q y    f  +sfnd   k+r+ya   +rp s  +d y l +dv          le ++d++
 tr|A0A396H0S2|A0A396H0S2_MEDTR 137 VQNYMGNTFFVKSFNDDIWKVRKYAIDRDRPASYTLDVYKLELDVQSGKLEQMNKLESLEDNI 199
                                   477888888899*********************************988877778888888875 PP

>> tr|G7L2F2|G7L2F2_MEDTR  DUF295 family protein OS=Medicago truncatula OX=3880 GN=MTR_7g076100 PE=4 SV=1
   #    score  bias  c-Evalue  i-Evalue hmmfrom  hmm to    alifrom  ali to    envfrom  env to     acc
 ---   ------ ----- --------- --------- ------- -------    ------- -------    ------- -------    ----
   1 ?   18.4   0.1   2.3e-06      0.35     425     487 ..     167     229 ..     148     238 .. 0.85

 Alignments for each domain:
 == domain 1  score: 18.4 bits;  conditional E-value: 2.3e-06
              sequence 425 dqtyqpvyfvsesfndakdklrnyasriqrpfsvkfdpytlaidvldsphtiqrslegvqdel 487
                            q y    f  +sfnd   k+r+ya   +rp s  +d y l +dv          le ++d++
 tr|G7L2F2|G7L2F2_MEDTR 167 VQNYMGNTFFVKSFNDDIWKVRKYAIDRDRPASYTLDVYKLELDVQSGKLEQMNKLESLEDNI 229
                           477888888899*********************************988877778888888875 PP

>> tr|A2Q5C0|A2Q5C0_MEDTR  DUF295 domain-containing protein OS=Medicago truncatula OX=3880 GN=MtrDRAFT_AC160924g10v1 PE=
   #    score  bias  c-Evalue  i-Evalue hmmfrom  hmm to    alifrom  ali to    envfrom  env to     acc
 ---   ------ ----- --------- --------- ------- -------    ------- -------    ------- -------    ----
   1 ?   18.3   0.1   2.4e-06      0.37     425     487 ..     175     237 ..     156     246 .. 0.85

 Alignments for each domain:
 == domain 1  score: 18.3 bits;  conditional E-value: 2.4e-06
```

```
                 sequence 425 dqtyqpvyfvsesfndakdklrnyasriqrpfsvkfdpytlaidvldsphtiqrslegvqdel 487
                               q y    f  +sfnd   k+r+ya   +rp s  +d y l +dv          le ++d++
  tr|A2Q5C0|A2Q5C0_MEDTR 175 VQNYMGNTFFVKSFNDDIWKVRKYAIDRDRPASYTLDVYKLELDVQSGKLEQMNKLESLEDNI 237
                               467888888899*********************************988877778888888875 PP

>> tr|A0A199VCK3|A0A199VCK3_ANACO  AP2/ERF and B3 domain-containing transcription repressor TEM1 OS=Ananas comosus OX=46
   #    score  bias  c-Evalue  i-Evalue hmmfrom  hmm to    alifrom  ali to    envfrom  env to     acc
 ---   ------ ----- --------- --------- ------- -------    ------- -------    ------- -------    ----
   1 ?   -1.4   0.2       2.3   3.5e+05      33      61 ..     129     157 ..     118     165 .. 0.49
   2 ?   17.6   0.2   3.8e-06      0.58      11      72 ..     256     318 ..     248     323 .. 0.78

  Alignments for each domain:
  == domain 1  score: -1.4 bits;  conditional E-value: 2.3
                       sequence  33 rfigrrqsliedarkereaaaaaaaaava 61
                                    r    r+ l +  r    aaaaaaa   a
  tr|A0A199VCK3|A0A199VCK3_ANACO 129 RKHTYRDELRQSRRALGPAAAAAAAGQKA 157
                                    44444444444444444444444444444 PP

  == domain 2  score: 17.6 bits;  conditional E-value: 3.8e-06
                       sequence  11 pkgfrravseqdakqaeavtsprfigrrqsliedar.kereaaaaaaaaavassepgnpleav 72
                                     kg+ r v e+  k  + +t  r  g  + l  d + +   aaaaaaaaa    ep  p+  v
  tr|A0A199VCK3|A0A199VCK3_ANACO 256 TKGWSRFVKEKSLKAGDVITFHRSTGPEKQLFIDYKpRSASAAAAAAAAAAGPVEPDRPVRVV 318
                                    79*************************887776655145556666666667778999999876 PP

>> tr|A0A6A5NPF4|A0A6A5NPF4_LUPAL  Uncharacterized protein OS=Lupinus albus OX=3870 GN=Lal_00023996 PE=4 SV=1
   #    score  bias  c-Evalue  i-Evalue hmmfrom  hmm to    alifrom  ali to    envfrom  env to     acc
 ---   ------ ----- --------- --------- ------- -------    ------- -------    ------- -------    ----
   1 ?    7.6   0.0    0.0042   6.5e+02      64     123 ..     178     234 ..     170     275 .. 0.75
   2 ?    7.3   0.0     0.005   7.7e+02      83     154 ..     477     551 ..     462     567 .. 0.74

  Alignments for each domain:
  == domain 1  score: 7.6 bits;  conditional E-value: 0.0042
                       sequence  64 epgnpleavvfeerdgnavlnllfslrgtkpsslsravkvfetfeakihhletrpaqrpl 123
                                    epg+   a+ +++ d     nllfs + +  s  s  vkv + fea++  +e   a   l
  tr|A0A6A5NPF4|A0A6A5NPF4_LUPAL 178 EPGQKSRAMGIKDEDQ---RNLLFSEKEASWSRYSEQVKVGDIFEARVSSIEDYGAFVDL 234
                                    6777777776666664...59****************************99988776544 PP

  == domain 2  score: 7.3 bits;  conditional E-value: 0.005
                       sequence  83 lnllfslrgtkpsslsravkvfetfeakihhletrpaqrpl...agsphleyfvrfevpsgdlaallssvrrvsd 154
                                      l+fs + +  s  s+ vkv + feak+  +e   a   l    g  hl  ++++   s dl   +  +    d
  tr|A0A6A5NPF4|A0A6A5NPF4_LUPAL 477 QSLMFSEKEASWSRYSKQVKVGDIFEAKVSCIEEYGAFVDLrfpDGLYHLHGLIHISEMSWDLVDNVRDILTECD 551
                                    4689*****************************999887555222577788888887777777766555555444 PP

>> tr|A0A061SG95|A0A061SG95_9CHLO  Uncharacterized protein (Fragment) OS=Tetraselmis sp. GSL018 OX=582737 GN=TSPGSL018_1
   #    score  bias  c-Evalue  i-Evalue hmmfrom  hmm to    alifrom  ali to    envfrom  env to     acc
 ---   ------ ----- --------- --------- ------- -------    ------- -------    ------- -------    ----
```

```
   1 ?   16.2   0.6     1e-05       1.6      43     155 ..      89     206 ..      66     213 .. 0.75

  Alignments for each domain:
  == domain 1  score: 16.2 bits;  conditional E-value: 1e-05
                         sequence  43 edar.kereaaaaa..aaaav..assepgnpleav..vfeerdgnavlnllfslrgtkpsslsravkvfetfeakihhl 114
                                      + ar  ereaa a    aa v  +  +  + +ea    fee+    v      + gtk +     v+v+e+ +  i+ l
  tr|A0A061SG95|A0A061SG95_9CHLO  89 KAARaAEREAAEAMetEAAEVgmTVEQYQKAVEAQqqHFEEKRKAKVAGAAADMDGTKRAFYKEFVRVVEASDVVIQVL 167
                                      44442467776653112232310333445566664227**************************************999 PP

                         sequence 115 etrpaqrplag.sphleyfvrfevpsgdlaallssvrrvsdd 155
                                      ++r    pla   p +e fvr   p   +  ll  v  v  d
  tr|A0A061SG95|A0A061SG95_9CHLO 168 DAR---DPLACrCPDVERFVRRMNPNKKVVLLLNKVDLVPRD 206
                                      876...6888637********************999887544 PP

>> tr|A0A397Y5H5|A0A397Y5H5_BRACM  Uncharacterized protein (Fragment) OS=Brassica campestris OX=3711 GN=BRARA_I05337 PE=
   #    score  bias  c-Evalue  i-Evalue hmmfrom  hmm to    alifrom  ali to    envfrom  env to     acc
 ---   ------ ----- --------- --------- ------- -------    ------- -------    ------- -------    ----
   1 ?   16.3   0.4   9.8e-06       1.5      32      77 ..       4      49 ..       2      72 .. 0.66

  Alignments for each domain:
  == domain 1  score: 16.3 bits;  conditional E-value: 9.8e-06
                         sequence 32 prfigrrqsli.edarkereaaaaaaaaavassepgnpleavvfeer 77
                                      rfigrr   +   ar e  +aaaaaaa  ++    nple     +r
  tr|A0A397Y5H5|A0A397Y5H5_BRACM  4 TRFIGRRFLAVaASARSESTTAAAAAAA-STARIAKNPLEEFFEFDR 49
                                     59*****865515677776666655555.55567889*997643344 PP

>> tr|A0A6A4PI42|A0A6A4PI42_LUPAL  Putative ribosomal protein S1 OS=Lupinus albus OX=3870 GN=Lalb_Chr13g0294601 PE=4 SV=
   #    score  bias  c-Evalue  i-Evalue hmmfrom  hmm to    alifrom  ali to    envfrom  env to     acc
 ---   ------ ----- --------- --------- ------- -------    ------- -------    ------- -------    ----
   1 ?    6.5   0.0    0.0089   1.4e+03      83     123 ..     165     205 ..     151     248 .. 0.76
   2 ?    7.5   0.0    0.0046   7.1e+02      83     154 ..     437     511 ..     422     527 .. 0.74

  Alignments for each domain:
  == domain 1  score: 6.5 bits;  conditional E-value: 0.0089
                         sequence  83 lnllfslrgtkpsslsravkvfetfeakihhletrpaqrpl 123
                                       nllfs + +  s  s  vkv + fea++  +e   a   l
  tr|A0A6A4PI42|A0A6A4PI42_LUPAL 165 RNLLFSEKEASWSRYSEQVKVGDIFEARVSSIEDYGAFVDL 205
                                      69****************************99988776544 PP

  == domain 2  score: 7.5 bits;  conditional E-value: 0.0046
                         sequence  83 lnllfslrgtkpsslsravkvfetfeakihhletrpaqrpl...agsphleyfvrfevpsgdlaallssvrrvsd 154
                                        l+fs + +  s  s+ vkv + feak+  +e   a   l    g  hl  ++++   s dl   +  +    d
  tr|A0A6A4PI42|A0A6A4PI42_LUPAL 437 QSLMFSEKEASWSRYSKQVKVGDIFEAKVSCIEEYGAFVDLrfpDGLYHLHGLIHISEMSWDLVDNVRDILTECD 511
                                      4689****************************9998875552225777888888777777776655555555444 PP

>> tr|A0A2Z6ZTF1|A0A2Z6ZTF1_9LAMI  Uncharacterized protein OS=Dorcoceras hygrometricum OX=472368 GN=F511_46696 PE=4 SV=1
```

```
   #    score  bias  c-Evalue  i-Evalue hmmfrom  hmm to    alifrom  ali to    envfrom  env to     acc
 ---   ------ ----- --------- --------- ------- -------    ------- -------    ------- -------    ----
   1 ?   14.5   5.2   3.3e-05       5.1       5      57 ..      78     129 ..      75     134 .. 0.83

  Alignments for each domain:
  == domain 1  score: 14.5 bits;  conditional E-value: 3.3e-05
                      sequence   5 sapspqpkgfrravseqdakqaeavtsprfigrrqsliedarkereaaaaaaa 57
                                   s p p+p+ +r+a  e  ++ a    spr igr+q +    r+ r aaaaaaa
  tr|A0A2Z6ZTF1|A0A2Z6ZTF1_9LAMI  78 SGPRPDPRLLRQAALEAMTRSAR-TDSPRRIGRKQFFRRIRRRRVAAAAAAA 129
                                   889***********99988886.589********988777777777776665 PP

>> tr|I0YL19|I0YL19_COCSC  Uncharacterized protein OS=Coccomyxa subellipsoidea (strain C-169) OX=574566 GN=COCSUDRAFT_44
   #    score  bias  c-Evalue  i-Evalue hmmfrom  hmm to    alifrom  ali to    envfrom  env to     acc
 ---   ------ ----- --------- --------- ------- -------    ------- -------    ------- -------    ----
   1 ?   14.2   0.9   4.3e-05       6.6      39     111 ..     587     660 ..     576     669 .. 0.68

  Alignments for each domain:
  == domain 1  score: 14.2 bits;  conditional E-value: 4.3e-05
              sequence  39 qsliedarkereaaaaaaaaavassepgnpleavvfeerdgnavlnllfs..lrgtkpsslsravkvfetfeaki 111
                           +s  +   ke eaaaaaaaaa as+      e  v+   dg av ++     +   +p+sl     +   f+ ki
  tr|I0YL19|I0YL19_COCSC 587 RSKKKSKAKEEEAAAAAAAAA-ASAAAAANTEVKVYRGEDGGAVKEVCSNviMGSDRPTSLHGGAVLGVGFDKKI 660
                           44445556777777666655.6666777789***********988754113445788887655555556655 PP

>> tr|A0A059CY94|A0A059CY94_EUCGR  Uncharacterized protein OS=Eucalyptus grandis OX=71139 GN=EUGRSUZ_B00348 PE=4 SV=1
   #    score  bias  c-Evalue  i-Evalue hmmfrom  hmm to    alifrom  ali to    envfrom  env to     acc
 ---   ------ ----- --------- --------- ------- -------    ------- -------    ------- -------    ----
   1 ?   14.4   0.2   3.5e-05       5.4      32      78 ..      26      70 ..      17      83 .. 0.84

  Alignments for each domain:
  == domain 1  score: 14.4 bits;  conditional E-value: 3.5e-05
                      sequence 32 prfigrrqsliedarkereaaaaaaaaavassepgnpleavvfeerd 78
                                   rf gr  +l   ar e  a +aaaa+a +s    nplea    +r+
  tr|A0A059CY94|A0A059CY94_EUCGR 26 SRFFGR--ALFAAARTESSAGTAAAASAATSRTGHNPLEAFFEADRN 70
                                  688887..6999**************99999999****97655554 PP

>> tr|A0A5D2SK86|A0A5D2SK86_GOSMU  Uncharacterized protein OS=Gossypium mustelinum OX=34275 GN=E1A91_D12G313000v1 PE=4 S
   #    score  bias  c-Evalue  i-Evalue hmmfrom  hmm to    alifrom  ali to    envfrom  env to     acc
 ---   ------ ----- --------- --------- ------- -------    ------- -------    ------- -------    ----
   1 ?   14.3   0.6   3.9e-05         6      32      77 ..       4      48 ..       2      59 .. 0.87

  Alignments for each domain:
  == domain 1  score: 14.3 bits;  conditional E-value: 3.9e-05
                      sequence 32 prfigrrqsliedarkereaaaaaaaaavassepgnpleavvfeer 77
                                   rf+gr  +l+  a+ e  aa++aaaa+ a+    nple +   +r
  tr|A0A5D2SK86|A0A5D2SK86_GOSMU  4 SRFVGR-TALLAAAKSESSAATTAAAASAAAPTNLNPLEQIFEADR 48
                                  599997.579999*****************9*******9865555 PP
```

```
>> tr|A0A059CZ03|A0A059CZ03_EUCGR  Uncharacterized protein OS=Eucalyptus grandis OX=71139 GN=EUGRSUZ_B00348 PE=4 SV=1
   #    score  bias  c-Evalue  i-Evalue hmmfrom  hmm to    alifrom  ali to    envfrom  env to     acc
 ---   ------ ----- --------- --------- ------- -------    ------- -------    ------- -------    ----
   1 ?   14.2   0.2   4.2e-05       6.5      33      78 ..       5      48 ..       2      62 .. 0.85

  Alignments for each domain:
  == domain 1  score: 14.2 bits;  conditional E-value: 4.2e-05
                        sequence 33 rfigrrqsliedarkereaaaaaaaaavassepgnpleavvfeerd 78
                                    rf gr  +l   ar e  a +aaaa+a +s    nplea    +r+
  tr|A0A059CZ03|A0A059CZ03_EUCGR  5 RFFGR--ALFAAARTESSAGTAAAASAATSRTGHNPLEAFFEADRN 48
                                    78887..69999*************999999999****97655554 PP

>> tr|B9RTY8|B9RTY8_RICCO  Protein binding protein, putative OS=Ricinus communis OX=3988 GN=RCOM_0913950 PE=4 SV=1
   #    score  bias  c-Evalue  i-Evalue hmmfrom  hmm to    alifrom  ali to    envfrom  env to     acc
 ---   ------ ----- --------- --------- ------- -------    ------- -------    ------- -------    ----
   1 ?   13.7   1.0   5.9e-05       9.1      32     108 ..      14      91 ..       2     100 .. 0.78

  Alignments for each domain:
  == domain 1  score: 13.7 bits;  conditional E-value: 5.9e-05
                sequence  32 prfigrrqsliedarker.eaaaaaaaaavassepgnpleavvfeerdgnavlnllfslrgtkpsslsravkvfetfe 108
                             p   g+++ l e+   e+ e +a+a+aaa a+   g+  +a+++e      +ln  fs   +  ++  ra++v+  f
  tr|B9RTY8|B9RTY8_RICCO  14 PERKGQKRKLEEEIEDEQqEISASATAAAAAAVPFGDARQALLYEVASQVNILNSTFSWNEADRAAAKRAIHVLAEFA 91
                             66667777787777666514555556666667779***************************************98884 PP

>> tr|I0YKX2|I0YKX2_COCSC  BZIP domain-containing protein OS=Coccomyxa subellipsoidea (strain C-169) OX=574566 GN=COCSUD
   #    score  bias  c-Evalue  i-Evalue hmmfrom  hmm to    alifrom  ali to    envfrom  env to     acc
 ---   ------ ----- --------- --------- ------- -------    ------- -------    ------- -------    ----
   1 ?   13.7   0.1   5.9e-05       9.1      33     136 ..     259     366 ..     225     397 .. 0.69

  Alignments for each domain:
  == domain 1  score: 13.7 bits;  conditional E-value: 5.9e-05
                sequence  33 rfigrrqsliedarker....eaaaaaaaaavassepgnpleavvfeerdgnavlnllfslrgtkpsslsravkvfetfeakihhle 115
                              +  r qsl  + r+ +     a++a+ aaa    e   p +av +  + g+  ++l +s+   k  +l r v++++t+  ki +
  tr|I0YKX2|I0YKX2_COCSC 259 ELSERVQSLELEKRQLQmaleRASTATKAAAAQNGEQKSPDDAVAIALKLGEPTVQLQLSVGDVKVMTLERMVHIWKTMVHKIGQYL 345
                             3445677776666654311112445555666778899 9************************************************98877 PP

                sequence 116 trpaqrplagsphleyfvrfe 136
                                 q p     h    + fe
  tr|I0YKX2|I0YKX2_COCSC 346 PEAEQSPGGLKFHCLEDMAFE 366
                             777777765555554455555 PP

>> tr|A0A2P5EQI7|A0A2P5EQI7_TREOI  Uncharacterized protein OS=Trema orientale OX=63057 GN=TorRG33x02_164400 PE=4 SV=1
   #    score  bias  c-Evalue  i-Evalue hmmfrom  hmm to    alifrom  ali to    envfrom  env to     acc
 ---   ------ ----- --------- --------- ------- -------    ------- -------    ------- -------    ----
   1 ?   14.0   0.1   4.7e-05       7.2     307     360 ..      61     115 ..      50     121 .. 0.89
```

```
  Alignments for each domain:
  == domain 1  score: 14.0 bits;  conditional E-value: 4.7e-05
                      sequence 307 rvfqctqyirhasspmhsp.epdcchellghvpmladrtfaqfsqdiglaslgas 360
                                    +f+ tq++   s p+  p +p   h+ l  +  la +t+ qfs+  g++s  +s
  tr|A0A2P5EQI7|A0A2P5EQI7_TREOI  61 ALFNTTQHLSALSHPILGPsQPTSQHKSLATICSLASKTLVQFSKTGGITSSKSS 115
                                   5799***********999846999************************9997766 PP

>> tr|A0A2P6TE94|A0A2P6TE94_CHLSO  SufE chloroplastic OS=Chlorella sorokiniana OX=3076 GN=C2E21_8555 PE=3 SV=1
   #    score  bias  c-Evalue  i-Evalue hmmfrom  hmm to    alifrom  ali to    envfrom  env to     acc
 ---   ------ ----- --------- --------- ------- -------    ------- -------    ------- -------    ----
   1 ?   14.2   0.7   4.1e-05       6.3      47     125 ..      29     107 ..       2     131 .. 0.80

  Alignments for each domain:
  == domain 1  score: 14.2 bits;  conditional E-value: 4.1e-05
                      sequence  47 kereaaaaaaaaavassepgnpleavvfeerdgnavlnllfslrgtkpsslsravkvfetfeakihhletrpaqrplag 125
                                     r + aaaa aa a+  pg+ l a      d n   +l+  +rg   ++l   +  +e ++    h+e     + + g
  tr|A0A2P6TE94|A0A2P6TE94_CHLSO  29 AGRPSLAAAAPAAAAAVRPGQLLAARASSSMDNNISSQLMDQMRGKIQAALNAEIVQVEDMQGDGRHVEIMVVSKEFEG 107
                                   3455557888899999**********************************99999999999999999998777666655 PP

>> tr|A0A699ZJV5|A0A699ZJV5_HAELA  TAF domain-containing protein OS=Haematococcus lacustris OX=44745 GN=HaLaN_19742 PE=4
   #    score  bias  c-Evalue  i-Evalue hmmfrom  hmm to    alifrom  ali to    envfrom  env to     acc
 ---   ------ ----- --------- --------- ------- -------    ------- -------    ------- -------    ----
   1 ?   13.6   1.2   6.3e-05       9.6      34      84 ..      83     133 ..      74     138 .. 0.84

  Alignments for each domain:
  == domain 1  score: 13.6 bits;  conditional E-value: 6.3e-05
                      sequence  34 figrrqsliedarkereaaaaaaaaavassepgnpleavvfeerdgnavln 84
                                   fi   q       k  +aa+aaaaaava  e g+ l av   e +  avl
  tr|A0A699ZJV5|A0A699ZJV5_HAELA  83 FIEGLQLYFAKVAKVVNAATAAAAAAVAGQEAGQGLAAVQAVEGQLRAVLA 133
                                   55556666677788999**********************999988888885 PP

>> tr|I0ZAJ8|I0ZAJ8_COCSC  Uncharacterized protein OS=Coccomyxa subellipsoidea (strain C-169) OX=574566 GN=COCSUDRAFT_55
   #    score  bias  c-Evalue  i-Evalue hmmfrom  hmm to    alifrom  ali to    envfrom  env to     acc
 ---   ------ ----- --------- --------- ------- -------    ------- -------    ------- -------    ----
   1 ?   12.9   0.9   0.00011        16      18      64 ..     390     434 ..     383     455 .. 0.81

  Alignments for each domain:
  == domain 1  score: 12.9 bits;  conditional E-value: 0.00011
              sequence  18 vseqdakqaeavtsprfigrrqsliedarkereaaaaaaaaavasse 64
                           +s+qd+ q +     r++ r+ sl+e+a   + aaaa aaaa
  tr|I0ZAJ8|I0ZAJ8_COCSC 390 LSQQDTFQQQVADLHRLVKRQTSLMEEAAAAQAAAAAVAAAAA--GA 434
                           799************************9887766665555443..23 PP

>> tr|A0A2I0AQ32|A0A2I0AQ32_9ASPA  40S ribosomal protein S4 OS=Apostasia shenzhenica OX=1088818 GN=RPS4 PE=3 SV=1
   #    score  bias  c-Evalue  i-Evalue hmmfrom  hmm to    alifrom  ali to    envfrom  env to     acc
```

```
 ---   ------ ----- --------- --------- ------- -------    ------- -------    ------- -------    ----
   1 ?   13.4   1.5   7.2e-05        11      29      61 ..     236     268 .]     229     268 .] 0.86

  Alignments for each domain:
  == domain 1  score: 13.4 bits;  conditional E-value: 7.2e-05
                       sequence  29 vtsprfigrrqsliedarkereaaaaaaaaava 61
                                    vt p+  g + s+ie+ark   aaaaaaaaa a
  tr|A0A2I0AQ32|A0A2I0AQ32_9ASPA 236 VTLPKGKGIKLSIIEEARKRAAAAAAAAAAATA 268
                                    8999999************99998888888865 PP

>> tr|A0A5D2IGR4|A0A5D2IGR4_GOSTO  Uncharacterized protein OS=Gossypium tomentosum OX=34277 GN=ES332_D12G322800v1 PE=4 S
   #    score  bias  c-Evalue  i-Evalue hmmfrom  hmm to    alifrom  ali to    envfrom  env to     acc
 ---   ------ ----- --------- --------- ------- -------    ------- -------    ------- -------    ----
   1 ?   13.5   0.4   6.8e-05        10      32      77 ..       4      48 ..       2      66 .. 0.86

  Alignments for each domain:
  == domain 1  score: 13.5 bits;  conditional E-value: 6.8e-05
                       sequence 32 prfigrrqsliedarkereaaaaaaaaavassepgnpleavvfeer 77
                                    rf+gr  +l+  a+ e  aa++aaaa+ a+    nple     +r
  tr|A0A5D2IGR4|A0A5D2IGR4_GOSTO  4 SRFVGR-TALLAAAKSESSAATTAAAASAAAPTNLNPLEQFFEADR 48
                                   599997.579999**************999999******8654444 PP

>> tr|A0A2R6X400|A0A2R6X400_MARPO  Uncharacterized protein OS=Marchantia polymorpha OX=3197 GN=MARPO_0037s0016 PE=4 SV=1
   #    score  bias  c-Evalue  i-Evalue hmmfrom  hmm to    alifrom  ali to    envfrom  env to     acc
 ---   ------ ----- --------- --------- ------- -------    ------- -------    ------- -------    ----
   1 ?   13.4   1.0   7.4e-05        11      13     107 ..      34     130 ..      32     150 .. 0.83

  Alignments for each domain:
  == domain 1  score: 13.4 bits;  conditional E-value: 7.4e-05
                       sequence  13 gfrravseqdakqaeavtsprfigrrqsliedarkereaaaaaaaaavasse.pgnpleavvfeerdgnavlnllfsl. 89
                                     f+r  se+  k+ae  +    + + q  +  ++k   aaa a aaav  +  p   l+ v + +  g+ v ++ f+
  tr|A0A2R6X400|A0A2R6X400_MARPO  34 TFKRQKSEKLKKEAEEFAKANAVQQPQPAVHASKKSATAAAKAKAAAVEKAHaPRHHLDLVTL-KGHGDVVTDVAFTSn 111
                                    599****************************************99999876527777777766.567999999999762 PP

                       sequence  90 .rgtkpsslsravkvfetf 107
                                     rg   +   + v+vf+
  tr|A0A2R6X400|A0A2R6X400_MARPO 112 gRGLATACADQVVRVFKLE 130
                                    3667777888888888754 PP

>> tr|A0A0B0NP41|A0A0B0NP41_GOSAR  39S ribosomal L47, mitochondrial OS=Gossypium arboreum OX=29729 GN=F383_07486 PE=4 SV
   #    score  bias  c-Evalue  i-Evalue hmmfrom  hmm to    alifrom  ali to    envfrom  env to     acc
 ---   ------ ----- --------- --------- ------- -------    ------- -------    ------- -------    ----
   1 ?   13.5   0.4   6.8e-05        10      32      77 ..       4      48 ..       2      66 .. 0.86

  Alignments for each domain:
  == domain 1  score: 13.5 bits;  conditional E-value: 6.8e-05
```

```
                        sequence 32 prfigrrqsliedarkereaaaaaaaaaavassepgnpleavvfeer 77
                                     rf+gr  +l+  a+ e  aa++aaaa+ a+    nple     +r
  tr|A0A0B0NP41|A0A0B0NP41_GOSAR  4 SRFVGR-TALLAAAKSESSAATTAAAASAAAPTNLNPLEQFFEADR 48
                                    599997.579999**************999999******8654444 PP

>> tr|A0A1U8MSW2|A0A1U8MSW2_GOSHI  39S ribosomal protein L47, mitochondrial-like OS=Gossypium hirsutum OX=3635 GN=LOC107
   #    score  bias  c-Evalue  i-Evalue hmmfrom  hmm to    alifrom  ali to    envfrom  env to     acc
 ---   ------ ----- --------- --------- ------- -------    ------- -------    ------- -------    ----
   1 ?   13.5   0.4   6.8e-05        10      32      77 ..       4      48 ..       2      66 .. 0.86

  Alignments for each domain:
  == domain 1  score: 13.5 bits;  conditional E-value: 6.8e-05
                        sequence 32 prfigrrqsliedarkereaaaaaaaaaavassepgnpleavvfeer 77
                                     rf+gr  +l+  a+ e  aa++aaaa+ a+    nple     +r
  tr|A0A1U8MSW2|A0A1U8MSW2_GOSHI  4 SRFVGR-TALLAAAKSESSAATTAAAASAAAPTNLNPLEQFFEADR 48
                                    599997.579999**************999999******8654444 PP

>> tr|A0A0D2T8Q2|A0A0D2T8Q2_GOSRA  Uncharacterized protein OS=Gossypium raimondii OX=29730 GN=B456_008G297400 PE=4 SV=1
   #    score  bias  c-Evalue  i-Evalue hmmfrom  hmm to    alifrom  ali to    envfrom  env to     acc
 ---   ------ ----- --------- --------- ------- -------    ------- -------    ------- -------    ----
   1 ?   13.5   0.4   6.8e-05        10      32      77 ..       4      48 ..       2      66 .. 0.86

  Alignments for each domain:
  == domain 1  score: 13.5 bits;  conditional E-value: 6.8e-05
                        sequence 32 prfigrrqsliedarkereaaaaaaaaaavassepgnpleavvfeer 77
                                     rf+gr  +l+  a+ e  aa++aaaa+ a+    nple     +r
  tr|A0A0D2T8Q2|A0A0D2T8Q2_GOSRA  4 SRFVGR-TALLAAAKSESSAATTAAAASAAAPTNLNPLEQFFEADR 48
                                    599997.579999**************999999******8654444 PP

>> tr|A0A1U8MRB8|A0A1U8MRB8_GOSHI  39S ribosomal protein L47, mitochondrial-like OS=Gossypium hirsutum OX=3635 GN=LOC107
   #    score  bias  c-Evalue  i-Evalue hmmfrom  hmm to    alifrom  ali to    envfrom  env to     acc
 ---   ------ ----- --------- --------- ------- -------    ------- -------    ------- -------    ----
   1 ?   13.5   0.4   6.8e-05        10      32      77 ..       4      48 ..       2      66 .. 0.86

  Alignments for each domain:
  == domain 1  score: 13.5 bits;  conditional E-value: 6.8e-05
                        sequence 32 prfigrrqsliedarkereaaaaaaaaaavassepgnpleavvfeer 77
                                     rf+gr  +l+  a+ e  aa++aaaa+ a+    nple     +r
  tr|A0A1U8MRB8|A0A1U8MRB8_GOSHI  4 SRFVGR-TALLAAAKSESSAATTAAAASAAAPTNLNPLEQFFEADR 48
                                    599997.579999**************999999******8654444 PP

>> tr|A0A5D2EGM7|A0A5D2EGM7_GOSDA  Uncharacterized protein OS=Gossypium darwinii OX=34276 GN=ES288_A12G312000v1 PE=4 SV=
   #    score  bias  c-Evalue  i-Evalue hmmfrom  hmm to    alifrom  ali to    envfrom  env to     acc
 ---   ------ ----- --------- --------- ------- -------    ------- -------    ------- -------    ----
   1 ?   13.5   0.4   6.8e-05        10      32      77 ..       4      48 ..       2      66 .. 0.86
```

```
  Alignments for each domain:
  == domain 1  score: 13.5 bits;  conditional E-value: 6.8e-05
                      sequence 32 prfigrrqsliedarkereaaaaaaaaavassepgnpleavvfeer 77
                                  rf+gr  +l+  a+ e  aa++aaaa+ a+    nple     +r
  tr|A0A5D2EGM7|A0A5D2EGM7_GOSDA  4 SRFVGR-TALLAAAKSESSAATTAAAASAAAPTNLNPLEQFFEADR 48
                                  599997.579999**************999999******8654444 PP

>> tr|A0A5D2N494|A0A5D2N494_GOSTO  Uncharacterized protein OS=Gossypium tomentosum OX=34277 GN=ES332_A12G312800v1 PE=4 S
   #    score  bias  c-Evalue  i-Evalue hmmfrom  hmm to    alifrom  ali to    envfrom  env to     acc
 ---   ------ ----- --------- --------- ------- -------    ------- -------    ------- -------    ----
   1 ?   13.5   0.4   6.8e-05        10      32      77 ..       4      48 ..       2      66 .. 0.86

  Alignments for each domain:
  == domain 1  score: 13.5 bits;  conditional E-value: 6.8e-05
                      sequence 32 prfigrrqsliedarkereaaaaaaaaavassepgnpleavvfeer 77
                                  rf+gr  +l+  a+ e  aa++aaaa+ a+    nple     +r
  tr|A0A5D2N494|A0A5D2N494_GOSTO  4 SRFVGR-TALLAAAKSESSAATTAAAASAAAPTNLNPLEQFFEADR 48
                                  599997.579999**************999999******8654444 PP

>> tr|A0A5D2X3A8|A0A5D2X3A8_GOSMU  Uncharacterized protein OS=Gossypium mustelinum OX=34275 GN=E1A91_A12G300700v1 PE=4 S
   #    score  bias  c-Evalue  i-Evalue hmmfrom  hmm to    alifrom  ali to    envfrom  env to     acc
 ---   ------ ----- --------- --------- ------- -------    ------- -------    ------- -------    ----
   1 ?   13.5   0.4   6.8e-05        10      32      77 ..       4      48 ..       2      66 .. 0.86

  Alignments for each domain:
  == domain 1  score: 13.5 bits;  conditional E-value: 6.8e-05
                      sequence 32 prfigrrqsliedarkereaaaaaaaaavassepgnpleavvfeer 77
                                  rf+gr  +l+  a+ e  aa++aaaa+ a+    nple     +r
  tr|A0A5D2X3A8|A0A5D2X3A8_GOSMU  4 SRFVGR-TALLAAAKSESSAATTAAAASAAAPTNLNPLEQFFEADR 48
                                  599997.579999**************999999******8654444 PP

>> tr|A0A5J5TKL3|A0A5J5TKL3_GOSBA  Uncharacterized protein OS=Gossypium barbadense OX=3634 GN=ES319_A12G285900v1 PE=4 SV
   #    score  bias  c-Evalue  i-Evalue hmmfrom  hmm to    alifrom  ali to    envfrom  env to     acc
 ---   ------ ----- --------- --------- ------- -------    ------- -------    ------- -------    ----
   1 ?   13.5   0.4   6.8e-05        10      32      77 ..       4      48 ..       2      66 .. 0.86

  Alignments for each domain:
  == domain 1  score: 13.5 bits;  conditional E-value: 6.8e-05
                      sequence 32 prfigrrqsliedarkereaaaaaaaaavassepgnpleavvfeer 77
                                  rf+gr  +l+  a+ e  aa++aaaa+ a+    nple     +r
  tr|A0A5J5TKL3|A0A5J5TKL3_GOSBA  4 SRFVGR-TALLAAAKSESSAATTAAAASAAAPTNLNPLEQFFEADR 48
                                  599997.579999**************999999******8654444 PP

>> tr|A0A0D2MIV3|A0A0D2MIV3_9CHLO  NFACT-R_1 domain-containing protein OS=Monoraphidium neglectum OX=145388 GN=MNEG_1302
   #    score  bias  c-Evalue  i-Evalue hmmfrom  hmm to    alifrom  ali to    envfrom  env to     acc
 ---   ------ ----- --------- --------- ------- -------    ------- -------    ------- -------    ----
```

```
   1 ?   13.2   1.0   8.2e-05        13      19      90 ..     249     321 ..     235     351 .. 0.83

  Alignments for each domain:
  == domain 1  score: 13.2 bits;  conditional E-value: 8.2e-05
                      sequence  19 seqdakqaeavtsprfigrrqsliedarkereaaaaaaaaavassepgnpleavvfeer.dgnavlnllfslr 90
                                     +q a+ a   +       r +lie    e +aa  a  aavas      l+a++ eer  gn v  l+ sl+
  tr|A0A0D2MIV3|A0A0D2MIV3_9CHLO 249 QDQGARVAALESEASEAELRATLIEYNLGEVDAAIDAVNAAVASGMDWRELDAMIREERrAGNPVAGLIHSLQ 321
                                     4555555554455555667999*********************************9986379*******9996 PP

>> tr|A0A1D6FM46|A0A1D6FM46_MAIZE  Protein apaG OS=Zea mays OX=4577 GN=ZEAMMB73_Zm00001d009800 PE=4 SV=1
   #    score  bias  c-Evalue  i-Evalue hmmfrom  hmm to    alifrom  ali to    envfrom  env to     acc
 ---   ------ ----- --------- --------- ------- -------    ------- -------    ------- -------    ----
   1 ?   13.3   0.6   7.9e-05        12      38     111 ..      28     101 ..       8     117 .. 0.83

  Alignments for each domain:
  == domain 1  score: 13.3 bits;  conditional E-value: 7.9e-05
                      sequence  38 rqsliedarkereaaaaaaaaavassepgnpl..eavvfeerdgnavlnllfslrgtkpsslsravkvfetfeaki 111
                                    q     ar+   a+aaa+ a  +s  pg+    e vv  e++ +av  ll  +r  k + l + + v   feak
  tr|A0A1D6FM46|A0A1D6FM46_MAIZE  28 PQGRFPTARRALVASAAASDANSSSNSPGKDEegEEVVRREKEEKAVASLL--MRSQKYAMLKQQLAVAAQFEAKA 101
                                     4667778999999999999999999**8752268*************99..5999*****************96 PP

>> tr|A0A3L6DRF5|A0A3L6DRF5_MAIZE  Protein ApaG OS=Zea mays OX=4577 GN=apaG_1 PE=4 SV=1
   #    score  bias  c-Evalue  i-Evalue hmmfrom  hmm to    alifrom  ali to    envfrom  env to     acc
 ---   ------ ----- --------- --------- ------- -------    ------- -------    ------- -------    ----
   1 ?   13.3   0.6   7.9e-05        12      38     111 ..      28     101 ..       8     117 .. 0.83

  Alignments for each domain:
  == domain 1  score: 13.3 bits;  conditional E-value: 7.9e-05
                      sequence  38 rqsliedarkereaaaaaaaaavassepgnpl..eavvfeerdgnavlnllfslrgtkpsslsravkvfetfeaki 111
                                    q     ar+   a+aaa+ a  +s  pg+    e vv  e++ +av  ll  +r  k + l + + v   feak
  tr|A0A3L6DRF5|A0A3L6DRF5_MAIZE  28 PQGRFPTARRALVASAAASDANSSSNSPGKDEegEEVVRREKEEKAVASLL--MRSQKYAMLKQQLAVAAQFEAKA 101
                                     4667778999999999999999999**8752268*************99..5999*****************96 PP

>> tr|A0A2Z6ZTG8|A0A2Z6ZTG8_9LAMI  Uncharacterized protein OS=Dorcoceras hygrometricum OX=472368 GN=F511_46884 PE=4 SV=1
   #    score  bias  c-Evalue  i-Evalue hmmfrom  hmm to    alifrom  ali to    envfrom  env to     acc
 ---   ------ ----- --------- --------- ------- -------    ------- -------    ------- -------    ----
   1 ?   13.6   2.9   6.2e-05       9.5       5      59 ..      38      91 ..      34      92 .] 0.85

  Alignments for each domain:
  == domain 1  score: 13.6 bits;  conditional E-value: 6.2e-05
                      sequence  5 sapspqpkgfrravseqdakqaeavtsprfigrrqsliedarkereaaaaaaaaa 59
                                   s p p+p+ + +a  e  ++ a    spr  gr+q   +d r+ r +a a aaaa
  tr|A0A2Z6ZTG8|A0A2Z6ZTG8_9LAMI 38 SGPRPDPRLLSQAALEALTRSAR-TDSPRRTGRKQFSGDDRRRRRRTAVAVAAAA 91
                                    8899*******99999888876.589*************999998888777776 PP

>> tr|I1JWI3|I1JWI3_SOYBN  AAI domain-containing protein OS=Glycine max OX=3847 GN=100817165 PE=4 SV=1
```

```
   #    score  bias  c-Evalue  i-Evalue hmmfrom  hmm to    alifrom  ali to    envfrom  env to     acc
 ---   ------ ----- --------- --------- ------- -------    ------- -------    ------- -------    ----
   1 ?   13.7   0.2   5.9e-05       9.2     308     341 ..      39      72 ..      23      92 .. 0.80

  Alignments for each domain:
  == domain 1  score: 13.7 bits;  conditional E-value: 5.9e-05
                sequence 308 vfqctqyirhasspmhspepdcchell.ghvpmla 341
                             + qc++y+ + s p  sp p+cche+     p l+
  tr|I1JWI3|I1JWI3_SOYBN  39 ITQCSEYVEK-SGPEMSPSPQCCHEIEnADTPCLC 72
                             679****976.78889*********9514566665 PP

>> tr|A0A0E0JL63|A0A0E0JL63_ORYPU  Uncharacterized protein OS=Oryza punctata OX=4537 PE=4 SV=1
   #    score  bias  c-Evalue  i-Evalue hmmfrom  hmm to    alifrom  ali to    envfrom  env to     acc
 ---   ------ ----- --------- --------- ------- -------    ------- -------    ------- -------    ----
   1 ?   13.1   0.0   9.1e-05        14     132     191 ..     213     272 ..     201     285 .. 0.86

  Alignments for each domain:
  == domain 1  score: 13.1 bits;  conditional E-value: 9.1e-05
                        sequence 132 fvrfevpsgdlaallssvrrvsddvrsaredkvpwfprkvseldkchhlvtkfdpdldld 191
                                     f  f+     +   l s++rv d+v s  ++k   f    + + kc  l   f pd d+
  tr|A0A0E0JL63|A0A0E0JL63_ORYPU 213 FTAFNFEKSVIEISLDSIKRVKDQVASETNQKCSTFDVVTAMMFKCRTLAIDFAPDADVR 272
                                     5556666666777899****************************************9974 PP

>> tr|A0A446NN00|A0A446NN00_TRITD  Uncharacterized protein OS=Triticum turgidum subsp. durum OX=4567 GN=TRITD_3Av1G18172
   #    score  bias  c-Evalue  i-Evalue hmmfrom  hmm to    alifrom  ali to    envfrom  env to     acc
 ---   ------ ----- --------- --------- ------- -------    ------- -------    ------- -------    ----
   1 ?   13.2   0.0   8.2e-05        13     309     362 ..      34      88 ..      12      95 .. 0.87

  Alignments for each domain:
  == domain 1  score: 13.2 bits;  conditional E-value: 8.2e-05
                        sequence 309 fqctqyirhasspmhspepdcche..llghvpmladrtfaqfsqdiglaslgasde 362
                                      qc q +rh    + sp    c+e  +lg v  +a+ t+ +f q  g+as+  sd
  tr|A0A446NN00|A0A446NN00_TRITD  34 KQCKQIVRHHLDSVTSPYSHICYEsdFLGGVGTVAN-TLHRFNQFSGVASFDLSDS 88
                                     59********************97558*****9996.889999999****999985 PP

>> tr|A0A445L060|A0A445L060_GLYSO  AAI domain-containing protein (Fragment) OS=Glycine soja OX=3848 GN=D0Y65_009689 PE=4
   #    score  bias  c-Evalue  i-Evalue hmmfrom  hmm to    alifrom  ali to    envfrom  env to     acc
 ---   ------ ----- --------- --------- ------- -------    ------- -------    ------- -------    ----
   1 ?   13.4   0.3   7.1e-05        11     308     341 ..      39      72 ..      31      91 .. 0.78

  Alignments for each domain:
  == domain 1  score: 13.4 bits;  conditional E-value: 7.1e-05
                        sequence 308 vfqctqyirhasspmhspepdcchell.ghvpmla 341
                                     + qc++y+ + s p  sp p+cche+     p l+
  tr|A0A445L060|A0A445L060_GLYSO  39 ITQCSEYVEK-SGPEMSPSPQCCHEIEnADTPCLC 72
                                     679****976.78889*********9514566665 PP
```

```
>> tr|J3L1W2|J3L1W2_ORYBR  Uncharacterized protein OS=Oryza brachyantha OX=4533 GN=102710161 PE=4 SV=1

   #    score  bias  c-Evalue  i-Evalue hmmfrom  hmm to    alifrom  ali to    envfrom  env to     acc

 ---   ------ ----- --------- --------- ------- -------    ------- -------    ------- -------    ----

   1 ?   13.0   0.0   9.5e-05        15     133     191 ..     215     273 ..     201     286 .. 0.85


  Alignments for each domain:

  == domain 1  score: 13.0 bits;  conditional E-value: 9.5e-05

                sequence 133 vrfevpsgdlaallssvrrvsddvrsaredkvpwfprkvseldkchhlvtkfdpdldld 191

                               f+     +   l s++rv d+v s  ++k   f    + + kc  l   f pd d+

  tr|J3L1W2|J3L1W2_ORYBR 215 TAFNFEKSVVEISLDSIKRVKDQVASETNQKCSTFDVVTAMMFKCRTLAIDFAPDADVR 273

                             455555666667899****************************************9974 PP




Internal pipeline statistics summary:

-------------------------------------

Query model(s):                            1  (498 nodes)

Target sequences:                   10161871  (3565606248 residues searched)

Passed MSV filter:                    296566  (0.0291842); expected 203237.4 (0.02)

Passed bias filter:                   227776  (0.0224148); expected 203237.4 (0.02)

Passed Vit filter:                     18333  (0.0018041); expected 10161.9 (0.001)

Passed Fwd filter:                       601  (5.91427e-05); expected 101.6 (1e-05)

Initial search space (Z):           10161871  [actual number of targets]

Domain search space  (domZ):              66  [number of targets reported over threshold]

# CPU time: 108.80u 1.97s 00:01:50.77 Elapsed: 00:00:44.76

# Mc/sec: 39664.42

//

[ok]
```

Supplementary File S8B. Protein domain similarity analysis using profile hidden Markov Models (hmmsearch).
